# Synthesis of 3-Carboxy-6-sulfamoylquinolones and Mefloquine-Based Compounds as Panx1 Blockers: Molecular Docking, Electrophysiological and Cell Culture Studies

**DOI:** 10.3390/molecules30102171

**Published:** 2025-05-15

**Authors:** Letizia Crocetti, Maria Paola Giovannoni, Tengis S. Pavlov, Veniamin Ivanov, Fabrizio Melani, Gabriella Guerrini

**Affiliations:** 1 Neurofarba, Pharmaceutical and Nutraceutical Section, University of Florence, Via Ugo Schiff 6, 50019 Sesto Fiorentino, Italy; letizia.crocetti@unifi.it (L.C.); mariapaola.giovannoni@unifi.it (M.P.G.); fabrizio.melani@unifi.it (F.M.); 2 Division of Hypertension and Vascular Research, Henry Ford Health & Wayne State University, 6135 Woodward Ave, Detroit, MI 48202, USA; tpavlov1@hfhs.org (T.S.P.); hr8703@wayne.edu (V.I.)

**Keywords:** Panx1, channel blockers, quinolones, patch clamp, molecular docking, multiple linear regression (MLR)

## Abstract

The membrane channel protein Panx1 is a promising therapeutic target since its involvement was demonstrated in a variety of pathologies such as neuropathic pain, ischemic stroke and cancer. As a continuation of our previous work in this field, we report here the synthesis and biological evaluation of two classes of compounds as Panx1 blockers: 3-carboxy-6-sulphonamidoquinolone derivatives and new Mefloquine analogs. The series of 3-carboxy-6-sulphonamidoquinolones gave interesting results, affording powerful Panx1 channel blockers with 73.2 < I% < 100 at 50 µM. In particular, **12f** was a more potent Panx1 blocker than the reference compound CBX (IC_50_ = 2.7 µM versus IC_50_ = 7.1 µM), and its profile was further investigated in a cell culture model of polycystic kidney disease. Finally, interesting results have been highlighted by new molecular modeling studies.

## 1. Introduction

Pannexins are a family of channel proteins present in the cellular membranes of vertebrates that connect the intra- and extracellular environment, thus allowing the passage of various molecules and ions. The three isoforms of this channel protein, called Panx1, Panx2 and Panx3 [1], identified in 2000, are expressed in various tissues and organs, but Panx1 proves to be the most abundant and the only one to be well characterized [2]. Panx1 shows a heptameric structure (see Figure S1, Supporting Information), and each subunit constituting the channel is equal and composed of four transmembrane alpha-helix domains (TM1-4), two extracellular loops (ECL1-2), an intracellular loop (ICL1) and two disulfide bridges (DS1-2) in the extracellular domain (ECD). The short amino-terminal group (N-terminal helices, NTH) of the hPanx1 channel protrudes into the transmembrane domain (TMD), and each NTH of one subunit lines the TMD of the adjacent ones [3], differently from the frog Panx1 channel in which NTH is located in the intracellular domain [4]. The carboxy-terminal group delimits the channel pore funnel but, depending on the construction strategies for cryo-EM (truncation or cleavage by caspases or C-terminal tagging), the C-terminal helix (CTH) segments were not well identified and could be exposed in the cytoplasm [5,6], so the elucidation of the full mechanism of the channel is incomplete. Recently, Hussain and coworkers [7], using the cryo-EM technique, identified the different structural organizations of Panx1-3, which show three different residues (W74, R89 and I74, respectively) in the extracellular domain, responsible for the different dimensions of the channel mouth. It has been known for several years that Panx1 activation results in the passage of ATP across the membrane [8], thus increasing extracellular ATP levels [9]. In addition, Panx1 seems to be subject to negative feedback regulation by elevated concentrations of ATP, whose binding to extracellular binding sites is responsible for blocking Panx1 currents and ATP release, as well as for inducing Panx1 internalization [10,11]. The involvement of these channel proteins in numerous pathologies has been highlighted, and these diseases are generally related to overexpression or up-regulation of Panx1. The most-studied pathologies are CNS diseases, such as ischaemic stroke, epilepsy, neuroinflammation and Parkinson’s disease, but also cancer and neuropathic pain, thus opening an interesting perspective on the use of channel blockers for their resolution [12,13]. Recently, Pavlov and coworkers [14] also demonstrated in a murine model the involvement of Panx1 channels in Autosomal Dominant Polycystic Kidney Disease (ADPKD), a monogenic disorder caused by *PDK1* and/or *PDK2* mutations, which result in the development of multiple cysts overexpressing Panx1 channels across the nephron [15]. The treatment of mice with the non-selective Panx1 blocker Probenecid reduced ATP release and slowed renal cyst development by improving epithelial transport across the cyst epithelium, thus demonstrating that Panx1 could be a new target for the treatment of ADPKD [14].

The first medicinal chemistry papers on this target have been published by our research group in recent years and report the results of the investigation of various heterocyclic nitrogen nuclei, including the quinoline scaffold [16,17,18], which is a significant assembly motif for the development of new drugs [19].

**Figure 1:**
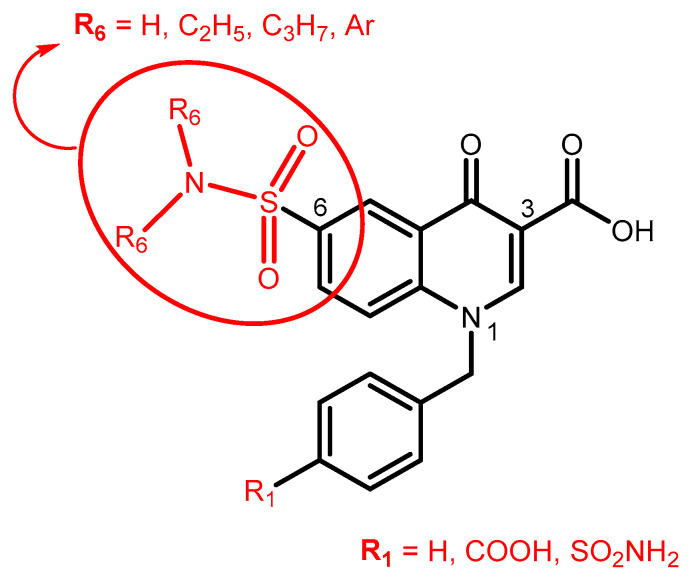
The first aim of this work: further elaboration of quinolone-based compounds already published by us.

Still working on the quinoline scaffold, in this new manuscript, we address two different aims. On the one hand, we have further developed the already-published 3-carboxy-6-nitroquinolone compounds [18] by introducing a sulphonamide function substituted (red circle in Figure 1) at position 6 of the scaffold (till now never inserted), while position 1 contains the best substituents of previously reported compounds (4-carboxy-, 4-sulfamoylbenzyl or benzyl groups; Figure 1). The choice of the sulphonamide function at position 6 of the quinolone scaffold (till now never inserted) is due to the evidence that this group results in a significant increase in activity in a series of indoles previously published by us [16].

**Figure 2:**
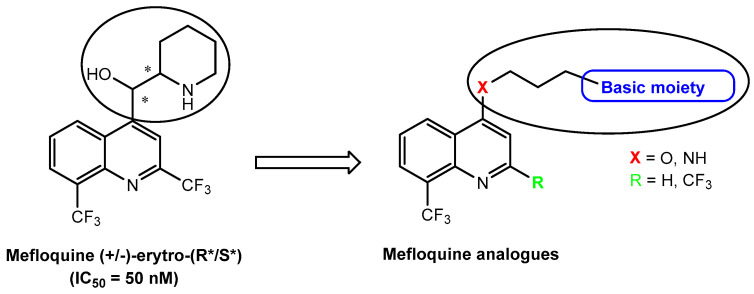
The second aim of the work: Mefloquine-based compounds as Panx1 blockers. The asterisks indicate the asimmetric carbons.

On the other hand, we have planned the synthesis of new analogs of Mefloquine, a well-known antimalarial agent but also a potent and selective Panx1 inhibitor (IC_50_ = 50 nM) [20]. As regards the Mefloquine-based compounds, we first investigated the importance of the basic portion of the drug for Panx1 blocker activity by replacing the (piperidin-2-yl)methanol moiety of Mefloquine with other basic groups linked at position 4 of the quinoline scaffold through an oxygen or NH spacer; some compounds lacking CF_3_ in position 2 have also been synthesized (Figure 2). All the final compounds have been tested as Panx1 blockers with a patch clamp on a Chinese hamster ovarian (CHO) cell heterologous expression system. We rationalized the in vitro results, and molecular modelling studies and chemometric analysis were performed. Finally, two representative products **12e** and **12f** were tested in a cell culture model of polycystic kidney disease.

## 2. Results and Discussions

### 2.1. Chemistry

**Scheme 1:**
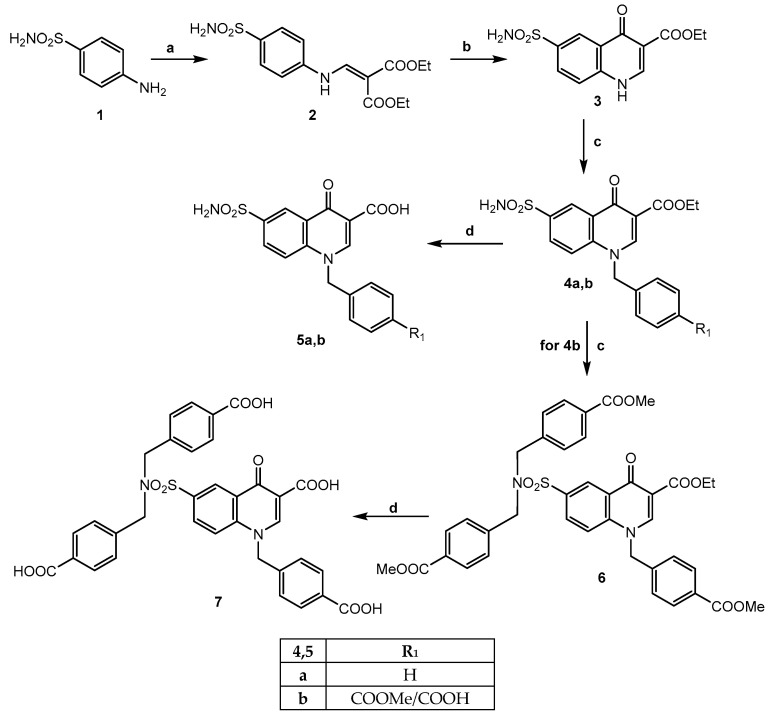
**Reagents and conditions:** (**a**) diethyl ethoxymethylenmalonate, 120 °C, 3 h (yield 86%); (**b**) Eaton’s reagent, 85 °C, 2 h (yield 23%); (**c**) K_2_CO_3_, DMF dry, r.t., 30′; suitable R-benzylbromide, 80 °C, 2 h (yield 30%); (**d**) for **5a**,**b**: EtOH/NaOH 10% (1:1), reflux, 30′. For **7**: NaOH 40%, EtOH (drops), reflux, 2 h (yield 70–80%).

**Scheme 2:**
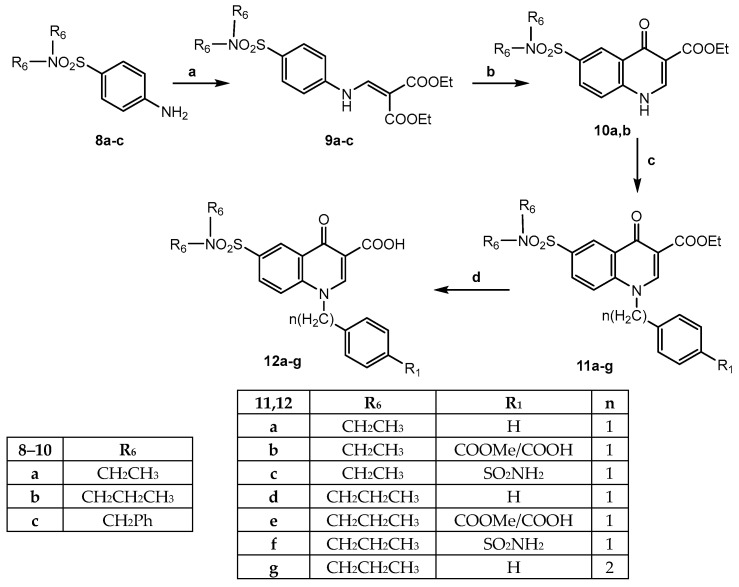
**Reagents and conditions:** (**a**) diethyl ethoxymethylenmalonate, 1,4-dioxane, reflux, 4 h (yield 40–60%); (**b**) Eaton’s reagent, 100 °C, 3 h (yield 68%) (for **9c**, closure failed); (**c**) K_2_CO_3_, DMF dry, r.t., 30′; suitable R-benzylbromide, 80–120 °C, 2–3 h (yield 20–30%); (**d**) EtOH/NaOH 10% (1:1), reflux, 30′ (yield 70–90%).

**Scheme 3:**
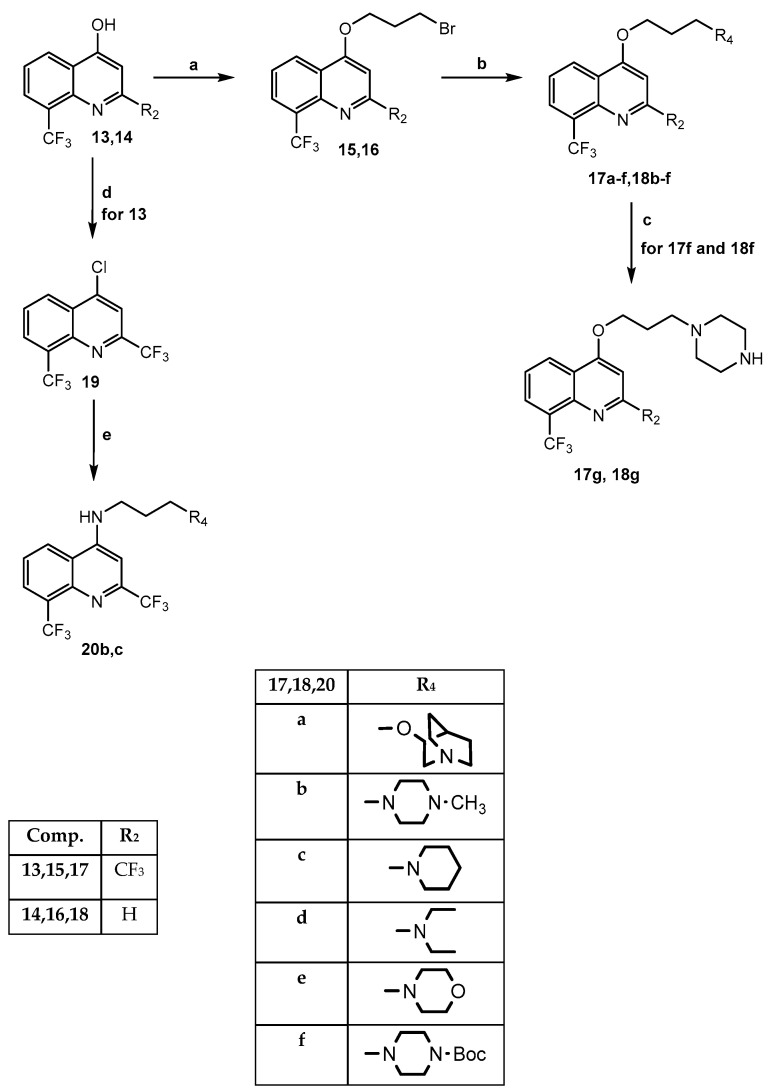
**Reagents and conditions:** (**a**) DMF dry, K_2_CO_3_, r.t., 30 min; 1,3-dibromopropane, 70 °C, 5 h (yield 30–40%); (**b**) DMF dry, K_2_CO_3_, suitable reactive, r.t., 24–72 h (yield 30–70%); (**c**) DCM/CF_3_COOH 6:1, r.t., 2 h (yield 80%); (**d**) POCl_3_, 80/90 °C, 2–5 h (yield 50%); (**e**) EtOH abs, suitable amine, DIPEA, 90 °C, 14 h (yield 20–25%).

The procedures followed to obtain all new products are shown in Scheme 1 and Scheme 2 (3-carboxy quinolone derivatives) and Scheme 3 (Mefloquine-based compounds), and the structures were confirmed by analytical and spectral data (mono- and bidimensional NMR spectra). The reagents used in the synthetic pathways are commercially available or were synthesized following the procedures reported in the literature (see Supporting Information). Scheme 1 shows the procedure for obtaining the final products **5a**,**b** and **7**. By reacting the commercial 4-aminobenzenesulphonamide **1** with diethyl ethoxymethylenmalonate at 120 °C for 3 h, (diethyl 2-{[(4-sulfamoyl phenyl) amino]methylene}malonate) **2** [21] was obtained; this is a useful intermediate which easily cyclizes with Eaton’s reagent to yield ethyl 4-oxo-6-sulfamoyl-1,4-dihydroquinoline-3-carboxylate **3**. Under usual conditions (DMF, K_2_CO_3_ and (substituted)benzylbromide), compound **3** was alkylated to obtain the N-benzyl derivatives **4a**,**b**. Reacting compound **4b** with an excess of methyl-4-(bromomethyl)benzoate [22], the tertiary sulphonamide **6** was obtained. All esters **4a**,**b** and **6** were finally converted into their respective carboxylic acids **5a**,**b** and **7** by hydrolysis in a 10% sodium hydroxide solution and ethanol 96%.

Scheme 2 reports the synthesis of the final acid compounds of type **12**, bearing the tertiary diethyl or dipropyl sulphonamide at position 6 of the quinolinone scaffold. The starting products **8a** [23], **8b** [24] and **8c**, synthesized following the procedures reported in the literature [25], were reacted with diethyl ethoxymethylenmalonate, as reported in Scheme 1, to obtain compounds **9a**–**c** [23]. The next cyclization with Eaton’s reagent to obtain compounds of type **10** took place for **9a** and **9b** only, giving **10a** [23] and **10b**, respectively, whereas for the intermediate **9c**, the cyclization to a quinolone nucleus did not occur, but rather, primary sulfonamide **2** (see Scheme 1) formation by N-debenzylation was observed. The further alkylation on **10a**,**b** performed in conditions previously reported (DMF and K_2_CO_3_) using benzylbromide, methyl-4-(bromomethyl)benzoate, 4-bromomethylbenzenesulfonamide and (2-bromoethyl)benzene gave the N-alkylated quinolinones (**11a**–**g**), whose ester functions were hydrolyzed, and the final corresponding acids **12a**–**g** were easily obtained.

Moving on to Mefloquine-based compounds, the synthesis is depicted in Scheme 3. In particular, the starting materials 2,8-bis-trifluoromethyl-4-hydroxyquinoline **13** [26] and 8-trifluoromethyl-4-hydroxyquinoline **14** [27] underwent *O*-alkylation with 1,3-dibromopropane in dry DMF/K_2_CO_3_, yielding the key intermediates **15** and **16**, which were reacted with the appropriate nucleophile reagent [(cyclo)alkylamine or quinuclidinol] under standard conditions, and the final products **17a**–**f** and **18b**–**f** were obtained (**17c**,**e** [28]). Compounds **17f** and **18f**, bearing the 3-(piperazin-1-Boc)propoxy- group at position 4 of the quinoline scaffold, were transformed into **17g** and **18g** by cleavage of the Boc group with a mixture of DCM and trifluoroacetic acid (6:1). To obtain the final 4-amino derivatives **20b**,**c** (isosters of **17b** and **17c**, respectively), the synthetic strategy provided the formation of 4-chloro-2,8-bis(trifluoromethyl) quinoline **19** [26] from intermediate **13** by reacting with phosphorus oxychloride in the usual conditions, followed by nucleophilic substitution with the suitable amine.

Due to the possible tautomerism of the key intermediates **13** and **14**, we used ^13^C-NMR and two-dimensional NMR techniques (^1^H-^13^C-HSQC (Heteronuclear Single-Quantum Correlation) and ^1^H-^13^C-HMBC (Heteronuclear Multiple-Bond Correlation)) to assign the correct structure to products of types **15** and **16** (*N*- or *O*-alkylated) obtained by alkylation of **13** and **14**. Both compounds of types **15** and **16** in the ^13^C-NMR spectra show the signal of the C-4 quaternary carbon at about 160 ppm, different from the ketone signal, which usually falls higher, at about 170 ppm (see Supporting Information). In addition, from the HMBC spectrum of both products, a coupling between the C-4 carbon and the methylene protons of the O-CH_2_- chain was evident (see Supporting Information). Thus, confirming that the alkylations occur at 4-OH and not at N-1, consistently, we attributed the structures **15** and **16** drawn in Scheme 3 to the alkylation products.

### 2.2. Biological Results

All new products were tested in mammalian CHO cells transfected with mPanx1 cDNA plasmids through patch-clamp analysis at 50 µM. Additionally, dose-dependency and IC_50_ were determined for the best three compounds. The voltage-clamp protocol included serial ramping from 90 to −90 mV for the cells in a whole-cell configuration to elicit pannein-1-dependent currents (see Supporting Information). Carbenoxolone (CBX) used as a reference compound gave an inhibition of 76% at 15 µM and an IC_50_ = 7.1 µM, in agreement with data reported in the literature [18,29]. Of the two targets we had set for this work, namely the synthesis of 3-carboxy-6-sulphonamidequinolones and Mefloquine-based compounds, only the first one performed well, as the Mefloquine derivatives of types **17** and **18** turned out to have very low activity or be completely inactive at the dose tested (I% < 20%). In contrast, the new 6-sulphonamide quinolones **5a**,**b**, **7**, and **12a**–**g** show interesting results reported in Table 1, together with CBX and the two reference products **6a** and **6g** (original numbering) previously published by us [18] of which the new products are elaborations. Analysis of the biological data led us to consider two main elements important for the activity: both the sulphonamide function and the acid group at R_1_. The replacement of the reference compounds **6a** and **6g** of the nitro group at position 6 with a primary sulphonamide function (compounds **5a** and **5b**) appears detrimental for activity, but in agreement with the couple **6a/6g** for **5a/5b**, the carboxy group in the para position of the benzyl improves the channel inhibition by about two-fold [I% = 27.1 and 48.8 for **5a** (R_1_ = H) and **5b** (R_1_ = COOH), respectively]. Moving from the primary to the tertiary sulphonamide (compounds of type **12**), a marked increase in channel inhibition was observed; also for these products, the introduction of the acid group at R_1_ was favorable for activity as for (N,N-diethylsulfamoyl) derivatives **12a** (R_1_ = H) and **12b** (R_1_ = COOH) with I% = 54.6 and 73.2, respectively, as well as for the N,N-dipropylsulfamoyl **12d** (I% = 10.4) and its corresponding acid derivatives **12e** (I% = 84.8) and **12f** (I% = 100). On the other hand, the introduction of a benzyl of a different acid function, such as a primary sulphonamide (compounds **12c** and **12f**), in the para position gives different results. In fact, in the first case, it provides a less potent product (compound **12c**, I% = 49.5) not only with respect to the carboxy analog (compound **12b**, I% = 73.2) but also to the unsubstituted one (compound **12a**, I% = 54.6); in contrast, for compound **12f**, a complete block of the channel was recorded (I% = 100), giving the most potent compounds in this research. The only product (**12g**) in which we elongated the methylene spacer between N-1 and phenyl (n = 2) was considerably more potent (I% = 57.2) than its analog with n = 1 (**12d**, I% = 10.4), thus opening up a future possibility for further study. Finally, the N,N-di(4-carboxybenzyl)sulphonamide derivative **7** is a potent Panx1 channel blocker with I% = 79.7; these data are in agreement with results obtained on the indole scaffold already published by us [16] and suggest that the favorable effect of lipophilic and bulky groups at this position is due to their ability to block the channel for steric hindrance, as with CBX.

**Figure 3:**
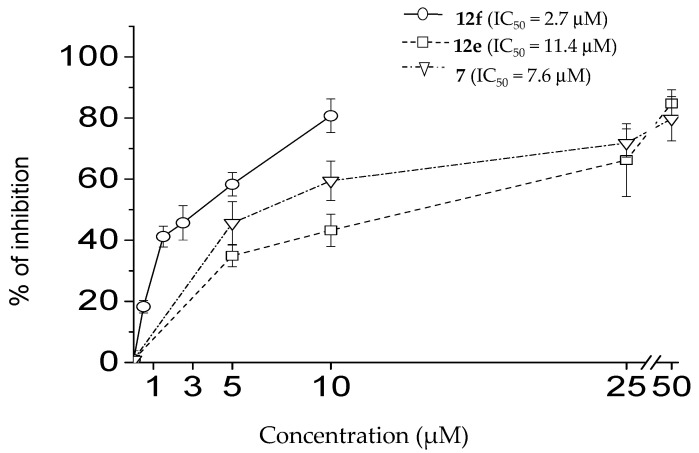
Dose-dependent effect of **12f**, **12e** and **7** on mPanx1 current. Percentage of inhibition (mean ± SEM) of patch-clamp current via pannexin-1 reconstituted in CHO cells.

For the most potent compounds **7**, **12e** and **12f** (79.6 < I% < 100), dose–response curves were established (Figure 3), giving IC_50_ values in the low micromolar range, and the values for **12f** were lower than the reference compound CBX (IC_50_ = 2.7 µM versus IC_50_ = 7.1 µM).

**Figure 4:**
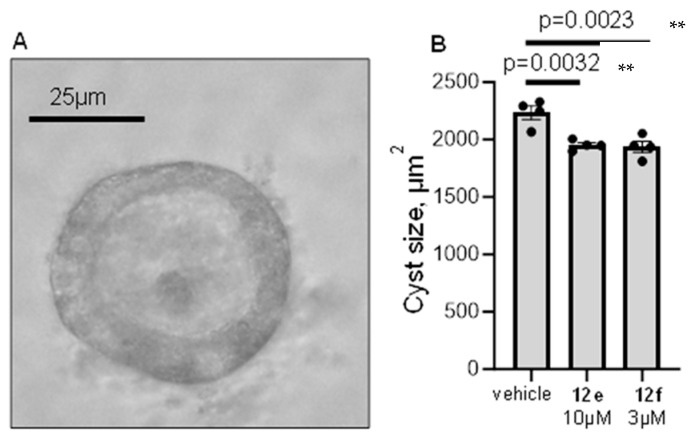
Effect of treatment with RF4 and RF6 on a model of polycystic kidney disease. Mouse renal epithelial cells were grown in the presence of forskolin to induce the formation of 3D cysts. (**A**)—representative image of a cyst-like spheroid formed by renal epithelial cells. (**B**)—the size of spheroids in different treatment groups. Each dot represents the mean size of 50+ cysts grown in a well of a 12-well plate. N = 4 wells/group; ** *p* < 0.01 according to the Dunnett test.

The therapeutic properties of compounds **12e** and **12f** were further tested in a model of renal cyst development. In fact, we demonstrated earlier that 50 µM Probenecid, a non-selective Panx1 blocker, was effective in a murine model of polycystic kidney disease, precluding the formation of renal cysts [14]. For this study, we used mpkCCD_cl4_ cell culture derived from mouse distal nephron, which forms cyst-like spheroids if grown in 3D Matrigel in the presence of forskolin. The formation of cysts and their size in this model depend on Panx1 activity [14,30]. Figure 4 demonstrates that the presence of 10 µM **12e** or 3 µM **12f** in the media during the growth period (3 weeks) precludes cyst development (vehicle 2235 ± 58 µm^2^; **12e** 1951 ± 22 µm^2^; **12f** 1937 ± 49 µm^2^). Since doses near IC_50_ were used in this experiment, we believe that the treatment of animals with **12e** and **12f** in submaximal doses could have high therapeutic potential as a pre-clinical research strategy

### 2.3. Molecular Modeling

**Figure 5:**
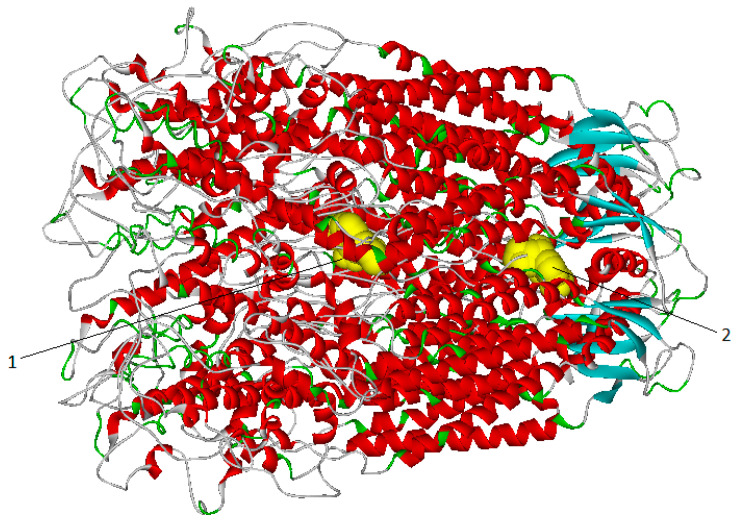
Distribution of the positions of Mefloquine in the whole channel: 35% of the positions located in the channel (1); 65% of positions located in the ECD (2).

To rationalize the biological results, a molecular modeling study on the Panx-1 channel obtained by cryo-EM (PDB 7DWB) [3] was performed. The 7DWB channel structure was chosen because it is the most complete among those deposited. In this study, the hydrogen bonds between the newly synthesized molecules and the amino acids present in the human Panx-1 channel were chosen on the assumption that they are primarily responsible for the strength and topology of the binding. The hydrogen bonds were characterized by their length, which first approximation can be considered inversely proportional to the strength of the bond. The location of possible docking sites on the entire channel was searched with AutoDock4 [31], using the Mefloquine (Mef) molecule. Two possible docking sites were identified, the most probable of which (65% of the poses) were located in the ECD portion of the channel (Figure 5).

Based on the behavior observed with Mefloquine and considering it unlikely that a larger molecule could insert itself deeply into the channel, due to various constrictions present in the length of the channel [6], it is believed that the ECD portion is where inhibitor binding is most likely to occur. So, using AutoDock 4.2 [31] (the operating conditions are reported in Supporting Information), the newly synthesized molecules were induced to bind with the protein in the ECD portion. Docking was performed with the rigid protein and the positions produced by AutoDock (i.e., the conformations of the ligand–protein complex) were collected in clusters with a structural homogeneity of within 10 RMS. For each cluster, the pose with the best binding energy of the largest cluster was considered for the following calculations, and in cases of low numbers, more clusters were considered in order to represent at least 70% of the positions produced by AutoDock (see Tables S1 and S2 in Supporting Information; compounds with more conformations are indicated as ‘compound’s number_n’). Ligand–protein complexes were solvated and minimized with GROMACS [32] (operating conditions are reported in the Supporting Information). On the minimized ligand–protein complex structures, all possible hydrogen bonds within a distance of 3 Å between the ligands and the protein amino acids were detected (Table S1) and between the ligands and the water molecule that simultaneously forms a hydrogen bond with an amino acid of the protein, always within a distance of 3 Å (Table S2).

Since the Panx-1 channel is formed by seven identical chains, the amino acids reported in the tables do not refer to a specific chain. The ligands bearing carboxylic acid group(s) were also considered in their deprotonated form and are indicated in the tables as superscript minus (^−^). The tables report the percentage inhibition value (I%) of the current, evaluated by patch-clamp experiments on the channel transfected with Panx1.

To search for a correlation between the length of hydrogen bonds (i.e., the strength of the hydrogen bond) and the value of percentage inhibition (I%), a multiple linear regression (MLR) was used. The independent variables (V) are the length of hydrogen bonds with the amino acids and the “bridged” water molecules. The 31 independent variables (V) are reported in Table 2, where the amino acids bound to the ligands with a “bridged” water molecule are indicated with the prefix W.

The MLR was set up with only eight independent variables (about a quarter of the total number of measured variables). A higher number of variables would lead to overfitting; a lower number would result in barely significant correlations.

**Figure 6:**
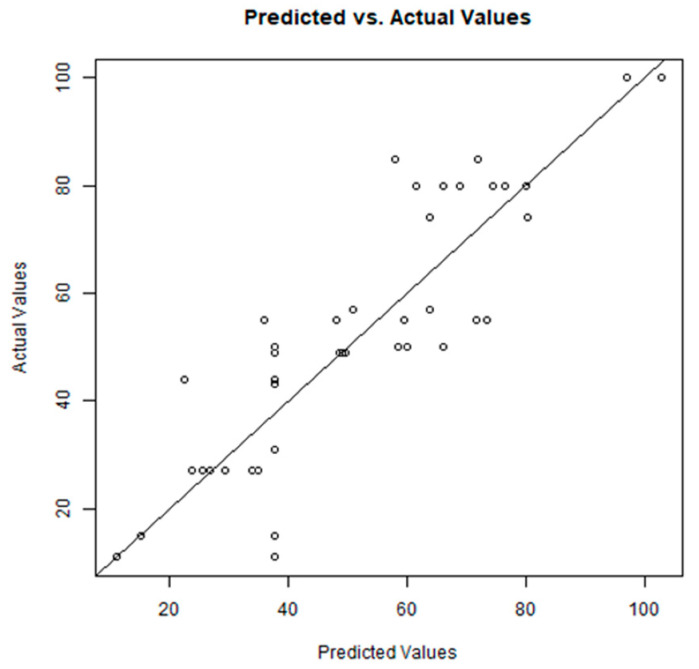
Relationship between the predicted value and the actual value from biological studies. R2 (determination coefficient) = 0.746; RMSE (Root Mean Square Error) = 12.226; MAE (Mean Absolute Error) = 9.429.

To select the combinations of the eight independent variables that provide the best correlation, a genetic algorithm (GA) was employed, and the flowchart and operating conditions of the GA used are reported in the Supporting Information. The GA uses the MLR as a fitness function and the determination coefficient (R2) as the score value. The combination of the variables V8, V19, V24, V27, V17, V26, V11 and V10 resulted in a determination coefficient of 0.746. The R2 value of 0.746, for the best combination of variables, is significant enough to suggest the existence of a correlation between hydrogen bond distances (“hydrogen bond strength”) between ligands and amino acids and percentage inhibition (I%). The correlation is graphically represented in Figure 6. The RMSE value indicates that the percentage inhibition (I%) calculated by the model (predicted value) differs from the actual value only by ±12%. The actual and predicted values of I% and the absolute errors are reported in the Supporting Information.

It is important to underline that the models obtained are not validated, that is, they were calculated in fitting; therefore, the results have a descriptive and not predictive value. The sign of the regression coefficients (positive or negative; see Table 3) allows for the identification of the type of contribution to activity made by the amino acid or by the amino acid when bound to a bridging water molecule.

**Figure 7:**
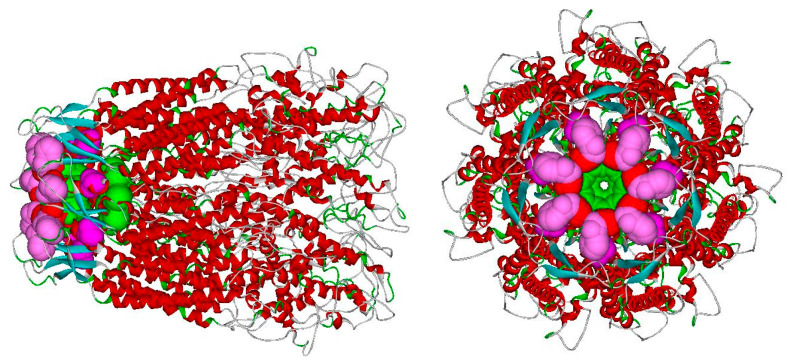
Significant amino acids for activity in the human Panx1 channel (see text).

The negative sign (red) of the coefficients for the variables V10 and V17 indicates that to have activity, the length of the hydrogen bonds with Trp74 and Cys434 must be as short as possible (strong hydrogen bond). In contrast, the positive sign of the coefficients for the variables V24, V26 and V8 indicates that to have activity, the length of the hydrogen bonds must be as long as possible (weak hydrogen bond) with the bridged water of Ser73, Arg75 and directly with Phe72, that is, the ligand must not interact with these amino acids. From a topological point of view, it emerges that the amino acids Cys434 and Trp74 (in green) are located in the deepest part of the ECD, while the amino acids Ser73, Arg75 and Phe72 (red, dark pink and pink) are located in the initial and external parts of the channel, as shown in Figure 7. This suggests that to have a high inhibition percentage value, the molecule must be inserted deeply into the ECD of the channel (in the ‘green zone’).

**Figure 8:**
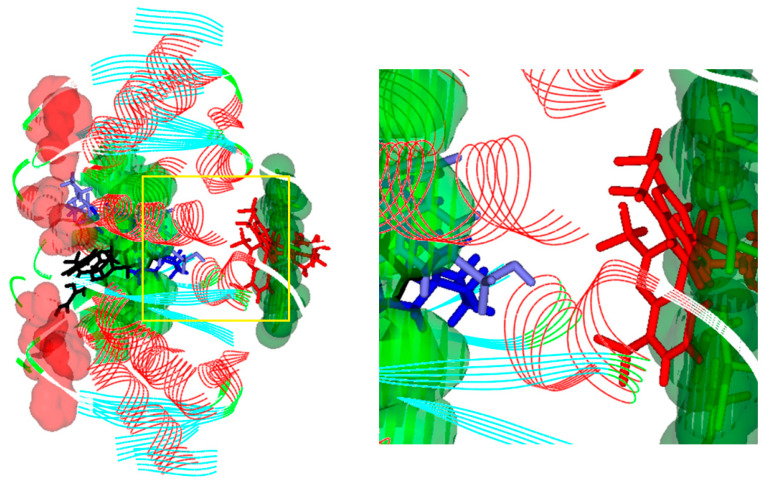
ECD portion of the 7DWB channel. Amino acids are represented with a VDW surface: Cys434 in green, Trp74 in light green and Phe72 in red. Molecules are represented in sticks: Mefloquine in red, **12e** in blue, **12f** in light blue and **12d** in black.

From Figure 8, where the EDC portion of the 7DWB channel is shown, it can be seen how the Mefloquine molecule (red) is deeply inserted into the channel, while the most active molecules **12e** (blue) and **12f** (light blue) are placed in an intermediate position between Mefloquine and the inactive molecule **12d** (black), which is positioned more externally than **12e** and **12f** and cannot be inserted as deeply. As previously suggested, the deeper the molecules are positioned, the more active they become. Among the four considered in the figure, Mefloquine is the most active, and **12e** and **12f** exhibit sufficient activity, while **12d**, which is positioned more externally than the others and has no deep ramifications, is practically inactive.

## 3. Materials and Methods

All melting points were determined on a Büchi apparatus (New Castle, DE, USA). Extracts were dried over Na_2_SO_4_, and the solvents were removed under reduced pressure. Merck F-254 commercial plates (Merck, Durham, NC, USA) were used for analytical TLC to follow the course of reactions. Silica gel 60 (Merck 70–230 mesh, Merck, Durham, NC, USA) was used for column chromatography. ^1^H-NMR, ^13^C-NMR, HSQC and HMBC bidimensional spectra were recorded on an Avance 400 instrument (Bruker Biospin Version 002 with SGU, Bruker Inc., Billerica, MA, USA). Chemical shifts (δ) are reported in ppm to the nearest 0.01 ppm using the solvent as an internal standard. Coupling constants (J values) are given in Hz and were calculated using TopSpin 1.3 software (Nicolet Instrument Corp., Madison, WI, USA), rounded to the nearest 0.1 vHz. Data are reported as follows: chemical shift, multiplicity (exch, exchange; br, broad; s, singlet; d, doublet; t, triplet; q, quartet; m, multiplet; or a combination of those, e.g., dd), integral, assignments, and coupling constant. Mass spectra (*m*/*z*) were recorded on an ESI-MS triple quadrupole (Varian 1200L) system, in positive ion mode, by infusing a 10 mg/L solution of each analyte dissolved in a mixture of mQ H_2_O/acetonitrile 1:1 *v*/*v*. All new compounds had a purity >95%; microanalyses indicated by symbols of the elements were performed on a Perkin-Elmer 260 elemental analyzer (Waltham, MA, USA) for C, H and N, and they were within ± 0.4% of the theoretical values.

### 3.1. Chemistry

*Diethyl 2-{[(4-sulfamoylphenyl)amino]methylene}malonate* (**2**): 2.90 mmol (500 mg) of 4-aminobenzenesulphonamide **1** (commercially available) and 2.90 mmol (0.6 mL) of diethylethoxymethylen malonate (commercially available) were stirred at 120 °C for 3 h. After cooling at room temperature, a white solid was formed and recovered with small amounts of ethanol by *vacuum* filtration to obtain the desired compound. Yield = 86%; mp = 153–155 °C (EtOH). ^1^H-NMR (400 MHz, DMSO-*d*_6_) δ 1.25 (t, 6H, *J* = 6.8 Hz, 2 × OCH_2_*CH_3_*); 4.15 (q, 2H, *J* = 7.0 Hz, O*CH_2_*CH_3_); 4.23 (q, 2H, *J* = 7.0 Hz, O*CH_2_*CH_3_); 7.32 (exch br s, 2H, SO_2_NH_2_); 7.55 (d, 2H, *J* = 8.8 Hz, Ar); 7.79 (d, 2H, *J* = 8.4 Hz, Ar); 8.41 (d, 1H, *J* = 13.2 Hz, C=*CH*); 10.72 (exch br d, 1H, *J* = 13.6 Hz, NH). Anal. C_14_H_18_N_2_O_6_S (C, H, N) calcd. C, 49.12; H, 5.30; N, 8.18; found: C, 49.81; H, 5.32; N, 8.21 [21].

*Ethyl 4-oxo-6-sulfamoyl-1,4-dihydroquinoline-3-carboxylate* (**3**). To 2.02 mmol of intermediate **2**, 5 mL (26.26 mmol) of Eaton’s reagent was added and the mixture was heated at 85 °C for 2 h. After cooling at room temperature, the mixture was slowly added drop by drop to a saturated solution of NaHCO_3_ (30 mL), and the formation of an orange precipitate was observed. The solid was recovered by *vacuum* filtration to obtain the desired compound. Yield = 23%; mp > 300 °C (EtOH). ^1^H-NMR (400 MHz, DMSO-*d*_6_) δ 1.29 (t, 3H, *J* = 6.8 Hz, OCH_2_*CH_3_*); 4.23 (q, 2H, *J* = 6.8 Hz, O*CH_2_*CH_3_); 7.51 (exch br s, 2H, SO_2_NH_2_); 7.79 (d, 1H, *J* = 8.4 Hz, Ar); 8.06 (d, 1H, *J* = 8.4 Hz, Ar); 8.62 (d, 2H, *J* = 9.2 Hz, Ar). Anal. C_12_H_12_N_2_O_5_S (C, H, N) calcd. C, 48.64; H, 4.08; N, 9.45; found: C, 48.83; H, 4.10; N, 9.49.

*General procedure for the synthesis of compounds* **4a**,**b**. A mixture of intermediate **3** (0.61 mmol) and anhydrous K_2_CO_3_ (1.22 mmol) in 8 mL of dry DMF was stirred at room temperature for 30 min. Then, the appropriate aromatic halide (0.92 mmol) was added, and the mixture was stirred at 80–90 °C for 2–3 h. After cooling, the mixture was concentrated in vacuo, and ice-cold water (10 mL) was added. The suspension was extracted with ethyl acetate (3 × 15 mL), dried on sodium sulphate and evaporated. The crude product was purified by flash column chromatography using ethyl acetate as the eluent.

*Ethyl 1-benzyl-4-oxo-6-sulfamoyl-1,4-dihydroquinoline-3-carboxylate* (**4a**). Benzyl bromide (commercially available) was used as the reagent. Yield = 30%; mp = 142–145 °C (EtOH). ^1^H-NMR (400 MHz, CDCl_3_) δ 1.41 (t, 3H, *J* = 7.2 Hz OCH_2_*CH_3_*); 4.40 (q, 2H, *J* = 7.2 Hz, O*CH_2_*CH_3_); 5.41 (s, 2H, N-*CH_2_*Ph); 7.15–7.40 (m, 7H, 5H Ar + 2H SO_2_NH_2_); 8.10 (dd, 1H, *J*_1_ = 8.8 Hz, *J*_2_ = 2.0 Hz, Ar); 8.18 (s, 1H, Ar); 8.61 (s, 1H, Ar); 8.88 (ds, 1H, *J* = 2.0 Hz, Ar). Anal. C_19_H_18_N_2_O_5_S (C, H, N) calcd. C, 59.06; H, 4.70; N, 7.25; found: C, 59.29; H, 4.72; N, 7.28.

*Ethyl-1-[4-(methoxycarbonyl)benzyl]-4-oxo-6-sulfamoyl-1,4-dihydroquinoline-3-carboxylate* (**4b**). Methyl 4-(bromomethyl)benzoate [22] was used as the reagent. Yield = 30%; mp = 217–220 °C (EtOH). ^1^H-NMR (400 MHz, DMSO-*d*_6_) δ 1.31 (t, 3H, *J* = 7.2 Hz, OCH_2_*CH_3_*); 3.83 (s, 3H, O*CH_3_*); 4.27 (q, 2H, *J* = 7.2 Hz, O*CH_2_*CH_3_); 5.83 (s, 2H, N-*CH_2_*Ph); 7.37 (d, 2H, *J* = 8.0 Hz, Ar); 7.50 (exch br s, 2H, SO_2_NH_2_); 7.72 (d, 1H, *J* = 9.2 Hz, Ar); 7.94 (d, 2H, *J* = 8.0 Hz, Ar); 7.98 (dd, 1H, *J*_1_ = 9.0 Hz, *J*_2_ = 2.0 Hz, Ar); 8.68 (ds, 1H, *J* = 2.0 Hz, Ar); 9.01 (s, 1H, Ar). Anal. C_21_H_20_N_2_O_7_S (C, H, N) calcd. C, 56.75; H, 4.54; N, 6.30; found: C, 56.52; H, 4.52; N, 6.27.

*General procedure for the synthesis of acids* **5a**,**b**. A mixture of suitable esters of type **4** (0.18 mmol), NaOH 10% (1.5 mL) and EtOH 96% (1.5 mL) was stirred at reflux for 30 min. After cooling, ice-cold water was added, the mixture was acidified with HCl 6M, and the precipitate was recovered by vacuum filtration and recrystallized from ethanol.

*1-Benzyl-4-oxo-6-sulfamoyl-1,4-dihydroquinoline-3-carboxylic acid* (**5a**). Yield = 77%; mp > 300 °C (EtOH). ^1^H-NMR (400 MHz, DMSO-*d*_6_) δ 5.91 (s, 2H, N-*CH_2_*Ph); 7.28–7.39 (m, 5H, Ar); 7.60 (exch br s, 2H, SO_2_NH_2_); 8.04 (d, 1H, *J* = 8.8 Hz, Ar); 8.16 (dd, 1H, *J*_1_ = 8.8 Hz, *J*_2_ = 2.0 Hz, Ar); 8.79 (s, 1H, Ar); 9.35 (s, 1H, Ar); 14.71 (exch br s, 1H, COOH). ^13^C-NMR (100 MHz, DMSO-*d*_6_) δ 57.1; 109.5; 120.6; 124.2; 126.0; 127.1; 128.6; 129.4; 130.9; 135.5; 141.6; 141.9; 151.8; 165.9; 178.2. ESI-MS calcd. for C_17_H_14_N_2_O_5_S, 358.37; found: *m*/*z* 359.07 [M + H]^+^. Anal. C_17_H_14_N_2_O_5_S (C, H, N) calcd. C, 56.98; H, 3.94; N, 7.82; found: C, 56.75; H, 3.92; N, 7.79.

*1-(4-Carboxybenzyl)-4-oxo-6-sulfamoyl-1,4-dihydroquinoline-3-carboxylic acid* (**5b**). Yield = 76%; mp = 215–218 °C (EtOH). ^1^H-NMR (400 MHz, DMSO-*d*_6_) δ 5.98 (s, 2H, N-*CH_2_*Ph); 7.35 (d, 2H, *J* = 8.4 Hz, Ar); 7.59 (exch br s, 2H, SO_2_NH_2_); 7.90 (d, 2H, *J* = 8.4 Hz, Ar); 7.97 (d, 1H, *J* = 9.2 Hz, Ar); 8.15 (dd, 1H, *J*_1_ = 9.0 Hz, *J*_2_ = 2.2 Hz, Ar); 8.79 (s, 1H, Ar); 9.36 (s, 1H, Ar); 13.50 (exch br s, 1H, COOH); 14.71 (exch br s, 1H, COOH). ^13^C-NMR (100 MHz, DMSO-*d*_6_) δ 56.9; 109.7; 120.5; 124.3; 126.0; 126.9; 130.3; 130.9; 139.8; 141.5; 141.9; 152.0; 165.9; 167.6; 178.2. ESI-MS calcd. for C_18_H_14_N_2_O_7_S, 402.38; found: *m*/*z* 403.06 [M + H]^+^. Anal. C_18_H_14_N_2_O_7_S (C, H, N) calcd. C, 53.73; H, 3.51; N, 6.96; found: C, 53.94; H, 3.52; N, 6.99.

*Ethyl-6-{N-(4-acetoxybenzyl)-N-[4-(methoxycarbonyl)benzyl]sulfamoyl}-1-(4-(methoxycarbonyl)benzyl)-4-oxo-1,4-dihydroquinoline-3-carboxylate* (**6**). Compound **6** was obtained following the same synthetic procedure and the same number of equivalents used for compounds **4a**,**b**, but starting from intermediate **4b** and using the appropriate reagent. The crude compound was purified by flash column chromatography using ethyl acetate as the eluent. Yield = 8%; mp = 207–210 °C (EtOH). ^1^H-NMR (400 MHz, DMSO-*d*_6_) δ 1.32 (t, 3H, *J* = 7.0 Hz, OCH_2_CH_3_); 3.81 (s, 9H, 3 × OCH_3_); 4.28 (q, 2H, *J* = 7.0 Hz, OCH_2_CH_3_); 4.46 (s, 4H, 2 × SO_2_N-CH_2_Ph); 5.85 (s, 2H, N-CH_2_Ph); 7.20 (d, 4H, *J* = 8.0 Hz, Ar); 7.42 (d, 2H, *J* = 8.0 Hz, Ar); 7.72 (d, 4H, *J* = 8.0 Hz, Ar); 7.99 (d, 3H, *J* = 8.4 Hz, Ar); 8.12 (d, 1H, *J* = 8.8 Hz, Ar); 8.47 (s, 1H, Ar); 9.02 (s, 1H, Ar). Anal. C_39_H_36_N_2_O_11_S (C, H, N) calcd. C, 63.23; H, 4.90; N, 3.78; found: C, 63.48; H, 4.92; N, 3.79.

*4,4′-{[((3-carboxy-1-(4-carboxybenzyl)-4-oxo-1,4-dihydroquinolin-6-yl)sulfonyl) azanediyl]bis(methylene)} dibenzoic acid* (**7**). A mixture of **6** (0.054 mmol), NaOH 40% (2 mL) and EtOH 96% (0.5 mL) was stirred at reflux for 2 h. After cooling, ice-cold water was added, the mixture was acidified with HCl 6M, and the precipitate was recovered by vacuum filtration and recrystallized with ethanol. Yield = 99%; mp > 300 °C (EtOH). ^1^H-NMR (400 MHz, DMSO-*d*_6_) δ 4.50 (s, 4H, 2 × SO_2_N-*CH_2_*Ph); 6.01 (s, 2H, N-*CH_2_*Ph); 7.22 (d, 4H, *J* = 7.6 Hz, Ar); 7.40 (d, 2H, *J* = 7.6 Hz, Ar); 7.70 (d, 4H, *J* = 7.6 Hz, Ar); 7.89 (d, 1H, *J* = 9.2 Hz, Ar); 7.97 (d, 2H, *J* = 8.0 Hz, Ar); 8.27 (d, 1H, *J* = 8.0 Hz, Ar); 8.42 (s, 1H, Ar); 9.36 (s, 1H, Ar). ^13^C-NMR (100 MHz, DMSO-*d*_6_) δ 26.2; 45.7; 52.9; 54.6; 54.9; 67.5; 97.6; 122.3; 125.0; 125.9; 126.3; 128.0; 129.2; 144.5; 149.5; 163.1. ESI-MS calcd. for C_34_H_26_N_2_O_11_S, 670.65; found: *m*/*z* 671.13 [M + H]^+^. Anal. C_34_H_26_N_2_O_11_S (C, H, N) calcd. C, 60.89; H, 3.91; N, 4.18; found: C, 60.65; H, 3.89; N, 4.16.

*General procedure for the synthesis of intermediates* **9a**–**c**. To a solution of 3.20 mmol of suitable amine of type **8** in 8 mL of 1,4-dioxane, 3.20 mmol of diethylethoxymethylen malonate was added, and the mixture was stirred at reflux for 4 h. After cooling at room temperature, the solvent was evaporated, and the solid formed was recovered with small amounts of ethanol and filtrated to obtain the desired compound (**9a** [23]).

*Diethyl 2-{[(4-(N,N-dipropylsulfamoyl)phenyl)amino]methylene}malonate* (**9b**). Yield = 40%; mp = 96–98 °C (EtOH). ^1^H-NMR (400 MHz, DMSO-*d*_6_) δ 0.80 (t, 6H, *J* = 7.2 Hz, 2 × SO_2_NCH_2_CH_2_*CH_3_*); 1.22 (t, 6H, *J* = 7.2 Hz, 2 × OCH_2_*CH_3_*); 1.45–1.50 (m, 4H, SO_2_NCH_2_*CH_2_*CH_3_); 2.99 (t, 4H, *J* = 8.8 Hz, 2 × SO_2_N*CH_2_*CH_2_CH_3_); 4.07–4.23 (m, 4H, 2 × O*CH_2_*CH_3_); 7.60 (d, 2H, *J* = 8.7 Hz, Ar); 7.74 (d, 2H, *J* = 8.7 Hz, Ar); 8.36 (d, 1H, *J* = 13.2 Hz, C=*CH*); 10.67 (exch br d, 1H, *J* = 13.2 Hz, NH). Anal. C_20_H_30_N_2_O_6_S (C, H, N) calcd. C, 56.32; H, 7.09; N, 6.57; found: C, 56.54; H, 7.11; N, 6.59.

*Diethyl 2-{[(4-(N,N-dibenzylsulfamoyl)phenyl)amino]methylene}malonate* (**9c**). Yield = 59%; mp = 117–118 °C (EtOH). ^1^H-NMR (400 MHz, CDCl_3_) δ 1.49–1.57 (m, 6H, 2 × OCH_2_*CH_3_*); 4.41–4.49 (m, 4H, 2 × O*CH_2_*CH_3_); 4.52 (s, 4H, 2 × N-*CH_2_*Ph); 7.20–7.25 (m, 4H, Ar); 7.35–7.42 (m, 8H, Ar); 7.97 (d, 2H, *J* = 8.8 Hz, Ar); 8.68 (d, 1H, *J* = 13.2 Hz, CH); 11.30 (exch br d, 1H, *J* = 13.2 Hz, NH). Anal. C_28_H_30_N_2_O_6_S (C, H, N) calcd. C, 64.35; H, 5.79; N, 5.36; found: C, 64.60; H, 5.81; N, 5.38.

*General procedure for the synthesis of intermediates* **10a**,**b**. Compounds **10a**,**b** were obtained following the same synthetic procedure and the same number of equivalents used for compound **3** but starting from intermediates **9a**,**b** (0.50 mmol) and modifying the temperature (100 °C) and the reaction time (3 h). The crude compound was purified by crystallization with ethanol (**10a** [23]).

*Ethyl 6-(N,N-dipropylsulfamoyl)-4-oxo-1,4-dihydroquinoline-3-carboxylate* (**10b**). Yield = 68%; mp > 300 °C (EtOH). ^1^H-NMR (400 MHz, DMSO-*d*_6_) δ 0.85–0.95 (m, 6H, 2 × SO_2_NCH_2_CH_2_*CH_3_*); 1.27 (t, 3H, *J* = 6.8 Hz, OCH_2_*CH_3_*); 1.43–1.51 (m, 4H, 2 × SO_2_NCH_2_*CH_2_*CH_3_); 3.02–3.10 (m, 4H, 2 × SO_2_N*CH_2_*CH_2_CH_3_); 4.24 (q, 2H, *J* = 7.0 Hz, O*CH_2_*CH_3_); 7.95 (d, 1H, *J* = 8.8 Hz, Ar); 8.06 (dd, 1H, *J*_1_ = 8.6 Hz, *J*_2_ = 2.2 Hz, Ar,); 8.47 (s, 1H, Ar); 8.65 (s, 1H, Ar); 12.65 (exch br s, 1H, NH). Anal. C_18_H_24_N_2_O_5_S (C, H, N) calcd. C, 56.83; H, 6.36; N, 7.36; found: C, 56.60; H, 6.33; N, 7.33.

*General procedure for the synthesis of compounds* **11a**–**g**. Compounds **11a**–**g** were obtained following the same synthetic procedure with the same number of equivalents used for compounds **4a**,**b** but starting from intermediates **10a**,**b** and using the appropriate reagent. The crude compounds were purified by flash column chromatography using dichloromethane/methanol 95:5 (for **11a**,**g**), ethyl acetate (for **11c**,**f**) or cyclohexane/ethyl acetate 1:1 (for **11b**,**d**,**e**) as the eluent.

*Ethyl 1-benzyl-6-(N,N-diethylsulfamoyl)-4-oxo-1,4-dihydroquinoline-3-carboxylate* (**11a**). Benzyl bromide (0.38 mmol) (commercially available) was used as the reagent. Yield = 44%; mp = 185–186 °C (EtOH). ^1^H-NMR (400 MHz, CDCl_3_) δ 1.14 (t, 6H, *J* = 7.2 Hz, 2 × SO_2_NCH_2_*CH_3_*); 1.44 (t, 3H, *J* = 7.0 Hz, OCH_2_*CH_3_*); 3.27 (q, 4H, *J* = 7.2 Hz, 2 × SO_2_N*CH_2_*CH_3_); 4.43 (q, 2H, *J* = 7.0 Hz, O*CH_2_*CH_3_); 5.44 (s, 2H, N-*CH_2_*Ph); 7.19 (d, 2H, *J* = 6.4 Hz, Ar); 7.39–7.45 (m, 4H, Ar); 7.96 (dd, 1H, *J*_1_ = 8.8 Hz, *J*_2_ = 2.0 Hz, Ar); 8.65 (s, 1H, Ar); 8.90 (s, 1H, Ar). Anal. C_23_H_26_N_2_O_5_S (C, H, N) calcd. C, 62.43; H, 5.92; N, 6.33; found: C, 62.68; H, 5.94; N, 6.35.

*Ethyl-6-(N,N-diethylsulfamoyl)-1-[4-(methoxycarbonyl)benzyl]-4-oxo-1,4-dihydroquinoline-3-carboxylate* (**11b**). Methyl 4-(bromomethyl)benzoate (0.42 mmol) [22] was used as the reagent. Yield = 29%; mp = 210–212 °C (EtOH). ^1^H-NMR (400 MHz, CDCl_3_) δ 1.13 (t, 6H, *J* = 7.2 Hz, 2 × SO_2_NCH_2_*CH_3_*); 1.43 (t, 3H, *J* = 7.2 Hz, OCH_2_*CH_3_*); 3.24 (q, 4H, *J* = 7.2 Hz, 2 × SO_2_N*CH_2_*CH_3_); 3.92 (s, 3H, O*CH_3_*); 4.42 (q, 2H, *J* = 7.2 Hz, O*CH_2_*CH_3_); 5.50 (s, 2H, N-*CH_2_*Ph); 7.24–7.32 (m, 3H, Ar); 7.88 (dd, 1H, *J*_1_ = 8.0 Hz, *J*_2_ = 2.2 Hz, Ar); 8.05 (d, 2H, *J* = 8.4 Hz, Ar); 8.63 (s, 1H, Ar); 8.86 (s, 1H, Ar). Anal. C_25_H_28_N_2_O_7_S (C, H, N) calcd. C, 59.99; H, 5.64; N, 5.60; found: C, 59.75; H, 5.62; N, 5.58.

*Ethyl-6-(N,N-diethylsulfamoyl)-4-oxo-1-(4-sulfamoylbenzyl)-1,4-dihydroquinoline-3-carboxylate* (**11c**). 4-(bromomethyl)benzenesulphonamide (0.85 mmol) [33] was used as the reagent. Yield = 20%; mp = 218–220 °C (EtOH). ^1^H-NMR (400 MHz, DMSO-*d*_6_) δ 1.04 (t, 6H, *J* = 7.2 Hz, 2 × SO_2_NCH_2_*CH_3_*); 1.31 (t, 3H, *J* = 7.2 Hz, OCH_2_*CH_3_*); 3.15–3.20 (m, 4H, 2 × SO_2_N*CH_2_*CH_3_); 4.40 (q, 2H, *J* = 7.2 Hz, O*CH_2_*CH_3_); 5.82 (s, 2H, N-*CH_2_*Ph); 7.36 (exch br s, 2H, SO_2_NH_2_); 7.44 (d, 2H, *J* = 8.0 Hz, Ar); 7.73 (d, 1H, *J* = 9.2 Hz, Ar); 7.80 (d, 2H, *J* = 8.0 Hz, Ar); 8.02 (d, 1H, *J* = 8.4 Hz, Ar); 8.57 (s, 1H, Ar); 9.00 (s, 1H, Ar). Anal. C_23_H_27_N_3_O_7_S_2_ (C, H, N) calcd. C, 52.96; H, 5.22; N, 8.06; found: C, 52.75; H, 5.20; N, 8.03.

*Ethyl 1-benzyl-6-(N,N-dipropylsulfamoyl)-4-oxo-1,4-dihydroquinoline-3-carboxylate* (**11d**). Benzyl bromide (0.55 mmol) (commercially available) was used as the reagent. Yield = 23%; mp = 155–158 °C (EtOH). ^1^H-NMR (400 MHz, CDCl_3_) δ 0.85 (t, 6H, *J* = 7.2 Hz, 2 × SO_2_NCH_2_CH_2_*CH_3_*); 1.43 (t, 3H, *J* = 6.8 Hz, OCH_2_*CH_3_*); 1.53–1.58 (m, 4H, 2 × SO_2_NCH_2_*CH_2_*CH_3_); 3.09 (t, 4H, *J* = 7.4 Hz, 2 × SO_2_N*CH_2_*CH_2_CH_3_); 4.41 (q, 2H, *J* = 6.8 Hz, O*CH_2_*CH_3_); 5.44 (s, 2H, N-*CH_2_*Ph); 7.18 (d, 2H, *J* = 6.8 Hz, Ar); 7.38–7.43 (m, 4H, Ar); 7.91 (d, 1H, *J* = 8.8 Hz, Ar); 8.63 (s, 1H, Ar); 8.88 (s, 1H, Ar). Anal. C_25_H_30_N_2_O_5_S (C, H, N) calcd. C, 63.81; H, 6.43; N, 5.95; found: C, 63.55; H, 6.40; N, 5.92.

*Ethyl-6-(N,N-dipropylsulfamoyl)-1-[4-(methoxycarbonyl)benzyl]-4-oxo-1,4-dihydroquinoline-3-carboxylate* (**11e**). Methyl 4-(bromomethyl)benzoate (0.51 mmol) [22] was used as the reagent. Yield = 39%; mp = 163–165 °C (EtOH). ^1^H-NMR (400 MHz, CDCl_3_) δ 0.84 (t, 6H, *J* = 7.0 Hz, 2 × SO_2_NCH_2_CH_2_*CH_3_*); 1.41 (t, 3H, *J* = 6.8 Hz, OCH_2_*CH_3_*); 1.51–1.56 (m, 4H, 2 × SO_2_NCH_2_*CH_2_*CH_3_); 3.06 (t, 4H, *J* = 7.2 Hz, 2 × SO_2_N*CH_2_*CH_2_CH_3_); 3.92 (s, 3H, O*CH_3_*); 4.40 (q, 2H, *J* = 6.8 Hz, O*CH_2_*CH_3_); 5.51 (s, 2H, N-*CH_2_*Ph); 7.24 (d, 2H, *J* = 8.0 Hz, Ar); 7.31 (d, 1H, *J* = 8.8 Hz, Ar); 7.86 (d, 1H, *J* = 8.8 Hz, Ar); 8.04 (d, 2H, *J* = 8.0 Hz, Ar); 8.65 (s, 1H, Ar); 8.80 (s, 1H, Ar). Anal. C_27_H_32_N_2_O_7_S (C, H, N) calcd. C, 61.35; H, 6.10; N, 5.30; found: C, 61.59; H, 6.12; N, 5.32.

*Ethyl-6-(N,N-dipropylsulfamoyl)-4-oxo-1-(4-sulfamoylbenzyl)-1,4-dihydroquinoline-3-carboxylate* (**11f**). 4-(bromomethyl)benzenesulphonamide (0.80 mmol) [33] was used as the reagent. Yield = 30%; mp > 300 °C (EtOH). ^1^H-NMR (400 MHz, DMSO-*d*_6_) δ 0.79 (t, 6H, *J* = 7.2 Hz, 2 × SO_2_NCH_2_CH_2_*CH_3_*); 1.31 (t, 3H, *J* = 7.0 Hz, OCH_2_*CH_3_*); 1.40–1.47 (m, 4H, 2 × SO_2_NCH_2_*CH_2_*CH_3_); 3.02 (t, 4H, *J* = 6.8 Hz, 2 × SO_2_N*CH_2_*CH_2_CH_3_); 4.28 (q, 2H, *J* = 7.2 Hz, O*CH_2_*CH_3_); 5.82 (s, 2H, N-*CH_2_*Ph); 7.36 (exch br s, 2H, SO_2_NH_2_); 7.44 (d, 2H, *J* = 8.0 Hz, Ar); 7.73 (d, 1H, *J* = 8.8 Hz, Ar); 7.80 (d, 2H, *J* = 8.0 Hz, Ar); 8.02 (d, 1H, *J* = 6.4 Hz, Ar); 8.56 (s, 1H, Ar); 9.01 (s, 1H, Ar). Anal. C_25_H_31_N_3_O_7_S_2_ (C, H, N) calcd. C, 54.63; H, 5.68; N, 7.64; found: C, 54.85; H, 5.70; N, 7.67.

*Ethyl 6-(N,N-dipropylsulfamoyl)-4-oxo-1-phenethyl-1,4-dihydroquinoline-3-carboxylate* (**11g**). Phenethyl bromide (0.36 mmol) (commercially available) was used as the reagent. Yield = 17%; mp = 185–188 °C (EtOH). ^1^H-NMR (400 MHz, CDCl_3_) δ 0.88 (t, 6H, *J* = 7.4 Hz, 2 × SO_2_NCH_2_CH_2_*CH_3_*); 1.38 (t, 3H, *J* = 7.0 Hz, OCH_2_*CH_3_*); 1.55–1.61 (m, 4H, 2 × SO_2_NCH_2_*CH_2_*CH_3_); 3.12–3.19 (m, 6H, 2 × SO_2_N*CH_2_*CH_2_CH_3_ + N-CH_2_-*CH_2_*-Ph); 4.35 (q, 2H, *J* = 7.0 Hz, O*CH_2_*CH_3_); 4.43 (t, 2H, *J* = 7.0 Hz, N-*CH_2_*CH_2_-Ph); 7.06 (d, 2H, *J* = 7.6 Hz, Ar); 7.25–7.30 (m, 3H, Ar); 7.55 (d, 1H, *J* = 8.8 Hz, Ar); 8.09 (dd, 1H, *J*_1_ = 8.8 Hz, *J*_2_ = 2.4 Hz, Ar); 8.17 (s, 1H, Ar); 8.91 (s, 1H, Ar). Anal. C_26_H_32_N_2_O_5_S (C, H, N) calcd. C, 64.44; H, 6.66; N, 5.78; found: C, 64.70; H, 6.68; N, 5.80.

*General procedure for the synthesis of acid compounds* **12a**–**g**. Compounds **12a**–**g** were obtained following the same synthetic procedure and the same number of equivalents used for compounds **5a**,**b**, but starting from ester derivatives **11a**–**g**. The compounds were purified by crystallization with ethanol.

*1-Benzyl-6-(N,N-diethylsulfamoyl)-4-oxo-1,4-dihydroquinoline-3-carboxylic acid* (**12a**). Yield = 88%; mp > 300 °C (EtOH). ^1^H-NMR (400 MHz, DMSO-*d*_6_) δ 1.03 (t, 6H, *J* = 7.0 Hz, 2 × SO_2_NCH_2_*CH_3_*); 3.16 (q, 4H, *J* = 7.0 Hz, 2 × SO_2_N*CH_2_*CH_3_); 5.74 (s, 2H, N-*CH_2_*Ph); 7.24–7.38 (m, 5H, Ar); 7.89 (d, 1H, *J* = 8.8 Hz, Ar); 8.02 (dd, 1H, *J*_1_ = 7.6 Hz, *J*_2_ = 1.8 Hz, Ar); 8.66 (s, 1H, Ar); 8.99 (s, 1H, Ar). ^13^C-NMR (100 MHz, DMSO-*d*_6_) δ 14.6; 42.4; 56.1; 119.8; 126.1; 127.0; 127.5; 128.4; 129.4; 130.1; 135.8; 136.2; 141.9; 150.5; 166.4; 176.5. ESI-MS calcd. for C_21_H_22_N_2_O_5_S, 414.48; found: *m*/*z* 415.13 [M + H]^+^. Anal. C_21_H_22_N_2_O_5_S (C, H, N) calcd. C, 60.86; H, 5.35; N, 6.76; found: C, 60.65; H, 5.37; N, 6.79.

*1-(4-Carboxybenzyl)-6-(N,N-diethylsulfamoyl)-4-oxo-1,4-dihydroquinoline-3-carboxylic acid* (**12b**). Yield = 54%; mp > 300 °C (EtOH). ^1^H-NMR (400 MHz, DMSO-*d*_6_) δ 1.04 (t, 6H, *J* = 7.0 Hz, 2 × SO_2_NCH_2_*CH_3_*); 3.19 (q, 4H, *J* = 7.0 Hz, 2 × SO_2_N*CH_2_*CH_3_); 6.00 (s, 2H, N-*CH_2_*Ph); 7.40 (d, 2H, *J* = 8.4 Hz, Ar); 7.91–7.96 (m, 3H, Ar); 8.17 (dd, 1H, *J*_1_ = 8.0 Hz, *J*_2_ = 2.2 Hz Ar); 8.67 (s, 1H, Ar); 9.37 (s, 1H, Ar); 12.98 (exch br s, 1H, COOH); 14.57 (exch br s, 1H, COOH). ^13^C-NMR (100 MHz, DMSO-*d*_6_) δ 14.6; 42.5; 56.9; 109.9; 120.9; 125.3; 126.2; 127.2; 130.4; 131.0; 131.6; 137.8; 140.4; 142.0; 152.2; 165.8; 167.3; 178.1. ESI-MS calcd. for C_22_H_22_N_2_O_7_S, 458.49; found: *m*/*z* 459.12 [M + H]^+^. Anal. C_22_H_22_N_2_O_7_S (C, H, N) calcd. C, 57.63; H, 4.84; N, 6.11; found: C, 57.40; H, 4.82; N, 6.08.

*6-(N,N-Diethylsulfamoyl)-4-oxo-1-(4-sulfamoylbenzyl)-1,4-dihydroquinoline-3-carboxylic acid* (**12c**). Yield = 80%; mp > 300 °C (EtOH). ^1^H-NMR (400 MHz, DMSO-*d*_6_) δ 1.05 (t, 6H, *J* = 7.0 Hz, 2 × SO_2_NCH_2_*CH_3_*); 3.20 (q, 4H, *J* = 7.2 Hz, 2 × SO_2_N*CH_2_*CH_3_); 5.99 (s, 2H, N-*CH_2_*Ph); 7.36 (exch br s, 2H, SO_2_NH_2_); 7.47 (d, 2H, *J* = 8.0 Hz, Ar); 7.80 (d, 2H, *J* = 8.0 Hz, Ar); 7.93 (d, 1H, *J* = 8.8 Hz, Ar); 8.16 (dd, 1H, *J*_1_ = 8.8 Hz, *J*_2_ = 2.2 Hz, Ar); 8.67 (s, 1H, Ar); 9.35 (s, 1H, Ar). ^13^C-NMR (100 MHz, DMSO-*d*_6_) δ 14.6; 42.5; 56.6; 110.5; 120.8; 125.4; 126.3; 126.7; 127.5; 131.5; 137.7; 139.5; 141.9; 144.2; 152.1; 165.8; 178.0. ESI-MS calcd. for C_22_H_22_N_2_O_7_S, 493.55; found: *m*/*z* 494.10 [M + H]^+^. Anal. C_21_H_23_N_3_O_7_S_2_ (C, H, N) calcd. C, 51.11; H, 4.70; N, 8.51; found: C, 51.31; H, 4.72; N, 8.54.

*1-Benzyl-6-(N,N-dipropylsulfamoyl)-4-oxo-1,4-dihydroquinoline-3-carboxylic acid* (**12d**). Yield = 56%; mp > 300 °C (EtOH). ^1^H-NMR (400 MHz, DMSO-*d*_6_) δ 0.78 (t, 6H, *J* = 7.2 Hz, 2 × SO_2_NCH_2_CH_2_*CH_3_*); 1.46 (sex, 4H, *J* = 7.2 Hz, 2 × SO_2_NCH_2_*CH_2_*CH_3_); 3.04 (t, 4H, *J* = 7.2 Hz, 2 × SO_2_N*CH_2_*CH_2_CH_3_); 5.82 (s, 2H, N-*CH_2_*Ph); 7.26–7.39 (m, 5H, Ar); 7.95 (d, 1H, *J* = 8.0 Hz, Ar); 8.10 (dd, 1H, *J*_1_ = 9.2 Hz, *J*_2_ = 2.0 Hz, Ar); 8.65 (s, 1H, Ar); 9.14 (s, 1H, Ar). ^13^C-NMR (100 MHz, DMSO-*d*_6_) δ 11.4; 22.1; 50.1; 54.9; 119.8; 120.3; 126.1; 125.7; 127.0; 127.1; 128.5; 129.4; 135.9; 141.9; 150.4; 166.0; 176.5. ESI-MS calcd. for C_23_H_26_N_2_O_5_S, 442.53; found: *m*/*z* 443.16 [M + H]^+^. Anal. C_23_H_26_N_2_O_5_S (C, H, N) calcd. C, 62.43; H, 5.92; N, 6.33; found: C, 62.68; H, 5.94; N, 6.35.

*1-(4-Carboxybenzyl)-6-(N,N-dipropylsulfamoyl)-4-oxo-1,4-dihydroquinoline-3-carboxylic acid* (**12e**). Yield = 46%; mp > 300 °C (EtOH). ^1^H-NMR (400 MHz, DMSO-*d*_6_) δ 0.78 (t, 6H, *J* = 7.4 Hz, 2 × SO_2_NCH_2_CH_2_*CH_3_*); 1.43–1.49 (m, 4H, 2 × SO_2_NCH_2_*CH_2_*CH_3_); 3.05 (t, 4H, *J* = 7.4 Hz, 2 × SO_2_N*CH_2_*CH_2_CH_3_); 6.00 (s, 2H, N-*CH_2_*Ph); 7.39 (d, 2H, *J* = 8.4 Hz, Ar); 7.90–7.95 (m, 3H, Ar); 8.19 (dd, 1H, *J*_1_ = 8.0 Hz, *J*_2_ = 2.4 Hz, Ar); 8.66 (s, 1H, Ar); 9.37 (s, 1H, Ar); 12.87 (exch br s, 1H, COOH); 14.55 (exch br s, 1H, COOH). ^13^C-NMR (100 MHz, DMSO-*d*_6_) δ 11.4; 22.1; 50.1; 56.9; 109.9; 120.9; 125.3; 126.2; 126.9; 127.2; 129.6; 130.9; 131.7; 137.6; 140.5; 142.0; 152.2; 165.7; 167.3; 178.1. ESI-MS calcd. for C_24_H_26_N_2_O_7_S, 486.54; found: *m*/*z* 487.15 [M + H]^+^. Anal. C_24_H_26_N_2_O_7_S (C, H, N) calcd. C, 59.25; H, 5.39; N, 5.76; found: C, 59.49; H, 5.41; N, 5.78.

*6-(N,N-Dipropylsulfamoyl)-4-oxo-1-(4-sulfamoylbenzyl)-1,4-dihydroquinoline-3-carboxylic acid* (**12f**). Yield = 99%; mp > 300 °C (EtOH). ^1^H-NMR (400 MHz, DMSO-*d*_6_) δ 0.79 (t, 6H, *J* = 7.2 Hz, 2 × SO_2_NCH_2_CH_2_*CH_3_*); 1.47 (sex, 4H, *J* = 7.2 Hz, 2 × SO_2_NCH_2_*CH_2_*CH_3_); 3.06 (t, 4H, *J* = 7.2 Hz, 2 × SO_2_N*CH_2_*CH_2_CH_3_); 6.00 (s, 2H, N-*CH_2_*Ph); 7.37 (exch br s, 2H, SO_2_NH_2_); 7.47 (d, 2H, *J* = 8.0 Hz, Ar); 7.80 (d, 2H, *J* = 8.0 Hz, Ar); 7.94 (d, 1H, *J* = 9.2 Hz, Ar); 8.18 (d, 1H, *J* = 8.4 Hz, Ar); 8.67 (s, 1H, Ar); 9.38 (s, 1H, Ar); 14.55 (exch br s, 1H, COOH). ^13^C-NMR (100 MHz, DMSO-*d*_6_) δ 11.4; 22.1; 50.2; 56.7; 109.9; 120.8; 125.4; 126.0; 126.2; 127.5; 127.8; 128.3; 131.7; 137.6; 139.4; 141.9; 144.2; 152.3; 165.8; 178.2. ESI-MS calcd. for C_23_H_27_N_3_O_7_S_2_, 521.60; found: *m*/*z* 522.13 [M + H]^+^. Anal. C_23_H_27_N_3_O_7_S_2_ (C, H, N) calcd. C, 52.96; H, 5.22; N, 8.06; found: C, 52.75; H, 5.20; N, 8.03.

*6-(N,N-Dipropylsulfamoyl)-4-oxo-1-phenethyl-1,4-dihydroquinoline-3-carboxylic acid* (**12g**). Yield = 75%; mp = 220–222 °C (EtOH). ^1^H-NMR (400 MHz, DMSO-*d*_6_) δ 0.82 (t, 6H, *J* = 6.4 Hz, 2 × SO_2_NCH_2_CH_2_*CH_3_*); 1.47–1.53 (m, 4H, 2 × SO_2_NCH_2_*CH_2_*CH_3_); 3.10–3.15 (m, 6H, 2 × SO_2_N*CH_2_*CH_2_CH_3_ + N-CH_2_-*CH_2_*-Ph); 4.84 (s, 2H, N-*CH_2_*CH_2_-Ph); 7.22–7.27 (m, 5H, Ar); 8.23–8.28 (m, 2H, Ar); 8.65 (s, 1H, Ar); 8.94 (s, 1H, Ar). ^13^C-NMR (100 MHz, DMSO-*d*_6_) δ 11.4; 22.4; 50.2; 55.7; 109.9; 120.7; 125.4; 126.1; 126.2; 127.7; 127.8; 128.5; 131.7; 137.4; 139.4; 141.9; 144.2; 152.4; 165.8; 178.1. ESI-MS calcd. for C_24_H_28_N_2_O_5_S, 456.56; found: *m*/*z* 457.18 [M + H]^+^. Anal. C_24_H_28_N_2_O_5_S (C, H, N) calcd. C, 63.14; H, 6.18; N, 6.14; found: C, 63.39; H, 6.20; N, 6.16.

*General procedure for the synthesis of intermediates* **15** *and* **16**. To a solution of 1.50 mmol of 2,8-bis(trifluoromethyl)quinolin-4-ol **13** [26] (for **15**) or 8-(trifluoromethyl) quinolin-4-ol **14** [27] (for **16**) in dry DMF (3–4 mL), 3.0 mmol of anhydrous K_2_CO_3_ was added, and the mixture was stirred for 30 min at room temperature. Then, 4.50 mmol of 1,3-dibromopropane was added, and the reaction was heated at 80–90 °C for 3–4 h. After evaporation of the solvent, ice/water was added, and the precipitate formed was filtered under a *vacuum*, washed with water, and purified by flash column chromatography using hexane/ethyl acetate 2:1 (for **15**) and 4:1 (for **16**) as eluents.

*4-(3-Bromopropoxy)-2,8-bis(trifluoromethyl)quinoline* (**15**). Yield = 49%; mp = 97–100 °C (EtOH). ^1^H-NMR (400 MHz, CDCl_3_) δ 2.56 (quin, 2H, *J* = 6.0 Hz, O-CH_2_-*CH_2_*-CH_2_-Br); 3.71 (t, 2H, *J* = 6.4 Hz, CH_2_-Br); 4.48 (t, 2H, *J* = 5.8 Hz, O-CH_2_); 7.18 (s, 1H, Ar); 7.68 (t, 1H, *J* = 7.8 Hz, Ar); 8.16 (d, 1H, *J* = 7.2 Hz, Ar); 8.45 (d, 1H, *J* = 8.4 Hz, Ar). Anal. C_14_H_10_BrF_6_NO (C, H, N) calcd. C, 41.82; H, 2.51; N, 4.48; found: C, 41.65; H, 2.50; N, 3.47.

*4-(3-Bromopropoxy)-8-(trifluoromethyl)quinoline* (**16**). Yield = 30%; mp = 70–72 °C (EtOH). ^1^H-NMR (400 MHz, CDCl_3_) δ 2.51 (quin, 2H, *J* = 6.2 Hz, O-CH_2_-*CH_2_-*CH_2_-Br); 3.68 (t, 2H, *J* = 6.4 Hz, CH_2_-Br); 4.38 (t, 2H, *J* = 6.0 Hz, O-CH_2_); 6.86 (d, 1H, *J* = 5.2 Hz, Ar); 7.56 (t, 1H, *J* = 7.6 Hz, Ar); 8.07 (d, 1H, *J* = 7.2 Hz, Ar); 8.41 (d, 1H, *J* = 8.4 Hz, Ar); 8.91 (d, 1H, *J* = 5.2 Hz, Ar). Anal. C_13_H_11_BrF_3_NO (C, H, N) calcd. C, 46.73; H, 3.32; N, 4.19; found: C, 46.91; H, 3.33; N, 4.21.

*General procedure for the synthesis of compounds* **17a**–**f** *and* **18b**–**f**. To a solution of 0.20 mmol of suitable reagents (amine or alcohol) in dry DMF (2.5 mL), 0.40 mmol of anhydrous K_2_CO_3_ was added, and the mixture was stirred for 30 min at room temperature. Then, 0.30 mmol of suitable intermediate **15** (for **17a**–**f**) or **16** (for **18b**–**f**) was added, and the reaction mixture was stirred at room temperature for 24–72 h. After evaporation of the solvent, ice/water was added and the suspension obtained was extracted with ethyl acetate (3 × 15 mL), dried on sodium sulphate and evaporated. For compound **17a**, we obtained a precipitate, which was filtered under a *vacuum* and washed with water. The crude products were purified by flash column chromatography using cyclohexane/ethyl acetate 2:1 (for **17f**), dichloromethane/methanol 80:20 (for **17b**), 90:10 (for **17c**,**d** and **18d**), 95:5 (for **18b**,**c**,**e**,**f**), 99:1 (for **17e**), dichloromethane/methanol/ammonia 80:20:2 (for **17a**) or dichloromethane/methanol 90:10 (for **21d**) as eluents.

*(1S,4S)-3-{3-[(2,8-bis(Trifluoromethyl)quinolin-4-yl)oxy]propoxy}quinuclidine* (**17a**). Quinuclidinol (commercially available) was used as the reagent. Reaction time: 48 h. Yield = 60%; mp = 226–229 °C (EtOH). ^1^H-NMR (400 MHz, DMSO-*d*_6_) δ 1.70–1.85 (m, 2H, CH_2_ quin.); 1.90–200 (m, 1H, CH-*H* quin.); 2.05–2.10 (m, 1H, CH-*H* quin.); 2.15–2.25 (m, 1H, CH-*H* quin.); 2.30–2.40 (m, 2H, O-CH_2_-*CH_2_*-CH_2_-O); 3.14 (d, 1H, *J* = 12.8 Hz, CH*-H* quin.); 3.35–3.50 (m, 5H, *CH_2_*-O-quin. + CH_2_ CH-*H* quin.); 3.74 (t, 1H, *J* = 9.8 Hz, CH-*H* quin.); 4.05–4.11 (m, 1H, CH-*H* quin.); 4.52 (t, 2H, *J* = 6.0 Hz, O-CH_2_); 5.69 (d, 1H, *J* = 3.2 Hz, CH-H quin.); 7.58 (s, 1H, Ar); 7.87 (t, 1H, *J* = 7.8 Hz, Ar); 8.34 (d, 1H, *J* = 7.2 Hz, Ar); 8.65 (d, 1H, *J* = 8.4 Hz, Ar). ^13^C-NMR (100 MHz, DMSO-*d*_6_) δ 17.9; 21.5; 21.9; 26.8; 53.2; 54.5; 60.7; 63.1; 63.9; 67.4; 99.5; 116.4; 122.2; 124.4; 127.5; 127.9; 128.1; 130.6; 141.8; 156.7; 162.1. ESI-MS calcd. for C_21_H_22_F_6_N_2_O_2_, 448.41; found: *m*/*z* 449.16 [M + H]^+^. Anal. C_21_H_22_F_6_N_2_O_2_ (C, H, N) calcd. C, 54.44; H, 5.36; N, 8.28; found: C, 54.66; H, 5.38; N, 8.31.

*4-[3-(4-Methylpiperazin-1-yl)propoxy]-2,8-bis(trifluoromethyl)quinoline* (**17b**). Methylpiperazine (commercially available) was used as the reagent. Reaction time: 24 h. Yield = 29%; mp = 73–75 °C (EtOH). ^1^H-NMR (400 MHz, CDCl_3_) δ 2.17 (quin, 2H, *J* = 6.6 Hz, O-CH_2_-*CH_2_*-CH_2_-N); 2.32 (s, 3H, CH_3_); 2.45–2.60 (m, 8H, 4 × CH_2_ piperazine); 2.62 (t, 2H, *J* = 7.2 Hz, CH_2_-N); 4.36 (t, 2H, *J* = 6.2 Hz, O-CH_2_); 7.14 (s, 1H, Ar); 7.64 (t, 1H, *J* = 8.0 Hz, Ar); 8.12 (d, 1H, *J* = 7.2 Hz, Ar); 8.44 (d, 1H, *J* = 8.4 Hz, Ar). ^13^C-NMR (100 MHz, CDCl_3_) δ 26.2; 45.7; 52.9; 54.6; 54.9; 67.5; 97.6; 122.3; 125.0; 125.9; 126.3; 128.0; 129.2; 144.5; 149.5; 163.1. ESI-MS calcd. for C_19_H_21_F_6_N_3_O, 421.39; found: *m*/*z* 422.16 [M + H]^+^. Anal. C_19_H_21_F_6_N_3_O (C, H, N) calcd. C, 54.16; H, 5.02; N, 9.97; found: C, 54.38; H, 5.04; N, 10.00.

*4-[3-(Piperidin-1-yl)propoxy]-2,8-bis(trifluoromethyl)quinoline* (**17c**). Piperidine (commercially available) was used as the reagent. Reaction time: 24 h. Yield = 74%; mp = 60–63 °C (EtOH). ^1^H-NMR (400 MHz, CDCl_3_) δ 1.45–1.50 (m, 2H, CH_2_ piperidine); 1.65 (quin, 4H, *J* = 5.2 Hz, 2 × CH_2_ piperidine); 2.21 (quin, 2H, *J* = 6.8 Hz, O-CH_2_-*CH_2_-*CH_2_-N); 2.45–2.55 (m, 4H, 2 × CH_2_ piperidine); 2.63 (t, 2H, *J* = 7.4 Hz, CH_2_-N); 4.34 (t, 2H, *J* = 6.2 Hz, O-CH_2_); 7.12 (s, 1H, Ar); 7.62 (t, 1H, *J* = 7.8 Hz, Ar); 8.10 (d, 1H, *J* = 7.2 Hz, Ar); 8.43 (d, 1H, *J* = 8.4 Hz, Ar). ^13^C-NMR (100 MHz, CDCl_3_) δ 24.0; 25.5; 25.9; 54.4; 55.3; 67.7; 97.6; 122.3; 125.0; 125.9; 126.4; 128.0; 129.2; 144.5; 149.5; 149.8; 163.1. ESI-MS calcd. for C_19_H_20_F_6_N_2_O, 406.37; found: *m*/*z* 407.15 [M + H]^+^. Anal. C_19_H_20_F_6_N_2_O (C, H, N) calcd. C, 56.16; H, 4.96; N, 6.89; found: C, 56.38; H, 4.98; N, 6.91 [28].

*3-{[2,8-bis(Trifluoromethyl)quinolin-4-yl]oxy}-N,N-diethylpropan-1-amine* (**17d**). Diethylamine (commercially available) was used as the reagent. Reaction time: 72 h. Yield = 10%; oil. ^1^H-NMR (400 MHz, CDCl_3_) δ 1.16 (t, 6H, *J* = 7.2 Hz, 2 × CH_3_); 2.27 (t, 2H, *J* = 6.0 Hz, O-CH_2_-*CH_2_*-CH_2_-N); 2.75 (q, 4H, *J* = 7.2 Hz, 2 × N-*CH_2_*CH_3_); 2.87 (t, 2H, *J* = 6.8 Hz, CH_2_-N); 4.38 (t, 2H, *J* = 6.0 Hz, O-CH_2_); 7.13 (s, 1H, Ar); 7.65 (t, 1H, *J* = 7.8 Hz, Ar); 8.13 (d, 1H, *J* = 7.2 Hz, Ar); 8.43 (d, 1H, *J* = 8.4 Hz, Ar). ^13^C-NMR (100 MHz, CDCl_3_) δ 10.9; 26.0; 46.9; 49.0; 67.4; 97.6; 119.6; 122.5; 125.0; 126.0; 126.2; 128.4; 129.3; 144.5; 149.5; 162.9. ESI-MS calcd. for C_18_H_20_F_6_N_2_O, 394.36; found: *m*/*z* 395.15 [M + H]^+^. Anal. C_18_H_20_F_6_N_2_O (C, H, N) calcd. C, 54.82; H, 5.11; N, 7.10; found: C, 54.60; H, 5.09; N, 7.07.

*4-{3-[(2,8-Bis(trifluoromethyl)quinolin-4-yl)oxy]propyl}morpholine* (**17e**). Morpholine (commercially available) was used as the reagent. Reaction time: 72 h. Yield = 24%; mp = 84–87 °C (EtOH). ^1^H-NMR (400 MHz, CDCl_3_) δ 2.15–2.25 (m, 2H, O-CH_2_-*CH_2_-*CH_2_-N); 2.50–2.60 (m, 3H, CH_2_ + CH-*H* morpholine); 2.65–2.70 (m, 2H, CH_2_-N); 3.35–3.40 (m, 2H, CH_2_ morpholine); 3.75–3.85 (m, 3H, CH_2_ + CH-*H* morpholine); 4.38 (t, 2H, *J* = 5.8 Hz, O-CH_2_); 7.15 (s, 1H, Ar); 7.65 (t, 1H, *J* = 7.6 Hz, Ar); 8.13 (d, 1H, *J* = 6.8 Hz, Ar); 8.44 (d, 1H, *J* = 8.4 Hz, Ar). ^13^C-NMR (100 MHz, CDCl_3_) δ 25.8; 53.7; 55.1; 66.7; 67.4; 97.6; 116.4; 120.4; 124.4; 126.0; 126.2; 128.1; 129.2; 145.8; 150.6; 162.1. ESI-MS calcd. for C_18_H_18_F_6_N_2_O_2_, 408.34; found: *m*/*z* 409.13 [M + H]^+^. Anal. C_18_H_18_F_6_N_2_O_2_ (C, H, N) calcd. C, 52.95; H, 4.44; N, 6.86; found: C, 52.74; H, 4.42; N, 6.83 [28].

*Tert-butyl-4-{3-[(2,8-bis(trifluoromethyl)quinolin-4-yl)oxy]propyl}piperazine-1-carboxylate* (**17f**). Tert-butylpiperazine-1-carboxylate (commercially available) was used as the reagent. Reaction time: 72 h. Yield = 29%; oil. ^1^H-NMR (400 MHz, CDCl_3_) δ 1.47 (s, 9H, C(CH_3_)_3_); 2.20–2.25 (m, 2H, O-CH_2_-*CH*_2_*-*CH_2_-Br); 2.48–2.55 (m, 4H, 2 × CH_2_ piperazine); 2.65–2.70 (m, 2H, CH_2_ piperazine); 3.45–3.55 (m, 4H, CH_2_-N-CH_2_ piperazine); 4.37 (t, 2H, *J* = 6.2 Hz, O-CH_2_); 7.14 (s, 1H, Ar); 7.65 (t, 1H, *J* = 8.0 Hz, Ar); 8.13 (d, 1H, *J* = 7.2 Hz, Ar); 8.43 (d, 1H, *J* = 8.4 Hz, Ar). ESI-MS calcd. for C_23_H_27_F_6_N_3_O_3_, 507.48; found: *m*/*z* 508.20 [M + H]^+^. Anal. C_23_H_27_F_6_N_3_O_3_ (C, H, N) calcd. C, 56.25; H, 4.95; N, 6.25; found: C, 56.47; H, 4.97; N, 6.27.

*4-[3-(4-Methylpiperazin-1-yl)propoxy]-8-(trifluoromethyl)quinoline* (**18b**). Methylpiperazine (commercially available) was used as the reagent. Reaction time: 24 h. Yield = 57%; oil. ^1^H-NMR (400 MHz, CDCl_3_) δ 2.12 (quin, 2H, *J* = 6.8 Hz, O-CH_2_-*CH_2_*-CH_2_-N); 2.30 (s, 3H, CH_3_); 2.40–2.55 (m, 8H, 4 × CH_2_ piperazine); 2.60 (t, 2H, *J* = 7.2 Hz, CH_2_-N); 4.25 (t, 2H, *J* = 6.2 Hz, O-CH_2_); 6.81 (d, 1H, *J* = 5.2 Hz, Ar); 7.51 (t, 1H, *J* = 7.8 Hz, Ar); 8.03 (d, 1H, *J* = 7.2 Hz, Ar); 8.39 (d, 1H, *J* = 8.4 Hz, Ar); 8.86 (d, 1H, *J* = 5.2 Hz, Ar). ^13^C-NMR (100 MHz, CDCl_3_) δ 26.3; 45.9; 53.1; 54.8; 55.0; 66.9; 101.5; 122.1; 122.8; 123.9; 125.6; 126.5; 128.1; 145.7; 152.3; 161.5. ESI-MS calcd. for C_18_H_22_F_3_N_3_O, 353.39; found: *m*/*z* 354.17 [M + H]^+^. Anal. C_18_H_22_F_3_N_3_O (C, H, N) calcd. C, 60.13; H, 6.42; N, 9.56; found: C, 60.37; H, 6.44; N, 9.59.

*4-[3-(Piperidin-1-yl)propoxy]-8-(trifluoromethyl)quinoline* (**18c**). Piperidine (commercially available) was used as the reagent. Reaction time: 24 h. Yield = 49%; oil. ^1^H-NMR (400 MHz, CDCl_3_) δ 1.45–1.55 (m, 2H, CH_2_ piperidine); 1.71 (quin, 4H, *J* = 5.6 Hz, 2 × CH_2_ piperidine); 2.24 (quin, 2H, *J* = 7.4 Hz, O-CH_2_*-CH_2_*-CH_2_-N); 2.60–2.70 (m, 4H, 2 × CH_2_ piperidine); 2.73 (t, 2H, *J* = 7.6 Hz, CH_2_-N); 4.24 (t, 2H, *J* = 6.2 Hz, O-CH_2_); 6.79 (d, 1H, *J* = 5.2 Hz, Ar); 7.49 (t, 1H, *J* = 7.8 Hz, Ar); 8.00 (d, 1H, *J* = 7.2 Hz, Ar); 8.36 (d, 1H, *J* = 8.4 Hz, Ar); 8.83 (d, 1H, *J* = 5.2 Hz, Ar). ^13^C-NMR (100 MHz, CDCl_3_) δ 23.8; 25.1; 25.7; 54.4; 55.5; 66.9; 101.5; 122.0; 122.8; 124.1; 125.6; 126.5; 128.2; 145.7; 152.3; 161.3. ESI-MS calcd. for C_18_H_21_F_3_N_2_O, 338.37; found: *m*/*z* 339.16 [M + H]^+^. Anal. C_18_H_21_F_3_N_2_O (C, H, N) calcd. C, 61.18; H, 6.28; N, 11.89; found: C, 61.42; H, 6.30; N, 11.94.

*N,N-Diethyl-3-{[8-(trifluoromethyl)quinolin-4-yl]oxy}propan-1-amine* (**18d**). Diethylamine (commercially available) was used as the reagent. Reaction time: 24 h. Yield = 46%; oil. ^1^H-NMR (400 MHz, CDCl_3_) δ 1.15 (t, 6H, *J* = 7.2 Hz, 2 × CH_3_); 2.22 (quin, 2H, *J* = 7.0 Hz, O-CH_2_-*CH_2_-*CH_2_-N); 2.75 (q, 4H, *J* = 7.2 Hz, 2 × *CH_2_*CH_3_); 2.86 (t, 2H, *J* = 7.4 Hz, CH_2_-N); 4.28 (t, 2H, *J* = 6.0 Hz, O-CH_2_); 6.82 (d, 1H, *J* = 5.2 Hz, Ar); 7.51 (t, 1H, *J* = 7.8 Hz, Ar); 8.02 (d, 1H, *J* = 7.2 Hz, Ar); 8.37 (d, 1H, *J* = 8.4 Hz, Ar); 8.86 (d, 1H, *J* = 5.2 Hz, Ar). ^13^C-NMR (100 MHz, CDCl_3_) δ 10.6; 25.7; 46.9; 49.1; 66.6; 101.6; 121.9; 122.8; 124.2; 125.8; 126.9; 128.3; 145.6; 152.4; 161.3. ESI-MS calcd. for C_17_H_21_F_3_N_2_O, 326.36; found: *m*/*z* 327.16 [M + H]^+^. Anal. C_17_H_21_F_3_N_2_O (C, H, N) calcd. C, 63.89; H, 6.26; N, 8.28; found: C, 63.63; H, 6.23; N, 8.25.

*4-{3-[(8-(Trifluoromethyl)quinolin-4-yl)oxy]propyl}morpholine* (**18e**). Morpholine (commercially available) was used as the reagent. Reaction time: 24 h. Yield = 64%; oil. ^1^H-NMR (400 MHz, CDCl_3_) δ 2.13 (quin, 2H, *J* = 6.8 Hz, O-CH_2_-*CH_2_*-CH_2_-N); 2.45–2.55 (m, 4H, 2 × CH_2_ morpholine); 2.60 (t, 2H, *J* = 7.2 Hz, CH_2_-N); 3.72 (t, 4H, *J* = 4.6 Hz, 2 × CH_2_ morpholine); 4.28 (t, 2H, *J* = 6.4 Hz, O-CH_2_); 6.81 (d, 1H, *J* = 5.2 Hz, Ar); 7.51 (t, 1H, *J* = 7.8 Hz, Ar); 8.03 (d, 1H, *J* = 7.2 Hz, Ar); 8.40 (d, 1H, *J* = 8.0 Hz, Ar); 8.86 (d, 1H, *J* = 5.2 Hz, Ar). ^13^C-NMR (100 MHz, CDCl_3_) δ 25.9; 53.7; 55.3; 66.8; 66.9; 101.5; 122.1; 122.9; 124.0; 125.6; 126.5; 128.1; 145.7; 152.3; 161.5. ESI-MS calcd. for C_17_H_19_F_3_N_2_O_2_, 340.35; found: *m*/*z* 341.14 [M + H]^+^. Anal. C_17_H_19_F_3_N_2_O_2_ (C, H, N) calcd. C, 62.56; H, 6.49; N, 8.58; found: C, 62.31; H, 6.46; N, 8.54.

*Tert-Butyl 4-{3-[(8-(trifluoromethyl)quinolin-4-yl)oxy]propyl}piperazine-1-carboxylate* (**18f**). Tert-butylpiperazine-1-carboxylate (commercially available) was used as the reagent. Reaction time: 72 h. Yield = 46%; oil. ^1^H-NMR (400 MHz, CDCl_3_) δ 1.45 (s, 9H, C(CH_3_)_3_); 2.15 (quin, 2H, *J* = 6.6 Hz, O-CH_2_-*CH_2_-*CH_2_-N); 2.40–2.55 (m, 4H, 2 × CH_2_ piperazine); 2.62 (t, 2H, *J* = 7.0 Hz, CH_2_-N); 3.40–3.55 (m, 4H, 2 × CH_2_ piperazine); 4.28 (t, 2H, *J* = 6.2 Hz, O-CH_2_); 6.82 (d, 1H, *J* = 5.2 Hz, Ar); 7.52 (t, 1H, *J* = 7.8 Hz, Ar); 8.04 (d, 1H, *J* = 7.2 Hz, Ar); 8.40 (d, 1H, *J* = 8.4 Hz, Ar); 8.87 (d, 1H, *J* = 5.2 Hz, Ar). ESI-MS calcd. for C_22_H_28_F_3_N_3_O_3_, 439.48; found: *m*/*z* 440.21 [M + H]^+^. Anal. C_22_H_28_F_3_N_3_O_3_ (C, H, N) calcd. C, 59.99; H, 5.63; N, 8.23; found: C, 59.75; H, 5.61; N, 8.20.

*General procedure for compounds* **17g** *and* **18g**. A mixture of **17f** (for **17g**) or **18f** (for **18g**) (0.08 mmol), trifluoroacetic acid (0.28 mL) and CH_2_Cl_2_ (1.72 mL) was stirred at room temperature for 2 h. After evaporation of the solvent, ice/cold water was added, and the pH was adjusted to 9 with NaOH 6M. The suspension was extracted with ethyl acetate (3 × 15 mL), dried on sodium sulphate and evaporated, and the solid was obtained, which was washed with petroleum ether to give the pure compound.

*4-[3-(Piperazin-1-yl)propoxy]-2,8-bis(trifluoromethyl)quinoline* (**17g**). Yield = 31%; mp = 74–76 °C (EtOH). ^1^H-NMR (400 MHz, CDCl_3_) δ 2.16 (t, 2H, *J* = 6.4 Hz, O-CH_2_-*CH_2_-*CH_2_-N); 2.50–2.60 (m, 4H, 2 × CH_2_ piperazine); 2.64 (t, 2H, *J* = 7.0 Hz, CH_2_-N); 2.95–3.05 (m, 4H, 2 × CH_2_ piperazine); 4.35 (t, 2H, *J* = 5.8 Hz, O-CH_2_); 7.13 (s, 1H, Ar); 7.64 (t, 1H, *J* = 7.8 Hz, Ar); 8.12 (d, 1H, *J* = 6.8 Hz, Ar); 8.43 (d, 1H, *J* = 8.4 Hz, Ar). ^13^C-NMR (100 MHz, CDCl_3_) δ 26.0; 44.3; 51.6; 54.7; 67.2; 97.6; 116.4; 120.4; 122.2; 126.1; 126.2; 128.1; 129.3; 144.8; 146.6; 162.2. ESI-MS calcd. for C_18_H_19_F_6_N_3_O, 407.36; found: *m*/*z* 408.15 [M + H]^+^. Anal. C_18_H_19_F_6_N_3_O (C, H, N) calcd. C, 53.07; H, 4.70; N, 10.32; found: C, 53.28; H, 4.72; N, 10.36.

*4-[3-(Piperazin-1-yl)propoxy]-8-(trifluoromethyl)quinoline* (**18g**). Yield = 84%; oil. ^1^H-NMR (400 MHz, CDCl_3_) δ 2.15 (quin, 2H, *J* = 6.6 Hz, O-CH_2_-*CH_2_*-CH_2_-N); 2.75 (t, 2H, *J* = 6.8 Hz, CH_2_-N); 2.80–2.85 (m, 4H, 2 × CH_2_ piperazine); 3.20–3.30 (m, 4H, 2 × CH_2_ piperazine); 4.28 (t, 2H, *J* = 6.0 Hz, O-CH_2_); 6.83 (d, 1H, *J* = 5.2 Hz, Ar); 7.55 (t, 1H, *J* = 8.0 Hz, Ar); 8.07 (d, 1H, *J* = 7.2 Hz, Ar); 8.40 (d, 1H, *J* = 8.4 Hz, Ar); 8.89 (d, 1H, *J* = 5.2 Hz, Ar). ^13^C-NMR (100 MHz, CDCl_3_) δ 25.6; 43.1; 49.4; 54.4; 66.1; 101.5; 122.1; 124.3; 124.4; 126.3; 127.0; 128.3; 145.7; 152.3; 161.2. ESI-MS calcd. for C_17_H_20_F_3_N_3_O, 339.36; found: *m*/*z* 340.16 [M + H]^+^. Anal. C_17_H_20_F_3_N_3_O (C, H, N) calcd. C, 60.17; H, 5.94; N, 12.38; found: C, 60.41; H, 5.96; N, 12.43.

*General procedure for the synthesis of compounds* **20b**,**c**. To a mixture of intermediate **19** [26] (0.40 mmol) in 12 mL of EtOH abs., 0.44 mmol of suitable amine (see Supporting Information) and 1.00 mmol of DIPEA were added. The mixture was stirred at reflux for 14 h; then, after evaporation of the solvent, the crude product was purified by flash column chromatography using dichloromethane/methanol/ammonia 90:10:1 (for **20b**) or 95:5:0.5 (for **20c**) as eluents.

*N-(3-(4-Methylpiperazin-1-yl)propyl)-2,8-bis(trifluoromethyl)quinolin-4-amine* (**20b**). 3-(4-methylpiperazin-1-yl)propan-1-amino [34] was used as the reagent. Yield = 23%; oil. ^1^H-NMR (400 MHz, CDCl_3_) δ 2.03 (quin, 2H, *J* = 5.6 Hz, HN-CH_2_-*CH_2_*-CH_2_-N); 2.43 (s, 3H, CH_3_ piperazine); 2.60–2.70 (m, 8H, 4 × CH_2_ piperazine); 2.75 (t, 2H, *J* = 5.2 Hz, CH_2_-N- piperazine); 3.47 (q, 2H, *J* = 5.2 Hz, HN-*CH_2_*); 6.68 (s, 1H, Ar); 7.51 (t, 1H, *J* = 7.8 Hz, Ar); 7.96 (exch br s, 1H, NH); 8.03 (d, 1H, *J* = 7.2 Hz, Ar); 8.26 (d, 1H, *J* = 8.4 Hz, Ar). ^13^C-NMR (100 MHz, CDCl_3_) δ 22.9; 45.9; 52.9; 54.3; 57.7; 94.4; 119.9; 120.1; 124.1; 125.4; 126.2; 128.4; 144.6; 151.9; 156.4; 156.8. ESI-MS calcd. for C_19_H_22_F_6_N_4_, 420.40; found: *m*/*z* 421.18 [M + H]^+^. Anal. C_19_H_22_F_6_N_4_ (C, H, N) calcd. C, 54.28; H, 5.27; N, 13.33; found: C, 54.50; H, 5.29; N, 13.38.

*N-(3-(Piperidin-1-yl)propyl)-2,8-bis(trifluoromethyl)quinolin-4-amine* (**20c**). 3-(piperidin-1-yl)propan-1-amino [34] was used as the reagent. Yield = 25%; oil. ^1^H-NMR (400 MHz, CDCl_3_ + D_2_O) δ 1.89 (t, 4H, *J* = 5.4 Hz, 2 × CH_2_ piperidine); 2.05–2.10 (m, 2H, CH_2_ piperidine); 2.15–2.20 (m, 2H, HN-CH_2_-*CH_2_-*CH_2_-N); 2.80–2.90 (m, 4H, 2 × CH_2_ piperidine); 2.96 (t, 2H, *J* = 6.4 Hz, CH_2_-N-piperidine); 3.49 (t, 2H, *J* = 5.4 Hz, HN-*CH_2_*); 6.64 (s, 1H, Ar); 7.55 (t, 1H, *J* = 7.8 Hz, Ar); 8.03 (d, 1H, *J* = 7.2 Hz, Ar); 8.61 (d, 1H, *J* = 8.4 Hz, Ar). ^13^C-NMR (100 MHz, CDCl_3_) δ 22.4; 22.8; 23.6; 40.9; 53.7; 55.7; 94.1; 119.9; 122.4; 124.5; 125.3; 126.2; 128.5; 144.5; 151.9. ESI-MS calcd. for C_19_H_21_F_6_N_3_, 405.39; found: *m*/*z* 406.17 [M + H]^+^. Anal. C_19_H_21_F_6_N_3_ (C, H, N) calcd. C, 56.29; H, 5.22; N, 10.37; found: C, 56.51; H, 5.24; N, 10.41.

### 3.2. Biological Test

*Electrophysiology*. Chinese hamster ovary (CHO; ATCC #CCL-61) cells were seeded onto glass chips; then, *mPanx1* and GFP cDNA (0.5 µg each; Origene) were transfected as described earlier [35,36]. Electrophysiological experiments were performed on the eGFP-positive cells 48–96 h after transfection. Whole-cell macroscopic current recordings of pannexin-1 were made under voltage-clamp conditions with a ramp protocol (90**→** −90 mV). The pipette solution contained the following (in mM): 120 CsCl_2_, 5 NaCl, 2 MgCl_2_, 5 EGTA, 2 Mg-ATP, 0.1 GTP, and 10 mM HEPES (pH 7.4); the extracellular solution contained 140 mM NaCl, 3mM KCl, 2 mM MgCl_2_, 2 mM CaCl_2_, and 10 mM HEPES (pH 7.4). Current recordings were acquired with an Axopatch 200B amplifier interfaced via a Digidata 1550B (Molecular Devices, Sunnyvale, CA, USA); all currents were filtered at 1 kHz. Whole-cell capacitance (average values of ~4–10 pF) and series resistances (average values ~2–5 MOhm) were compensated. The inhibitory effect of the drugs on Panx1-dependent currents is reported in mean% ± SEM. IC50 was calculated using the Y = 100/(1 + X/IC50) model with GraphPad Prism 10’s [inhibitor vs. normalized response] function.

*Cyst development assay*. Mouse collecting duct (mpkCCD_cl4_) cells (PMID: 10232677, 37024297) were plated on a 24-well plate (Corning 3598, Corning, NY, USA) in a 1:1 mixture of Matrigel (Corning 356234) and culture media DMEM:F12 (10-090-CV) containing 2% FBS, 1% insulin–transferrin–selenium mix (Gibco 41400-045, Grand Island, NY, USA), 1% Penicillin–Streptomycin Glutamine (Gibco 10378-016) with 10 µM Forskolin (Tocris 1099, Bristol, UK) and a vehicle/RFs. Cysts were grown for 3 weeks with the addition and replacement of the media, covering Matrigel, and treatment. Individual cyst images were captured at 40× magnification, and cyst size was measured using the Fiji software package (NIH, Bethesda, MD, USA, https://imagej.net/software/fiji/downloads (accessed on 11 May 2025)) by manual selection of each cyst wall border.

### 3.3. Molecular Modeling

All the 3D structures of the molecules were designed with DS ViewerPro 6.0 [37].

*Molecular Docking.* For molecular docking, we used the AUTODOCK suite [31]. Docking studies were performed considering the entire structure of the 7DWB channel. The search grid (GRID module), 37 × 37 × 49 Å (spacing 0.46 Å), was initially built in the center of the channel and subsequently on the extracellular portion. The main parameters used for docking (AutoDock operating conditions) in the ECD portion are reported in the Supporting Information. From AutoDock, 100 conformations of the complex (poses) were obtained and collected into conformational similarity classes within 10 Root Mean Square (RMS).

*Complex potential energy Minimization.* For the potential energy minimization of the ligand–protein complex, GROMACS was used (version 2023 https://doi.org/10.5281/zenodo.7588619) [38]. The entire solvated complex was considered. The partial atomic charge of the ligand structures was calculated with CHIMERA [39] using the AM1-BCC method, and the topology was created with ACPYPE [40] based on the routine Antechamber. For the potential energy calculation, the AMBER99sb force field parameters were applied. A steepest descent algorithm for energy minimization was used with a tolerance of 10.0 kJ mol^−1^ nm^−1^ (the minimization is converged when the maximum force is smaller than this value). The main parameters used for energy minimization (GROMACS operating conditions) are reported in the Supporting Information.

### 3.4. Statistical Analysis

*Chemometric methods.* The chemometric approach was based on multiple linear regression (MLR). A genetic algorithm (GA) was used to find the best combination of variables. *GA* is a metaheuristic method inspired by natural selection processes and is commonly applied to find high-quality solutions to complex problems. The algorithm evolves a set of solutions to a problem towards improved outcomes; each solution consists of a combination of variables that can be modified and altered. The iterative process begins with a set (populations) of randomly generated combinations of variables (individuals). Each individual in the population is evaluated (fitness), and those with better performance are selected. The selected individuals undergo genetic operations such as crossover (also called recombination) and mutation, generating a new population (generation). This process is repeated until a predefined maximum number of generations is produced. The adopted GA is represented by the flowchart reported in the Supporting Information.

## 4. Conclusions

In this manuscript, we report the synthesis and the biological evaluation of two different series of compounds as Panx1 blockers: new 3-carboxy-6-sulphonamidoquinolones as an elaboration of the already-published 3-carboxy-6-nitroquinolones and new analogs of Mefloquine, a well-known antimalarial agent but also a potent and selective Panx1 inhibitor. Mefloquine-based compounds turned out to have very low activity or be completely inactive (I% < 20%), whereas the new 6-sulphonamide quinolones showed interesting results. From the biological data, it emerges that the replacement of the reference compounds of type 6 of the nitro group at position 6 with a primary sulphonamide moiety is detrimental for activity, but moving to the tertiary sulphonamide, a marked increase in potency is recorded; moreover, a fundamental element for activity is the acid group at R_1_, such as COOH and SO_2_NH_2_. The combination of these two elements has resulted in powerful Panx1 channel blockers with 73.2 < I% < 100 at 50 µM (**7**, **12b**, **12e** and **12f**), thus comparable to or greater than the reference compounds.

For compounds **7**, **12e** and **12f**, the IC_50_ values were calculated, and for **12f**, they were lower than those of CBX (IC_50_ = 2.7 µM versus IC_50_ = 7.1 µM); moreover, the most potent products **12e** and **12f**, tested during a 3-week period at 10 µM and 3 µM, respectively, in a cell culture model of polycystic kidney disease, reduced the size of cysts by about 13%, thus confirming the therapeutic potential of Panx1 blockers on cystogenesis.

Finally, in this work, we have acquired additional information about the binding site of Panx1 channel blockers. In fact, we previously evidenced [16,17,18] that our active compounds were able to block the channel at the top of the extracellular domain, analogous to CBX. Instead, in this study, we have observed that the more active molecules (**12e**, **12f** and Mef) accumulated in the deep part of the extracellular domain and formed strong hydrogen bond interactions with Trp74 and Cys434, differently from the inactive **12d**. Thus, in conclusion, this study has provided other information on the discovery of the Panx1 blocker’s binding site and the configuration of the channel, formed by seven identical protomers, making this research intriguing.

## Figures and Tables

**Table 1:** Inhibition % values of compounds **5a**,**b**, **7** and **12a**–**g**.

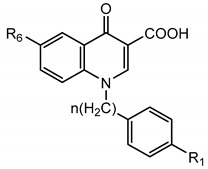				
Compounds	R_6_	R_1_	n	I% ^a^
**5a**	SO_2_NH_2_	H	1	27.1 ± 4.4
**5b**	SO_2_NH_2_	COOH	1	48.8 ± 5.9
**7**	SO_2_NCH_2_-(Ph-4-COOH)_2_	COOH	1	79.7 ± 7.2
**12a**	SO_2_N(CH_2_CH_3_)_2_	H	1	54.6 ± 6.0
**12b**	SO_2_N(CH_2_CH_3_)_2_	COOH	1	73.2 ± 11.9
**12c**	SO_2_N(CH_2_CH_3_)_2_	SO_2_NH_2_	1	49.5 ± 6.0
**12d**	SO_2_N(CH_2_CH_2_CH_3_)_2_	H	1	10.4 ± 7.7
**12e**	SO_2_N(CH_2_CH_2_CH_3_)_2_	COOH	1	84.8 ± 4.4
**12f**	SO_2_N(CH_2_CH_2_CH_3_)_2_	SO_2_NH_2_	1	100 ± 0
**12g**	SO_2_N(CH_2_CH_2_CH_3_)_2_	H	2	57.2 ± 8.5
**6a** [18]	NO_2_	H	1	53 ± 2.9
**6g** [18]	NO_2_	COOH	1	89.0 ± 1.7
**CBX ^b^**				80

^a^ Compounds were tested using the whole-cell technique in CHO mammalian cells expressing the mPanx-1 channel at 50 µM; n = 5–6. ^b^ CBX at 15 µM.

**Table 2:** Independent variables for MLR. V1–V17: hydrogen bond lengths between ligands and amino acids. V18–V31: hydrogen bond lengths between ligands and the amino acid-bridged water molecule.

Code	Aminoacidi
V1	ser59
V2	ile60
V3	gly61
V4	thr62
V5	ser68
V6	ser70
V7	ser71
V8	phe72
V9	ser73
V10	trp74
V11	arg75
V12	asp81
V13	ser82
V14	asn420
V15	gly421
V16	glu422
V17	cys434
V18	Wser59
V19	Wile60
V20	Wgly61
V21	Wthr62
V22	Wpro69
V23	Wser70
V24	Wser73
V25	Wtrp74
V26	Warg75
V27	Wtrp85
V28	Wglu415
V29	Wasn420
V30	Wgly421
V31	Wcys434

**Table 3:** Regression coefficients of the multiple linear equations and corresponding statistical parameters (asterisks denote the statistical significance of the regression coefficient).

*Variable*	*Coefficient*	*St. Error*	*t*	*p ^a^*
(Intercept)	−4.801	67.718	−0.071	0.943886
V8 (phe 72)	**16.513**	7.103	2.325	0.026003 *
V19	10.565	6.697	1.577	0.123687
V24 (Wser 73)	**36.231**	9.371	3.866	0.000459 ***
V27	−8.328	7.838	−1.062	0.295289
V17 (cys 434)	**−27.533**	4.615	−5.966	8.56 × 10^−7^ ***
V26 (Warg 75)	**9.131**	2.880	3.171	0.003155 **
V11	−6.254	4.125	−1.516	0.138445
V10 (trp 74)	**−19.747**	2.867	−6.888	5.32 × 10^−8^ ***

^a^ * *p* < 0.05, ** *p* < 0.005, *** *p* < 0.0005.

## Data Availability

The data supporting this article can be found online as part of the Supplementary Materials.

## Supplementary Materials

The following supporting information can be downloaded at https://www.mdpi.com/article/10.3390/molecules30102171/s1: Chemistry; molecular modeling (conditions for Autodock and Gromacs, Tables S1 and S2); Table S3: The actual and predicted values of I% and the absolute errors; NMR spectra of some representative compounds; elementary analysis; validation of electrophysiological approach. Figure S1: Cartoon of structure of h-PANX1 (7DWB). Figures S2–S4: Docking images of more representative compounds (**7**, **12e** and **12f**).

## References

[1] Panchin Y., Kelmanson I., Matz M., Lukyanov K., Usman N., Lukyanov S. (2000). A Ubiquitous Family of Putative Gap Junction Molecules. Curr. Biol..

[2] Penuela S., Gehi R., Laird D.W. (2013). The Biochemistry and Function of Pannexin Channels. Biochim. Biophys. Acta—Biomembr..

[3] Zhang S., Yuan B., Lam J.H., Zhou J., Zhou X., Ramos-Mandujano G., Tian X., Liu Y., Han R., Li Y. (2021). Structure of the Full-Length Human Pannexin1 Channel and Insights into Its Role in Pyroptosis. Cell Discov..

[4] Michalski K., Syrjanen J.L., Henze E., Kumpf J., Furukawa H., Kawate T. (2020). The Cryo-EM Structure of a Pannexin 1 Reveals Unique Motifs for Ion Selection and Inhibition. eLife.

[5] Ruan Z., Orozco I.J., Du J., Lü W. (2020). Structures of Human Pannexin 1 Reveal Ion Pathways and Mechanism of Gating. Nature.

[6] Mim C., Perkins G., Dahl G. (2021). Structure versus Function: Are New Conformations of Pannexin 1 yet to Be Resolved?. J. Gen. Physiol..

[7] Hussain N., Apotikar A., Pidathala S., Mukherjee S., Burada A.P., Sikdar S.K., Vinothkumar K.R., Penmatsa A. (2024). Cryo-EM Structures of Pannexin 1 and 3 Reveal Differences among Pannexin Isoforms. Nat. Commun..

[8] López X., Palacios-Prado N., Güiza J., Escamilla R., Fernández P., Vega J.L., Rojas M., Marquez-Miranda V., Chamorro E., Cárdenas A.M. (2021). A Physiologic Rise in Cytoplasmic Calcium Ion Signal Increases Pannexin1 Channel Activity via a C-Terminus Phosphorylation by CaMKII. Proc. Natl. Acad. Sci. USA.

[9] Dahl G. (2015). ATP Release through Pannexon Channels. Philos. Trans. R. Soc. B Biol. Sci..

[10] Boyce A.K.J., Kim M.S., Wicki-Stordeur L.E., Swayne L.A. (2015). ATP Stimulates Pannexin 1 Internalization to Endosomal Compartments. Biochem. J..

[11] Boyce A.K.J., Swayne L.A. (2017). P2X7 Receptor Cross-Talk Regulates ATP-Induced Pannexin 1 Internalization. Biochem. J..

[12] Baracaldo-Santamaría D., Corrales-Hernández M.G., Ortiz-Vergara M.C., Cormane-Alfaro V., Luque-Bernal R.-M.M., Calderon-Ospina C.-A.A., Cediel-Becerra J.-F.F., Gabriela Corrales-Hernández M., Camila Ortiz-Vergara M., Cormane-Alfaro V. (2022). Connexins and Pannexins: Important Players in Neurodevelopment, Neurological Diseases, and Potential Therapeutics. Biomedicines.

[13] Seo J.H., Dalal M.S., Contreras J.E. (2021). Pannexin-1 Channels as Mediators of Neuroinflammation. Int. J. Mol. Sci..

[14] Arkhipov S.N., Potter D.L., Sultanova R.F., Ilatovskaya D.V., Harris P.C., Pavlov T.S. (2023). Probenecid Slows Disease Progression in a Murine Model of Autosomal Dominant Polycystic Kidney Disease. Physiol. Rep..

[15] Arkhipov S.N., Pavlov T.S. (2019). ATP Release into ADPKD Cysts via Pannexin-1/P2X7 Channels Decreases ENaC Activity. Biochem. Biophys. Res. Commun..

[16] Crocetti L., Guerrini G., Puglioli S., Giovannoni M.P., Di Cesare Mannelli L., Lucarini E., Ghelardini C., Wang J., Dahl G. (2021). Design and Synthesis of the First Indole-Based Blockers of Panx-1 Channel. Eur. J. Med. Chem..

[17] Crocetti L., Guerrini G., Giovannoni M.P., Melani F., Lamanna S., Mannelli L.D.C., Lucarini E., Ghelardini C., Wang J., Dahl G. (2022). New Panx-1 Blockers: Synthesis, Biological Evaluation and Molecular Dynamic Studies. Int. J. Mol. Sci..

[18] Crocetti L., Giovannoni M.P., Guerrini G., Lamanna S., Melani F., Bartolucci G., Pallecchi M., Paoli P., Lippi M., Wang J. (2023). Design, Synthesis and Pharmacological Evaluation of New Quinoline-Based Panx-1 Channel Blockers. Int. J. Mol. Sci..

[19] Matada B.S., Pattanashettar R., Yernale N.G. (2021). A Comprehensive Review on the Biological Interest of Quinoline and Its Derivatives. Bioorganic Med. Chem..

[20] Koval M., Schug W.J., Isakson B.E. (2023). Pharmacology of Pannexin Channels. Curr. Opin. Pharmacol..

[21] Price C.C., Leonard N.J., Stacy G.W. (1947). The Synthesis of 4-Hydroxyquinolines. X. Quinoline Derivatives with Sulfur- Containing Substituents. J. Am. Chem. Soc..

[22] Yun F., Cheng C., Ullah S., Yuan Q. (2020). Design, Synthesis and Biological Evaluation of Novel Histone Deacetylase1/2 (HDAC1/2) and Cyclin-Dependent Kinase2 (CDK2) Dual Inhibitors against Malignant Cancer. Eur. J. Med. Chem..

[23] Zhang H., Daněk O., Makarov D., Rádl S., Kim D., Ledvinka J., Vychodilová K., Hlaváč J., Lefèbre J., Denis M. (2022). Drug-like Inhibitors of DC-SIGN Based on a Quinolone Scaffold. ACS Med. Chem. Lett..

[24] Serkov S.A., Sigay N.V., Kostikova N.N., Tyurin A.P., Kolotyrkina N.G., Gazieva G.A. (2024). Synthesis of 5-Oxo-1-[4-(Aminosulfonyl)Phenyl]Pyrrolidine-3-Carboxylic Acids. Russ. Chem. Bull..

[25] Daha F.J., Matloubi H., Tabatabi S.A., Shafii B., Shafiee A. (2005). Syntheses of 1-(4-Methylsulfonylphenyl)-5-Aryl-1,2,3-Triazoles and -(4-Aminosulfonylphenyl)-5-Aryl-1,2,3-Triazoles. J. Heterocycl. Chem..

[26] Eswaran S., Adhikari A.V., Chowdhury I.H., Pal N.K., Thomas K.D. (2010). New Quinoline Derivatives: Synthesis and Investigation of Antibacterial and Antituberculosis Properties. Eur. J. Med. Chem..

[27] Liebman K.M., Burgess S.J., Gunsaru B., Kelly J.X., Li Y., Morrill W., Liebman M.C., Peyton D.H. (2020). Unsymmetrical Bisquinolines with High Potency against P. Falciparum Malaria. Molecules.

[28] Yang G.Z., Zhu J.K., Yin X.D., Yan Y.F., Wang Y.L., Shang X.F., Liu Y.Q., Zhao Z.M., Peng J.W., Liu H. (2019). Design, Synthesis, and Antifungal Evaluation of Novel Quinoline Derivatives Inspired from Natural Quinine Alkaloids. J. Agric. Food Chem..

[29] Ma W., Hui H., Pelegrin P., Surprenant A. (2009). Pharmacological Characterization of Pannexin-1 Currents Expressed in Mammalian Cells. J. Pharmacol. Exp. Ther..

[30] Shum M.G., Shao Q., Lajoie P., Laird D.W. (2019). Destination and Consequences of Panx1 and Mutant Expression in Polarized MDCK Cells. Exp. Cell Res..

[31] Morris G.M., Ruth H., Lindstrom W., Sanner M.F., Belew R.K., Goodsell D.S., Olson A.J. (2009). Software News and Updates AutoDock4 and AutoDockTools4: Automated Docking with Selective Receptor Flexibility. J. Comput. Chem..

[32] Pronk S., Páll S., Schulz R., Larsson P., Bjelkmar P., Apostolov R., Shirts M.R., Smith J.C., Kasson P.M., Van Der Spoel D. (2013). GROMACS 4.5: A High-Throughput and Highly Parallel Open Source Molecular Simulation Toolkit. Bioinformatics.

[33] Türkeş C., Arslan M., Demir Y., Çoçaj L., Rifati Nixha A., Beydemir Ş. (2019). Synthesis, Biological Evaluation and in Silico Studies of Novel N-Substituted Phthalazine Sulfonamide Compounds as Potent Carbonic Anhydrase and Acetylcholinesterase Inhibitors. Bioorg. Chem..

[34] Soliman B., Wang N., Zagotto G., Pockes S. (2019). Synthesis and Biological Evaluation of Heteroalicyclic Cyanoguanidines at Histamine Receptors. Arch. Pharm..

[35] Staruschenko A., Booth R.E., Pochynyuk O., Stockand J.D., Tong Q. (2006). Functional Reconstitution of the Human Epithelial Na+ Channel in a Mammalian Expression System. Methods Mol. Biol..

[36] Doerr N., Wang Y., Kipp K.R., Liu G., Benza J.J., Pletnev V., Pavlov T.S., Staruschenko A., Mohieldin A.M., Takahashi M. (2016). Regulation of Polycystin-1 Function by Calmodulin Binding. PLoS ONE.

[37] (2005). DS ViewerPro.

[38] Pall S., Abraham M.J.M.J., Kutzner C., Hess B., Lindahl E., Szilárd P., Abraham M.J.M.J., Kutzner C., Hess B., Lindahl E., Markidis S., Laure E. (2015). Tackling Exascale Software Challenges in Molecular Dynamics Simulations with GROMACS. Solving Software Challenges for Exascale 8759.

[39] Pettersen E.F., Goddard T.D., Huang C.C., Couch G.S., Greenblatt D.M., Meng E.C., Ferrin T.E. (2004). UCSF Chimera—A Visualization System for Exploratory Research and Analysis. J. Comput. Chem..

[40] Sousa Da Silva A.W., Vranken W.F. (2012). ACPYPE—AnteChamber PYthon Parser InterfacE. BMC Res. Notes.

