# Supplementary material for: Synthesis of 3-Carboxy-6-sulfamoylquinolones and Mefloquine-Based Compounds as Panx1 Blockers: Molecular Docking, Electrophysiological and Cell Culture Studies
Source: Molecules. 2025 May 15;30(10):2171. doi: 10.3390/molecules30102171 (PMC12114274; doi:10.3390/molecules30102171)

**Supporting Information**

**For**

# Synthesis of 3-carboxy-6-sulfamoylquinolones and Mefloquine-based compounds as Panx1 blockers: synthesis, molecular docking, electrophysiological and cell culture studies

Letizia Crocetti[a], Maria Paola Giovannoni[a], Tengis S. Pavlov[b], Veniamin Ivanov[b], Melani Fabrizio[a], Gabriella Guerrini[a*]

[a] Neurofarba, Pharmaceutical and Nutraceutical Section, University of Florence, Via Ugo Schiff 6, 50019 Sesto Fiorentino, Italy

[b] Division of Hypertension and Vascular Research, Henry Ford Health & Wayne State University, 6135 Woodward Ave, Detroit, MI 48202, USA

*Correspondence: gabriella.guerrini@unifi.it; Tel.: +39-055-4573766

**Table of contents**

1. Chemistry
2. Fig. S1: Cartoon of structure of h-PANX1 (7DWB)
3. Molecular Modeling (Conditions for Autodock and Gromacs, Table S1 and S2)
4. Table S3: The actual and predicted values of I% and the absolute errors.
5. Fig. S2-4: Docking images of more representative compounds (7, 12e and 12f)
6. NMR spectra of some representative compounds
7. Elementary Analysis
8. Validation of the electrophysiological approach

1. **Chemistry**

To obtain the final compounds **20b,c** reported in the main manuscript, we have synthesized the suitable basic chains of type **4** following a procedure reported in literature (Soliman et al. 2019). Starting from the commercial compound **1**, which is first protected with Boc (**2**) and then reacted with the appropriate amine in DCM/$Et_3N$ providing the intermediates **3a,b**. Nitrogen deprotection on compounds **3a,b** using a mixture of DCM/$CF_3COOH$ led to the desired basic chains **4a,b** (Soliman et al. 2019).

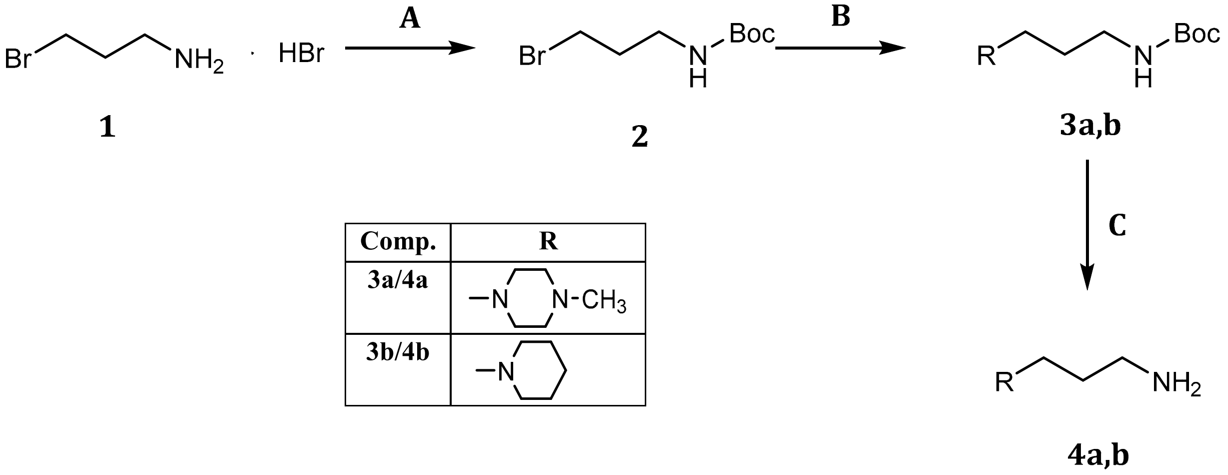


**Reagents and Conditions:** **A)** DCM dry, Boc, $Et_3N$, r.t., 2 h; **B)** DCM dry, appropriate amine, $Et_3N$, 70 °C, 14 h; **C)** DCM/$CF_3COOH$ 4:1, r.t., 2 h.

## 2. Cartoon of structure of h-PANX1 (7DWB)

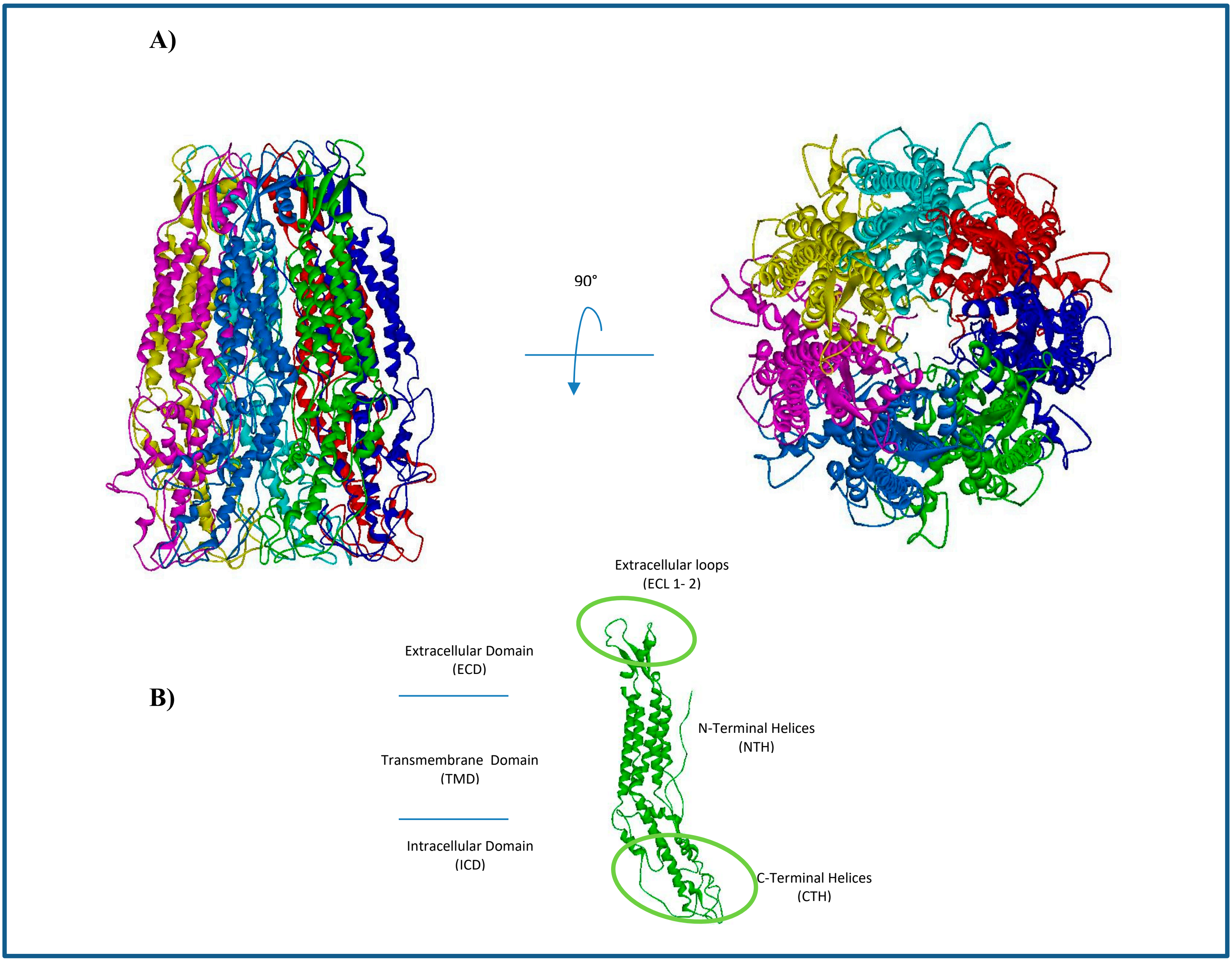


Fig S1. A) Cartoon of the hPanx1 channel (7DWB) showing the seven protomers in different colours. B) Schematic representation of the secondary structure of the single chain.

## 3. Molecular Modeling

AutoDock (operating conditions)

GRID:

```
npts 80 80 106                       # num.grid points in xyz
spacing 0.46                         # spacing(A)
gridcenter 108.0 108.0 79.0          # xyz-coordinates or auto
```

DOCK:

```
ga_pop_size 150            # number of individuals in the population
ga_num_evals 2500000       # maximum number of energy evaluations
ga_num_generations 27000   # maximum number of generations
ga_elitism 1               # number of top individuals
ga_mutation_rate 0.02      # rate of gene mutation
ga_crossover_rate 0.8      # rate of crossover
ga_run 100                 # do this many hybrid GA-LS runs
```

GROMACS version: 2023 (more significant parameters)

MINIMIZATION

```
integrator = steep
emtol = 1000.0
emstep = 0.01
nsteps = 50000
nstlist = 1
cutoff-scheme = Verlet
ns_type = grid
rlist = 1.2
coulombtype = PME
rcoulomb = 1.2
vdwtype = cutoff
vdw-modifier = force-switch
rvdw-switch  = 1.0
rvdw  = 1.2
pbc = xyz
DispCorr = no
```

**Flow-chart**

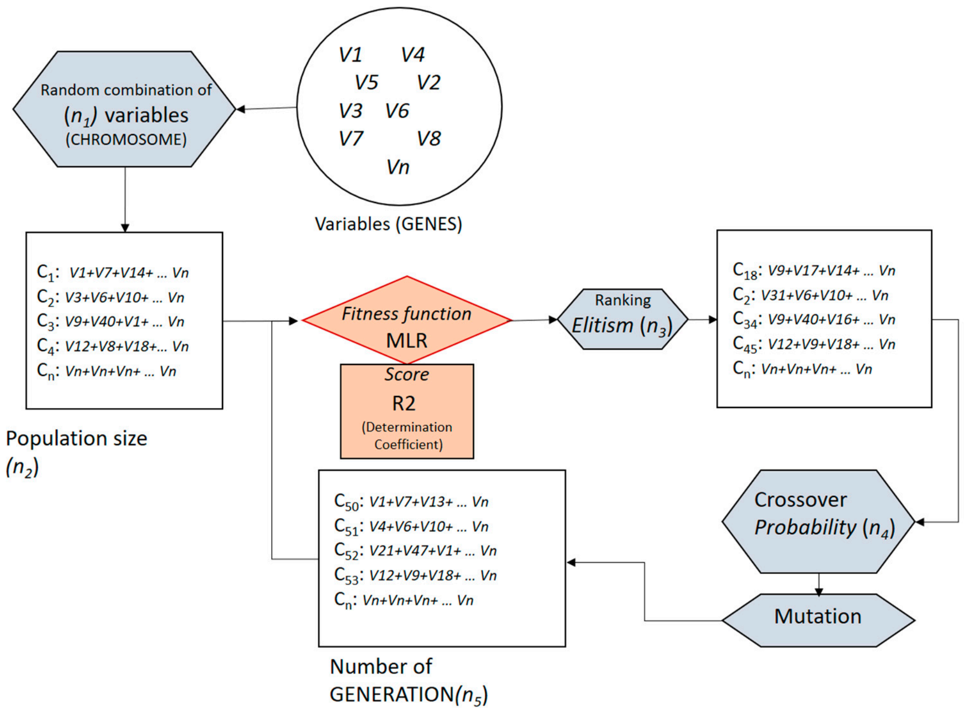


Multiple Linear Regression (MLR) is used as the fitness function and the "score" is given by the Determination Coefficient (R2).

The *GA* parameters are set as follows:

*($n_1$)* - Number of variables (genes) that constitute an "individual" (chromosome) : 8

*($n_2$)* - Number of "individuals" in the initial population (Population size): 100

*($n_3$)* - Number of elite "individuals" (Elitism): 50 ("individuals" with better performance).

*($n_4$)* - Crossover probability: 1 (all elite "individuals"). Mutation probability: random

*($n_5$)* - Number of Generations: 25.

An "individual" is a combination of n variables represented by hydrogen bond lengths.

The MLR was performed using the R package ("The R Foundation for Statistical Computing" 2023). This study correlates, by MLR, the hydrogen bond lengths, which represent the independent variables, with the activity expressed as percent inhibition (I%).

**Table S1.** Hydrogen bond lengths (Å) between amino acids and new compounds, related to percentage of inhibition (I%); for all ligands, the different poses (indicated by the underscore sign) and deprotonated form ($^-$) are reported.

| Ligand | I (%) | ser59 | ile60 | gly61 | thr62 | ser68 | ser70 | ser71 | phe72 | ser73 | trp74 | arg75 | asp81 | ser82 | asn420 | gly421 | glu422 | cys434 |
|---|---|---|---|---|---|---|---|---|---|---|---|---|---|---|---|---|---|---|
| **5a_1** | 27 | | | 2.45 | | | | | | | | 2.88 | 1.76 | | | | | |
| **5a_2** | 27 | | | 2.87 | | | | | | | | 2.95 | 1.73 | | | | | |
| **5a_3** | 27 | | | 2.97 | | | | | | | | 2.38 | 1.85 | | | | | |
| **5a_1$^-$** | 27 | | | 2.91 | | | | | | | | 1.95 | 2.24 | 1.93 | | | | |
| **5a_2$^-$** | 27 | | | | | | | | | | | 2.79 | 1.79 | | | | | |
| **5a_3$^-$** | 27 | | | 2.55 | | | | | | | | 1.79 | 1.94 | | | | | |
| **5b_1** | 49 | | | | | | | | | 2.98 | | | | | | | | |
| **5b_2** | 49 | | | | | | | | 1.84 | | 1.96 | 2.9 | | | | | | |
| **5b_1$^-$** | 49 | | | 2.32 | | | | | | | | 1.9 | 1.92 | 2.48 | | | | |
| **5b_2$^-$** | 49 | | | 2.32 | | | | | | | | 1.91 | 1.91 | 2.43 | | | | |
| **7_1** | 80 | | | | | | | | | | 2.03 | | | | | | | |
| **7_2** | 80 | | | | | | | | | 2.05 | 2.42 | | | | | | | |
| **7$^-$** | 80 | | | | | | | | | 1.63 | 1.85 | | | | | | | |
| **12a_1** | 55 | | | | 2.56 | | | | | | | 2.31 | | | | | | |
| **12a_2** | 55 | | | | | | | | | | | 1.94 | | | | | | |
| **12a_3** | 55 | | | | | | | | | | 2.56 | 2.82 | | | | | | |
| **12a_1$^-$** | 55 | | | | | | | | | | 2.27 | | | | | | | |
| **12a_2$^-$** | 55 | | | | | | | | | | 1.92 | | | | | | | |
| **12b** | 74 | | | | | | | | | | 2.67 | | | | | | | |
| **12b$^-$** | 74 | | | | | | | | | | 1.84 | | | | | | | |
| **12c_1** | 50 | | | | | | | | | 1.62 | | | | | | | | |
| **12c_2** | 50 | | | | | | | | | 1.63 | 1.99 | | | | | | | |
| **12c_1$^-$** | 50 | | | | | | | | | 1.68 | 2.56 | | | | | | | |
| **12c_2$^-$** | 50 | | | | | 2.69 | | | | 1.79 | 2.01 | | | | | | | |
| **12d** | 11 | | | | | | | | | | 2.59 | | | | | | | |
| **12d$^-$** | 11 | | | | | | | | | | | | | | | | | |
| **12e** | 85 | | | | | | | | | | 2.26 | | | | | | | |
| **12e$^-$** | 85 | | | | | | | | | | 2.96 | | | | | | | |
| **12f_1** | 80[a] | | | | | | | | | | 2.79 | | | | | | | |
| **12f_2** | 80[a] | | | | | | 2.66 | 1.93 | | | 2.56 | | | | | | | |
| **12f$^-$** | 80[a] | | | | | | | | | | 2.13 | | | | | | | |
| **12g** | 57 | | | | | | | | | | 2.49 | | | | | | | |
| **12g$^-$** | 57 | | | | | | | | | | 1.84 | | | | | | | |
| **17a_1** | 44 | | 2.85 | 2.14 | | | | | | | | | | | | | | |
| **17a_2** | 44 | | 1.97 | 2.64 | | | | | | | | | | | | | | |
| **17g** | 31 | | | | | | | | | | | | | | | | 2.23 | |
| **18b_1** | 43 | | | | | | | | | | | | | | 2.62 | 2.21 | | |
| **18b_2** | 43 | | | | | | | | | | | | | | | | | |
| **18c_1** | 15 | 1.87 | | | | | | | | | | | | | | | | |
| **18c_2** | 15 | | | | | | | | | | | | | | | | | |
| **18e** | 15 | | | | | | | | | | | | | | | | | |
| **18g** | 15 | | | | | | | | | | | | | | 2.78 | 2.23 | 2.01 | |
| **Mef_1** | 100 | | | | | | | | | | | | | | | | | 1.84 |
| **Mef_2** | 100 | 2.64 | | | | | | | | | | | | | | | | 1.63 |

[a]concentration 10μM

**Table S2.** Hydrogen bond lengths (Å) between water (bridged to amino acid) and new compounds, related to percentage of inhibition (I%); for all ligands, the different poses (indicated by the underscore sign) and deprotonated form ($^-$) are reported.

| **Ligand** | I (%) | ser59 | ile60 | gly61 | thr62 | pro69 | ser70 | ser73 | trp74 | arg75 | trp85 | glu415 | asn420 | gly421 | cys434 |
|---|---|---|---|---|---|---|---|---|---|---|---|---|---|---|---|
| **5a_1** | 27 | 3 | | | 2.8 | | | | | 1.9 | | | | | |
| **5a_2** | 27 | 3 | | | | | | | | 1.76 | | | | | |
| **5a_3** | 27 | 2.7 | | | | | | | | 2 | | | | | |
| **5a_1$^-$** | 27 | | | | | | | | | 2.2 | | | | | |
| **5a_2$^-$** | 27 | | | | | | | | | 2 | | | | | |
| **5a_3$^-$** | 27 | | | | 2.7 | | | | | 2.2 | | | | | |
| **5b_1** | 49 | | | | | 2.7 | 2.8 | | | | | | | | |
| **5b_2** | 49 | | | | | | | | | | | | | | |
| **5b_1$^-$** | 49 | | | | | | | | | 2.5 | 2.5 | | | | |
| **5b_2$^-$** | 49 | | | | | | | | | 2.5 | 2.6 | | | | |
| **7_1** | 80 | | | | | | | | | | | | | | |
| **7_2** | 80 | | | | | | | | | | | | | | |
| **7$^-$** | 80 | | | | | | | | 2 | | | | | | |
| **12a_1** | 55 | | | 2.2 | | | | | | | | | | | |
| **12a_2** | 55 | | | | | | | | | 2.4 | | | | | |
| **12a_3** | 55 | | | | | | | | 2.6 | | | | | | |
| **12a_1$^-$** | 55 | | | | | | | | | | | | | | |
| **12a_2$^-$** | 55 | | | | | | | | | 1.9 | | | | | |
| **12b** | 74 | | | | | | | | | | | | | | |
| **12b$^-$** | 74 | | | | | | | | | | | | | | |
| **12c_1** | 50 | | | | | | | | | | | | | | |
| **12c_2** | 50 | | | | | | | | | 2.1 | | | | | |
| **12c_1$^-$** | 50 | | | | | | | | | | | | | | |
| **12c_2$^-$** | 50 | | | | | | | | 2.7 | 2 | | | | | |
| **12d** | 11 | | | | | | 2.64 | 2.5 | 2.43 | | | | | | |
| **12d$^-$** | 11 | | | | | | | | | | | | | | |
| **12e** | 85 | | | | | | | | | | | | | | |
| **12e$^-$** | 85 | | | | | | | | 3 | | | | | | |
| **12f_1** | 80[a] | | | | | | | | | | | | | | |
| **12f_2** | 80[a] | | | | | | 2.96 | | | | | | | | |
| **12f$^-$** | 80[a] | | | | | | | | 1.64 | | | | | | |
| **12g** | 57 | | | | | | | | | 2.2 | | | | | |
| **12g$^-$** | 57 | | | | | | | | 2.1 | 2.2 | | | | | |
| **17a_1** | 44 | | | | | | | | | | | | | | 2.19 |
| **17a_2** | 44 | 2.76 | | 2.61 | 2.6 | | | | | 2.36 | | | | | |
| **17** | 31 | | | | | | | | | | | 2.05 | 2.14 | | |
| **18b_1** | 43 | 2.66 | | | 2.23 | | | | | | | | | | |
| **18b_2** | 43 | | | | | | | | | | | | | | |
| **18c_1** | 15 | | 1.87 | | | | | | | | | | | | |
| **18c_2** | 15 | 2.68 | | 2.47 | 2.57 | | | | | | | | | | |
| **18e** | 15 | | | | | | | | 1.97 | | | | | | |
| **18g** | 15 | | | | | | | | | | | | | 2.88 | |
| **Mef_1** | 100 | 1.96 | | | | | | | | | | | | | 1.77 |
| **Mef_2** | 100 | | | | | | | | | | | | | | |

[a]concentration 10µM

4. **Table S3. The actual and predicted values of I% and the absolute errors.**

| Samples | Compds N° | Actual | Predicted | Error |
|---|---|---|---|---|
| Mef_1 | **Mef_1** | 100 | 97 | -3 |
| Mef_2 | **Mef_2** | 100 | 102.8 | 2.8 |
| RF2 | **12b** | 74 | 63.8 | -10.2 |
| RF2- | **12b**$^{-}$ | 74 | 80.2 | 6.2 |
| RF4 | **12e** | 85 | 71.9 | -13.1 |
| RF4- | **12e**$^{-}$ | 85 | 58 | -27 |
| RF6_1 | **12f_1** | 80 | 61.4 | -18.6 |
| RF6_2 | **12f_2** | 80 | 65.9 | -14.1 |
| RF6- | **12f**$^{-}$ | 80 | 74.4 | -5.6 |
| M1 | **17g** | 31 | 37.5 | 6.5 |
| M10_1 | **18b_1** | 43 | 37.5 | -5.5 |
| M10_2 | **18b_2** | 43 | 37.5 | -5.5 |
| M12_1 | **17a_1** | 44 | 37.5 | -6.5 |
| M12_2 | **17a_2** | 44 | 22.5 | -21.5 |
| M4 | **18g** | 15 | 37.5 | 22.5 |
| M8_1 | **18c_1** | 15 | 15 | 0 |
| M8_2 | **18c_2** | 15 | 37.5 | 22.5 |
| M9 | **18e** | 15 | 37.5 | 22.5 |
| RF3 | **12d** | 11 | 11 | 0 |
| RF3- | **12d**$^{-}$ | 11 | 37.5 | 26.5 |
| RF1_1 | **12a_1** | 55 | 48.1 | -6.9 |
| RF1_2 | **12a_2** | 55 | 35.8 | -19.2 |
| RF1_3 | **12a_3** | 55 | 73.3 | 18.3 |
| RF1_1- | **12a_1**$^{-}$ | 55 | 71.7 | 16.7 |
| RF1_2- | **12a_2**$^{-}$ | 55 | 59.4 | 4.4 |
| RF5_1 | **12c_1** | 50 | 37.5 | -12.5 |
| RF5_2 | **12c_2** | 50 | 59.8 | 9.8 |
| RF5_1- | **12c_1**$^{-}$ | 50 | 65.9 | 15.9 |
| RF5_2- | **12c_2**$^{-}$ | 50 | 58.5 | 8.5 |
| RF7_1 | **5b_1** | 49 | 37.5 | -11.5 |
| RF7_2 | **5b_2** | 49 | 49 | 0 |
| RF7_1- | **5b_1**$^{-}$ | 49 | 49.4 | 0.4 |
| RF7_2- | **5b_2**$^{-}$ | 49 | 48.5 | -0.5 |
| RF8 | **12g** | 57 | 50.9 | -6.1 |
| RF8- | **12g**$^{-}$ | 57 | 63.7 | 6.7 |
| RF9_1 | **5a_1** | 27 | 25.3 | -1.7 |
| RF9_2 | **5a_2** | 27 | 23.6 | -3.4 |
| RF9_3 | **5a_3** | 27 | 29.4 | 2.4 |
| RF9_1- | **5a_1**$^{-}$ | 27 | 33.9 | 6.9 |
| RF9_2- | **5a_2**$^{-}$ | 27 | 26.8 | -0.2 |
| RF9_3- | **5a_3**$^{-}$ | 27 | 34.9 | 7.9 |
| RF10_1 | **7_1** | 80 | 76.4 | -3.6 |
| RF10_2 | **7_2** | 80 | 68.7 | -11.3 |
| RF10- | **7**$^{-}$ | 80 | 80 | 0 |

## 5. Docking images of more representative compounds (7, 12e and 12f)

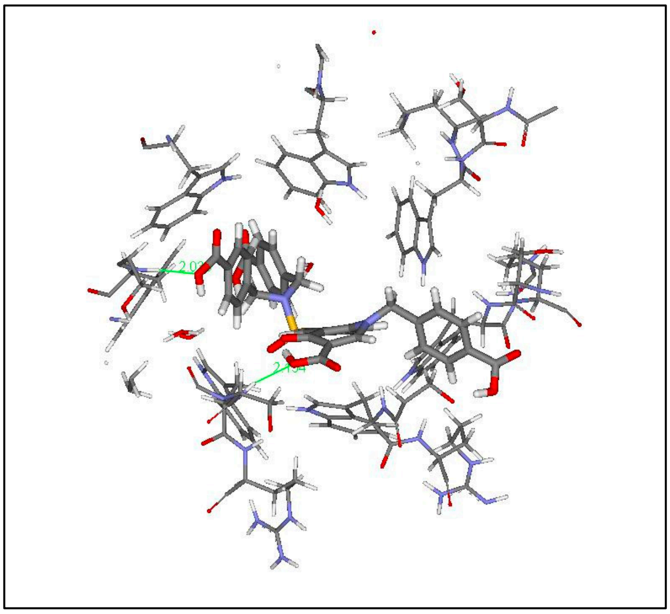

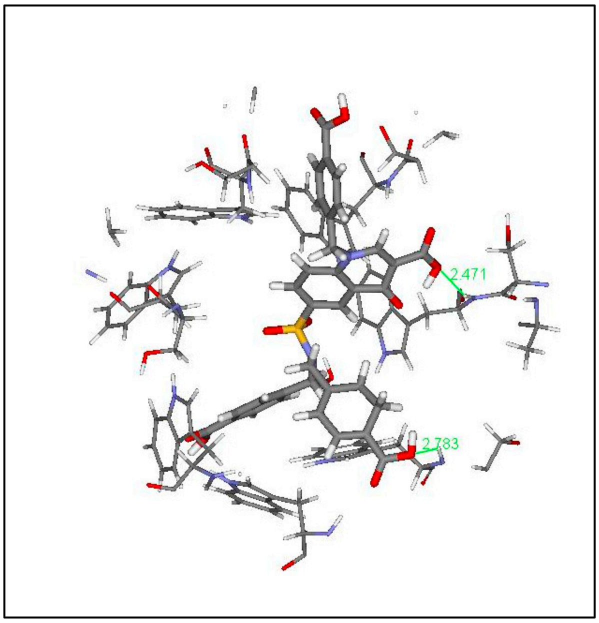


**Fig. S2.** Compound 7_1 Compound 7_2

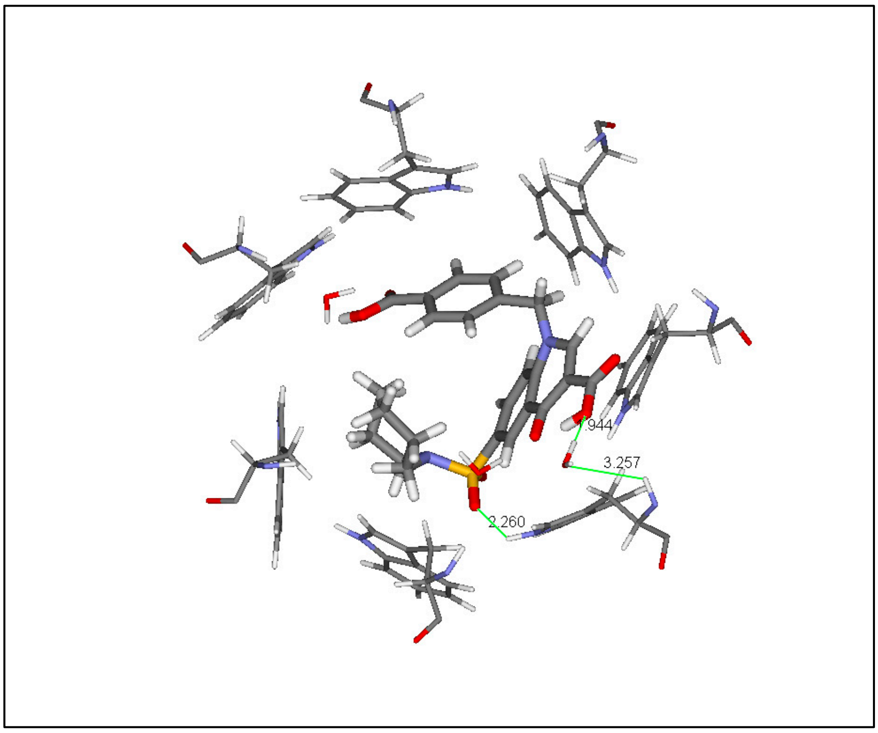


**Fig. S3**. Compound 12e

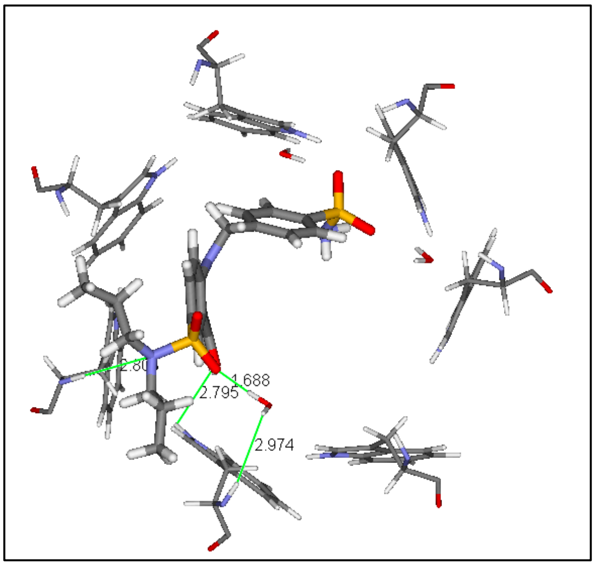


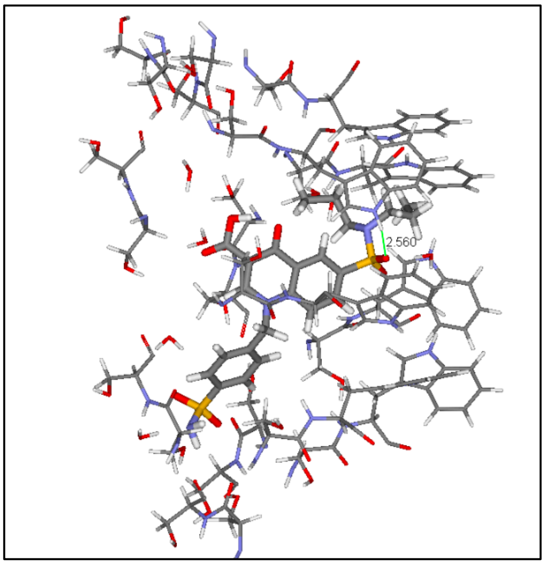


**Fig. S4.** Compound 12f_1 Compound 12f_2

# 6. NMR spectra of some representative compounds

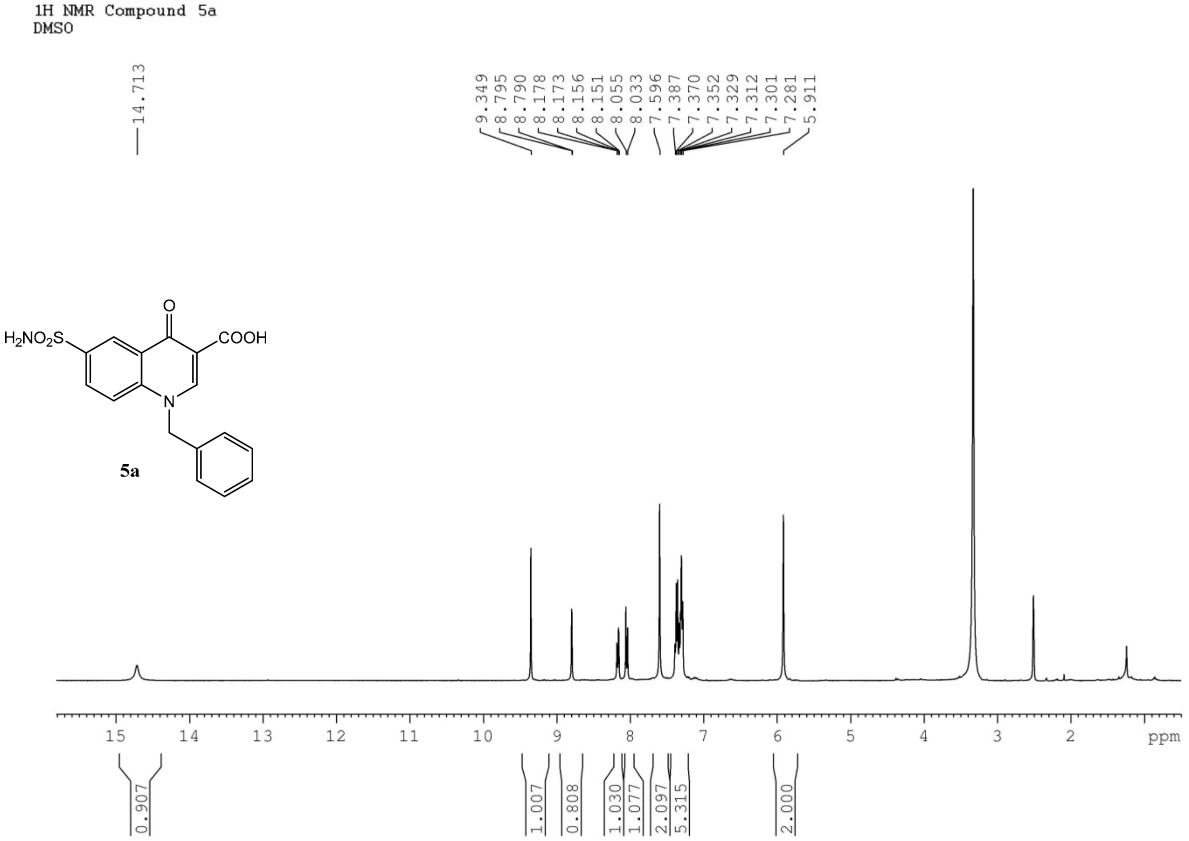


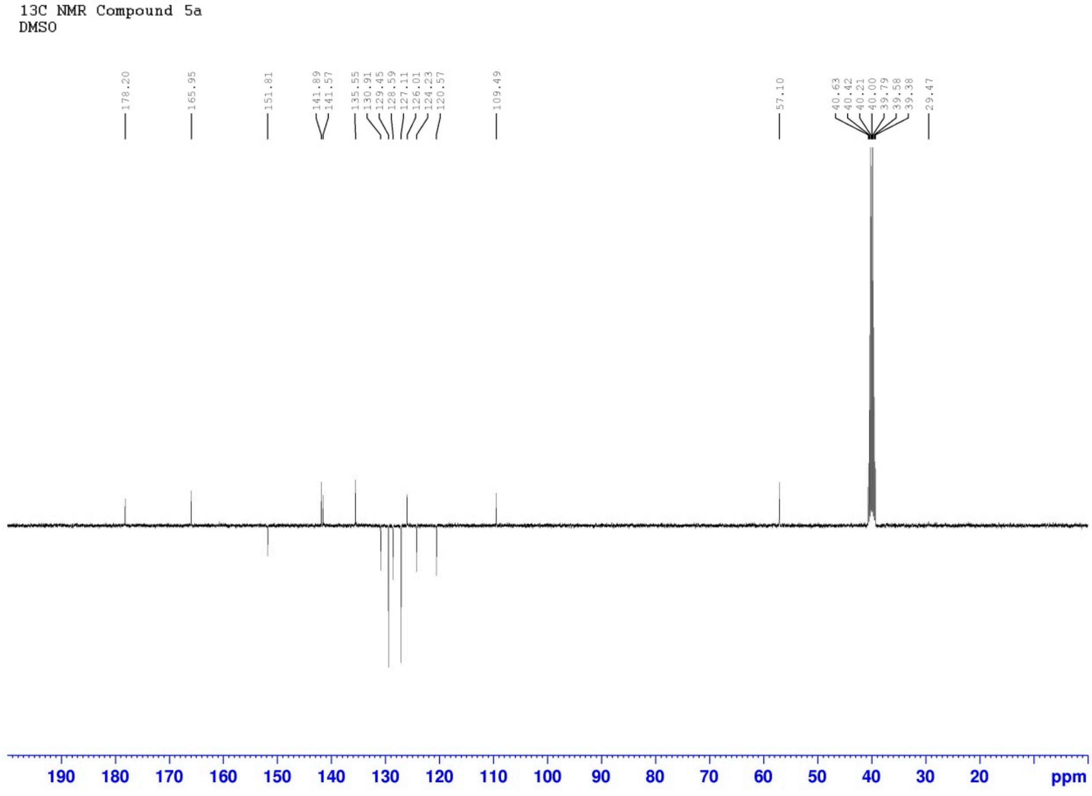

1H NMR Compound 5b
DMSO

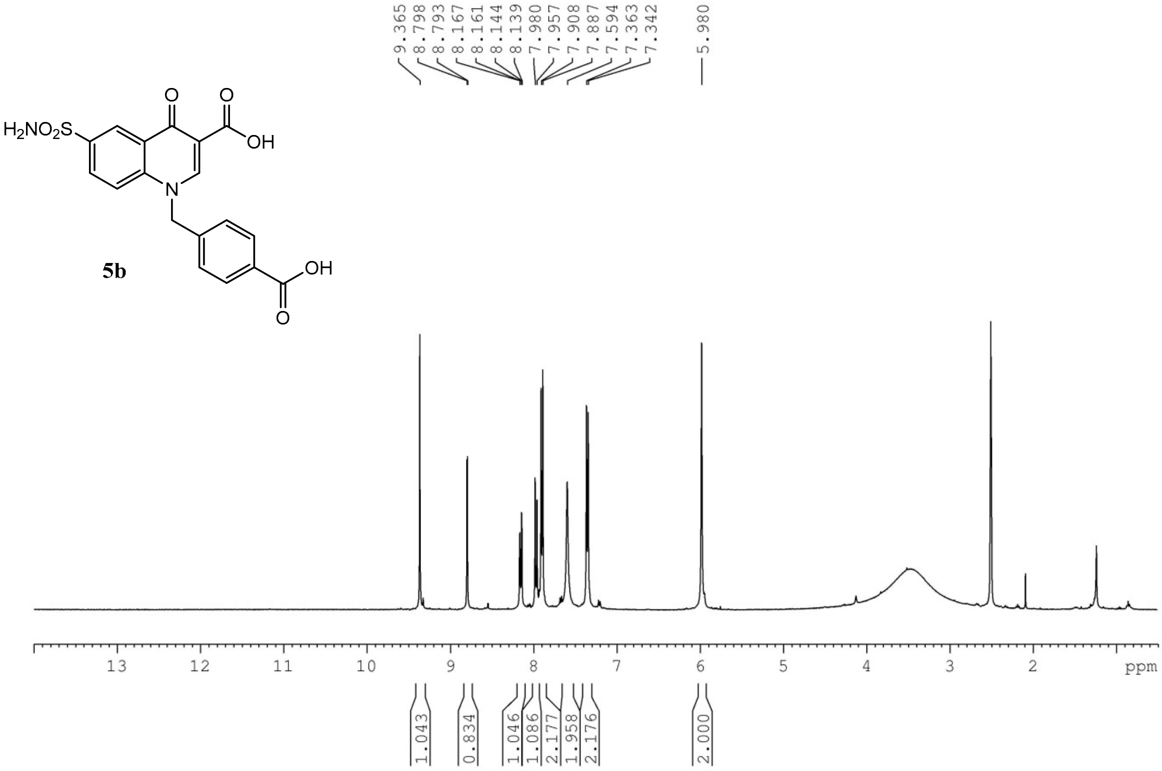


13C NMR Compound 5b
DMSO

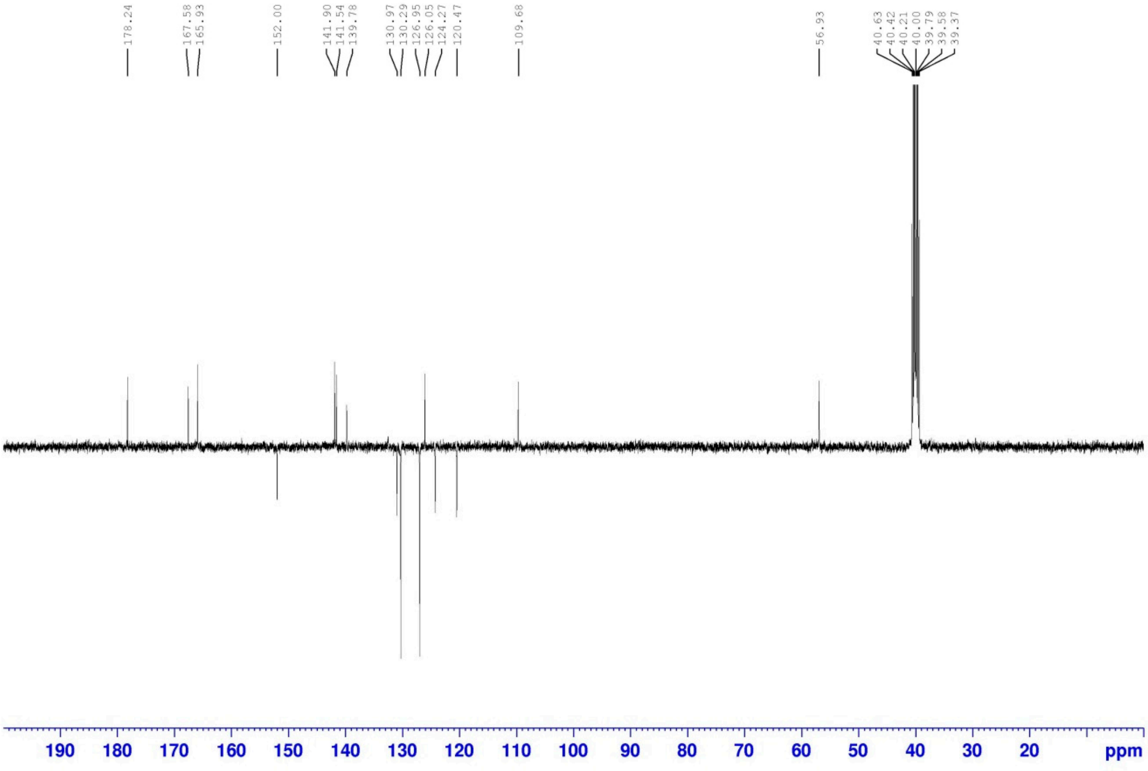

1H NMR Compound 12a
DMSO

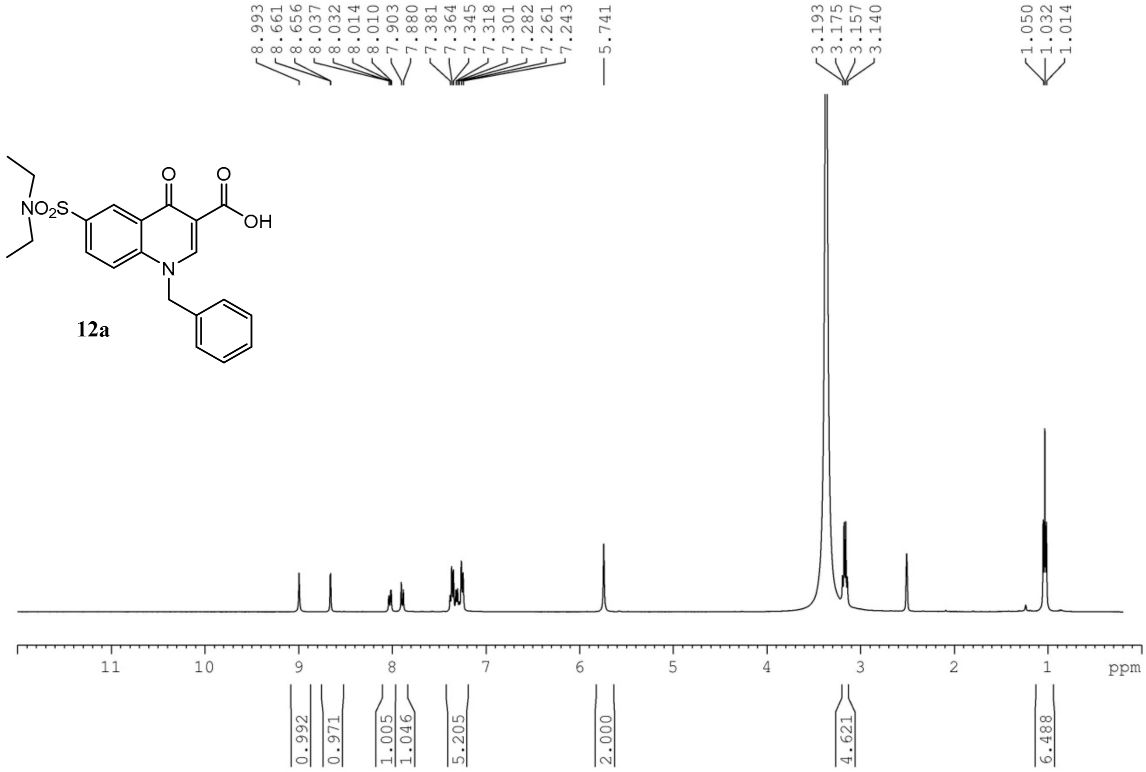


12a

13C NMR Compound 12a
DMSO

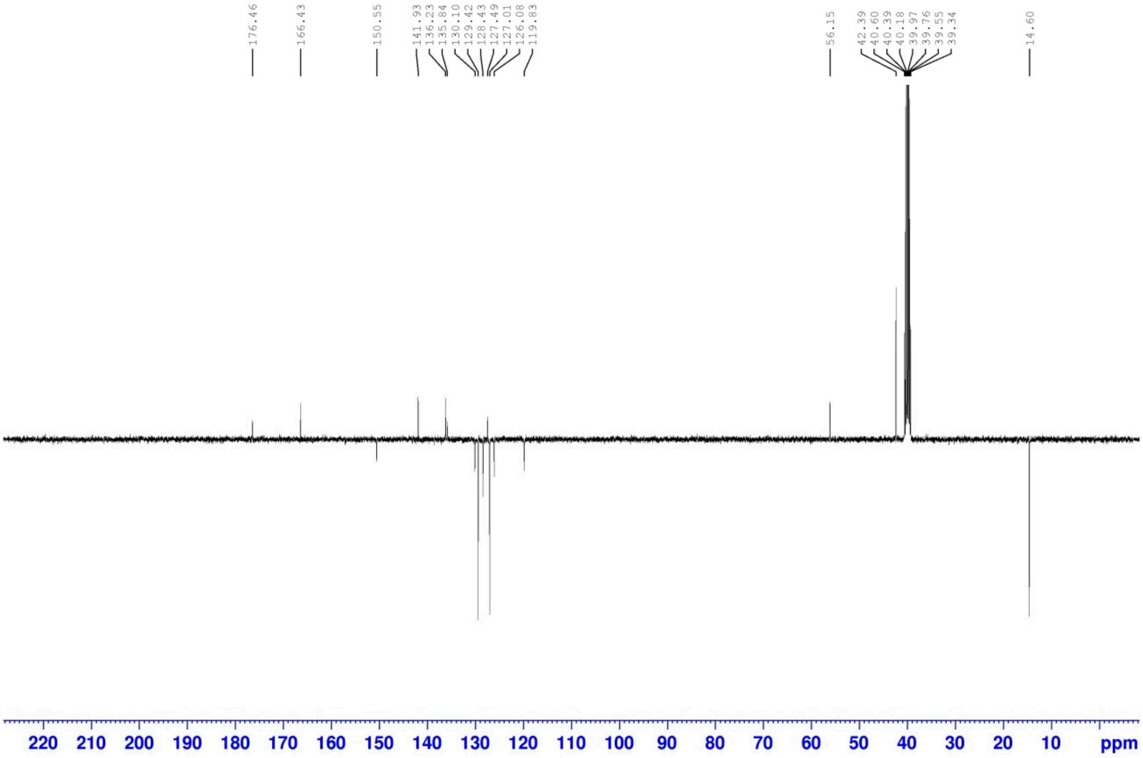

1H NMR Compound 12b
DMSO

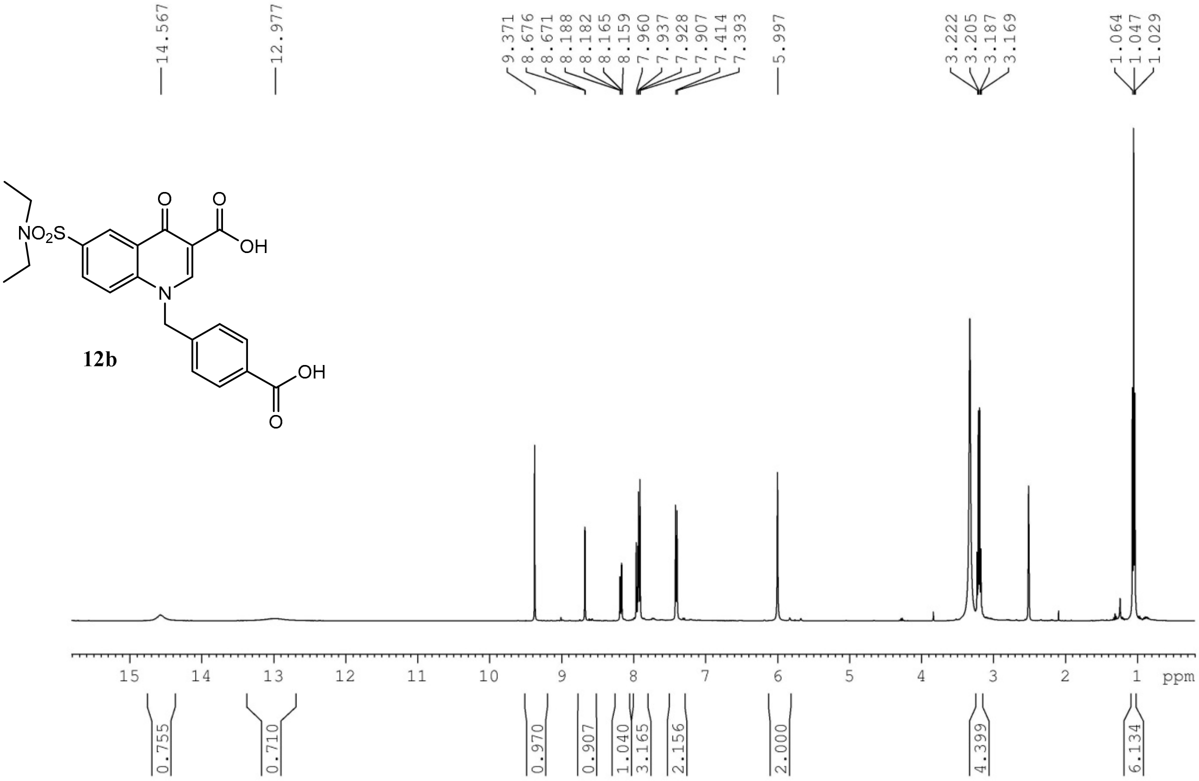


13C NMR Compound 12b
DMSO

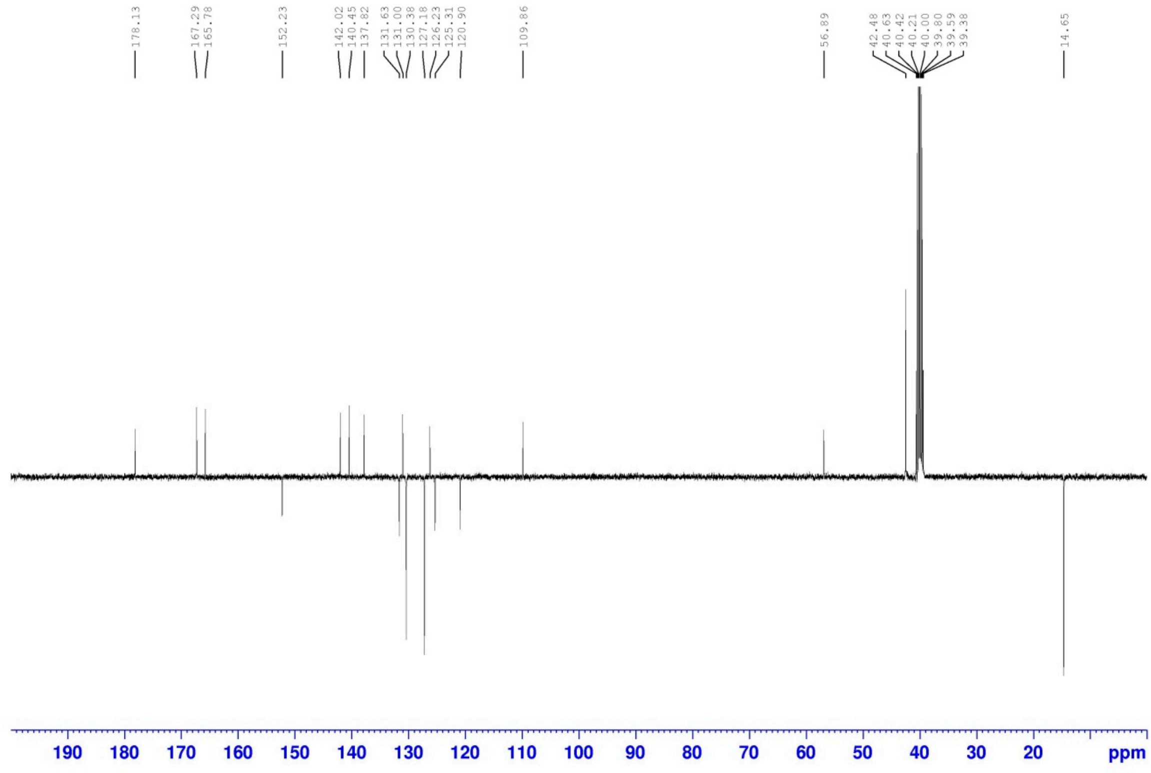

1H NMR Compound 12c
DMSO

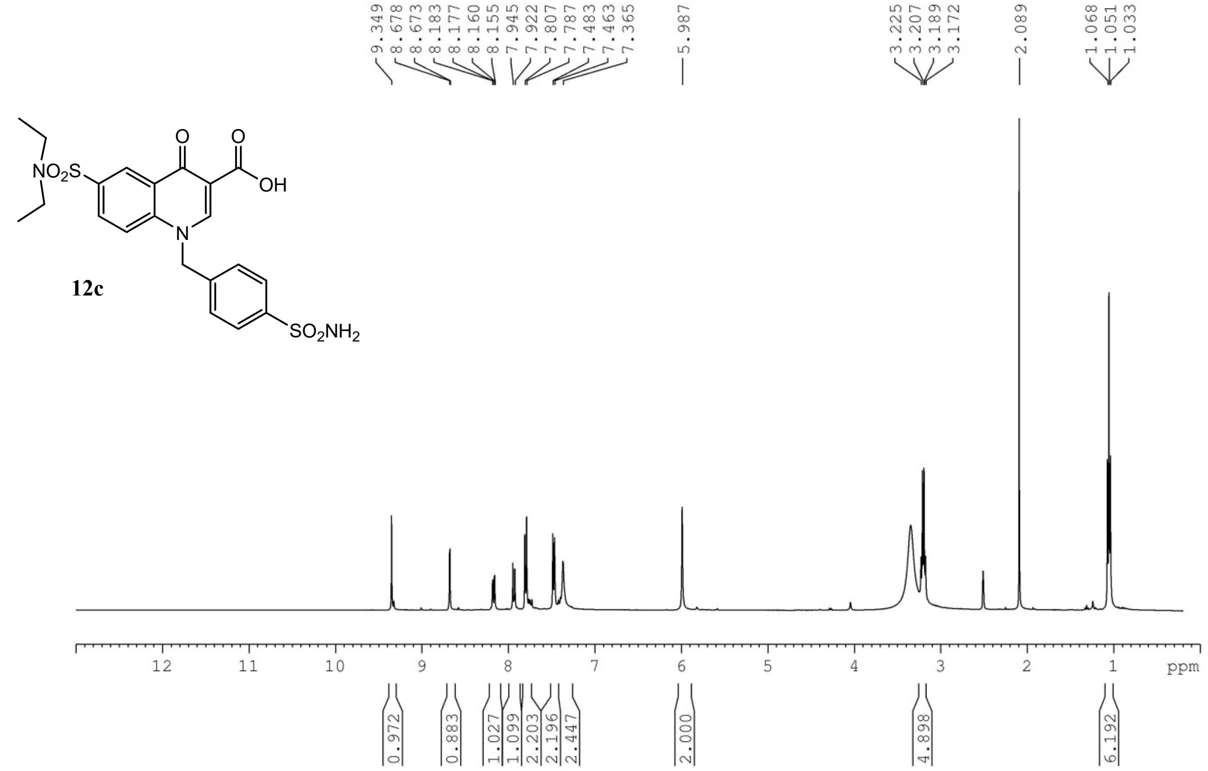


**12c**

13C NMR Compound 12c
DMSO

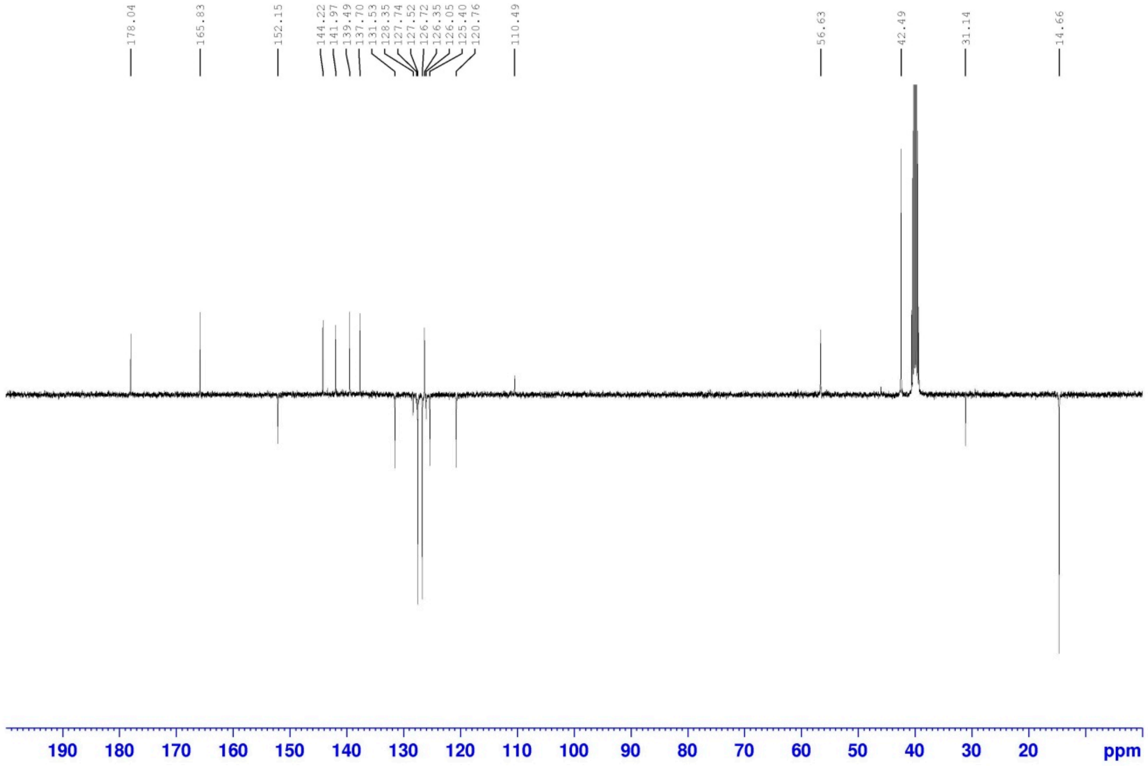

1H NMR Compound 12f
DMSO

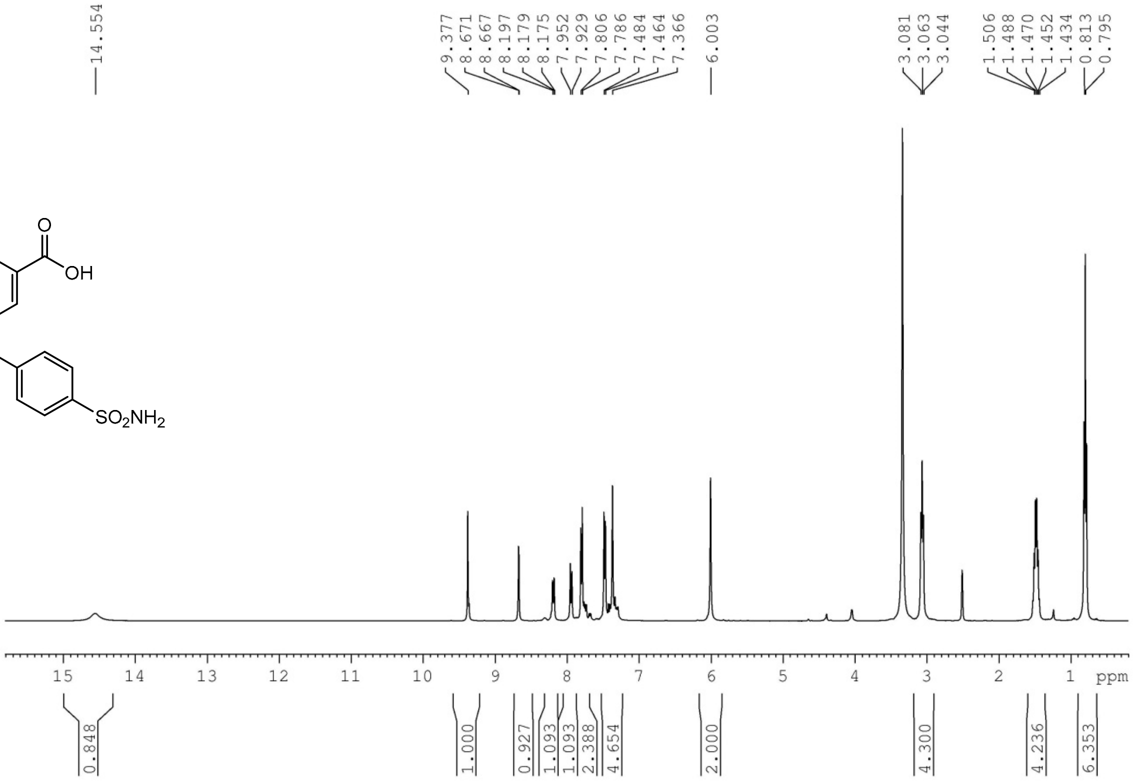


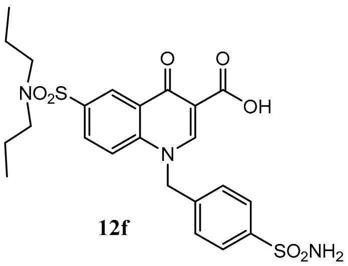


**12f**

13C NMR Compound 12f
DMSO

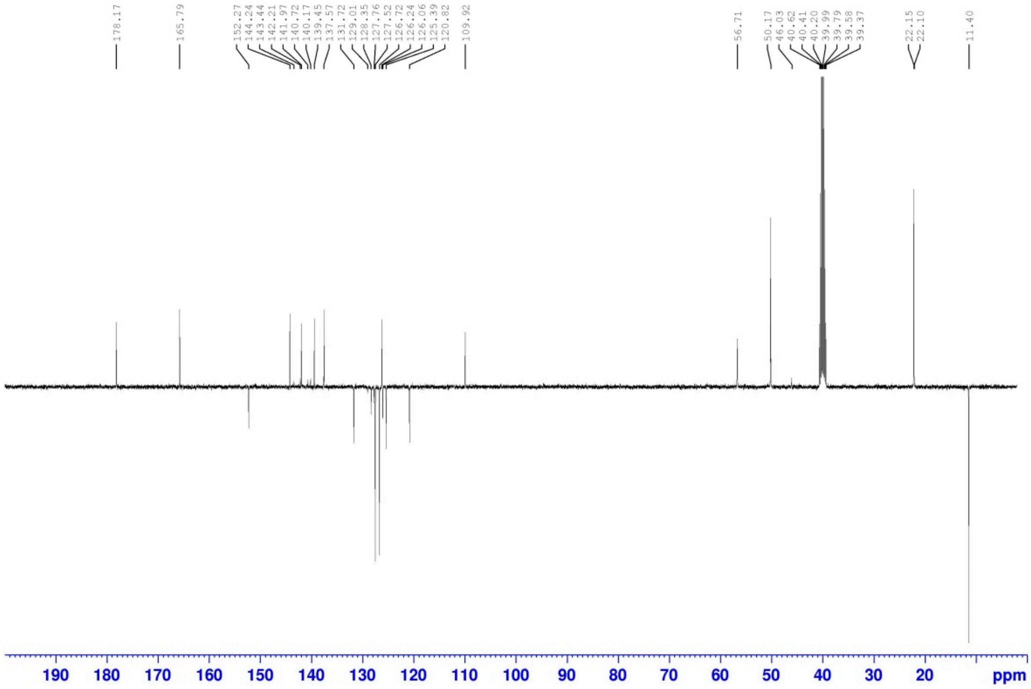

1H NMR Compound 15
CDCl3

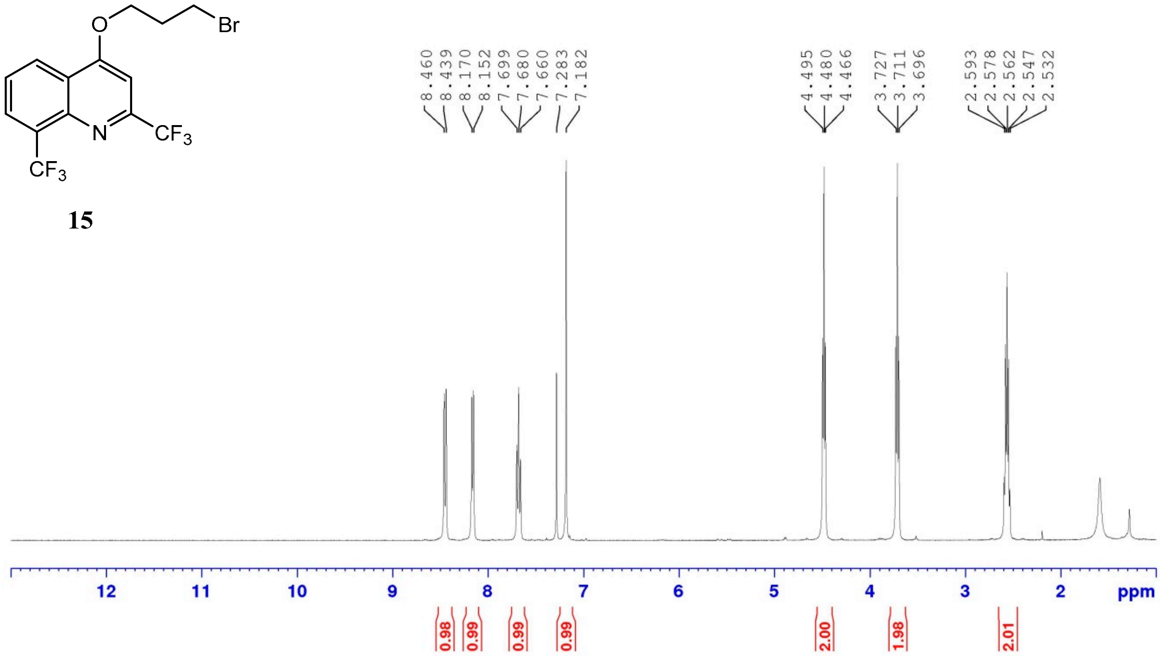


**15**

HMBC Compound 15

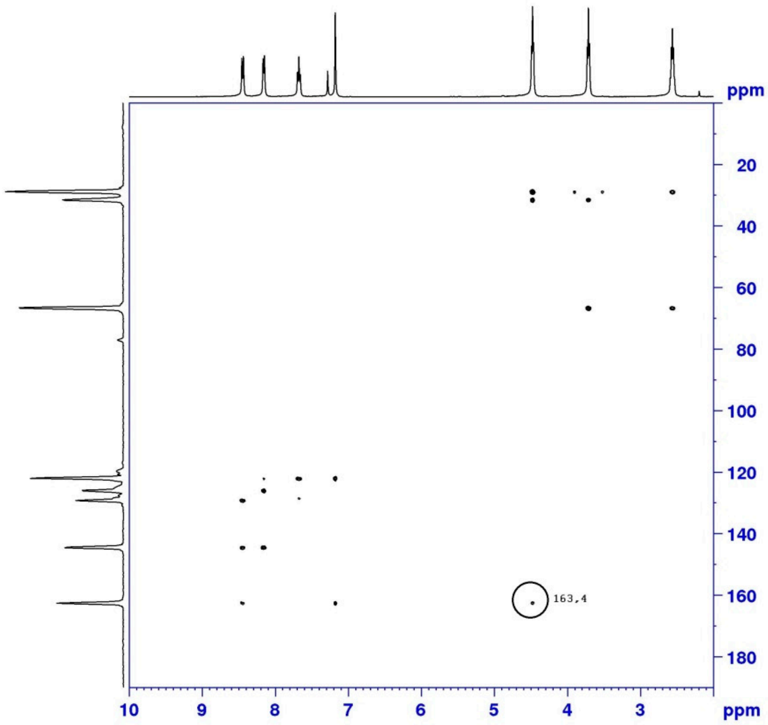

HSQC Compound 15

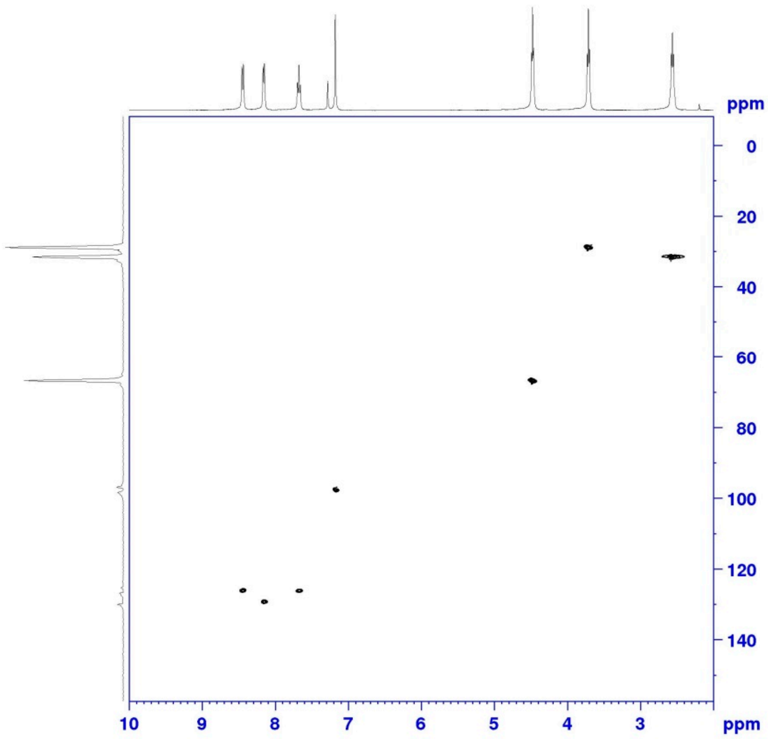


1H NMR Compound 17b
CDCl3

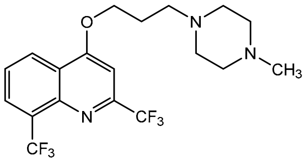


**17b**

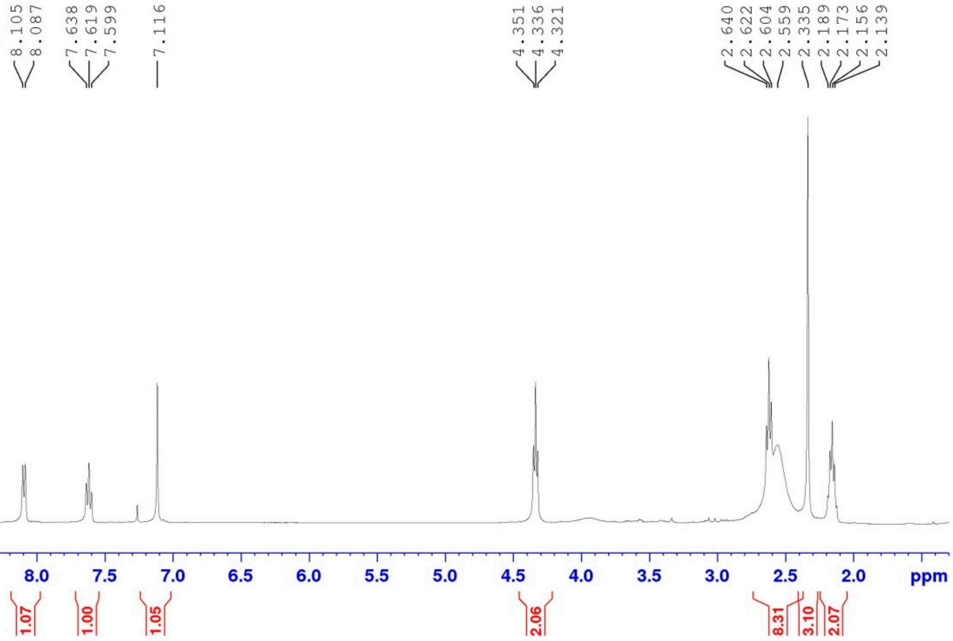

13C NMR Compound 17b
CDCl3

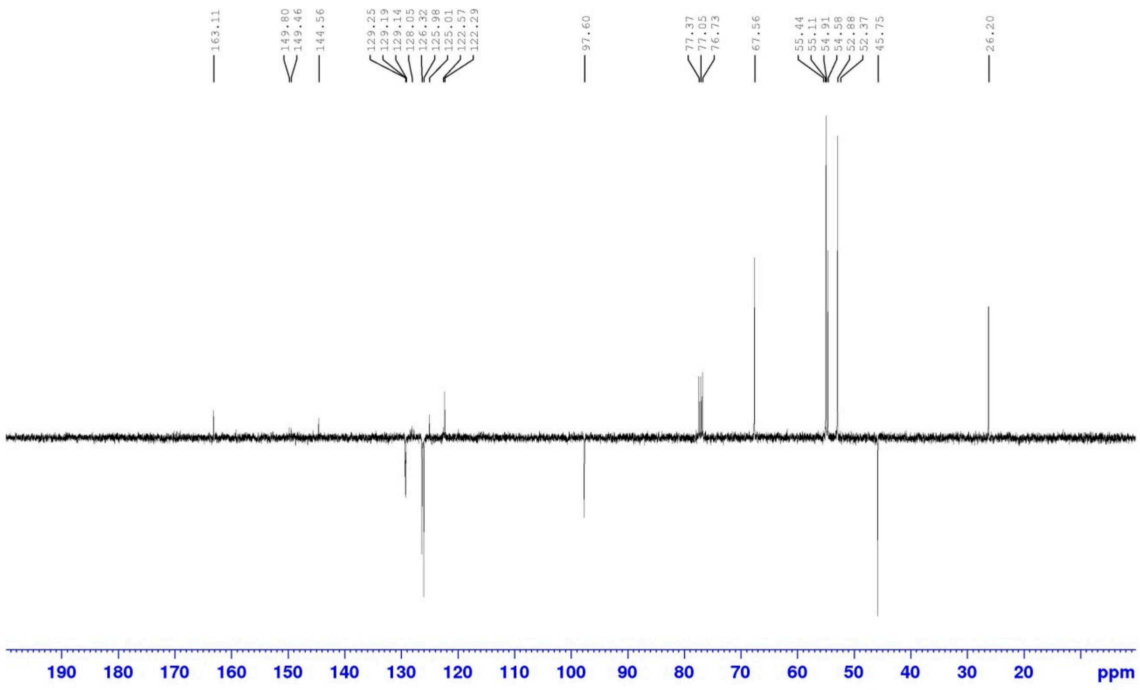


1H NMR Compound 17c
CDCl3

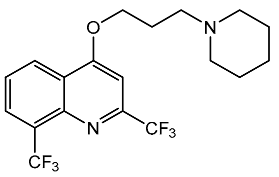


17c

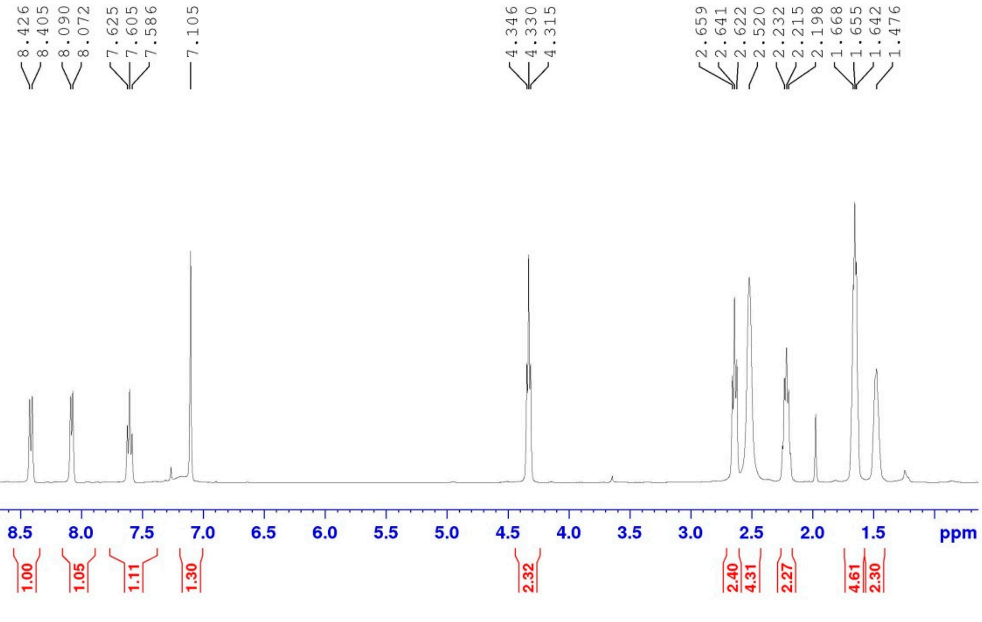

13C NMR Compound 17c
CDCl3

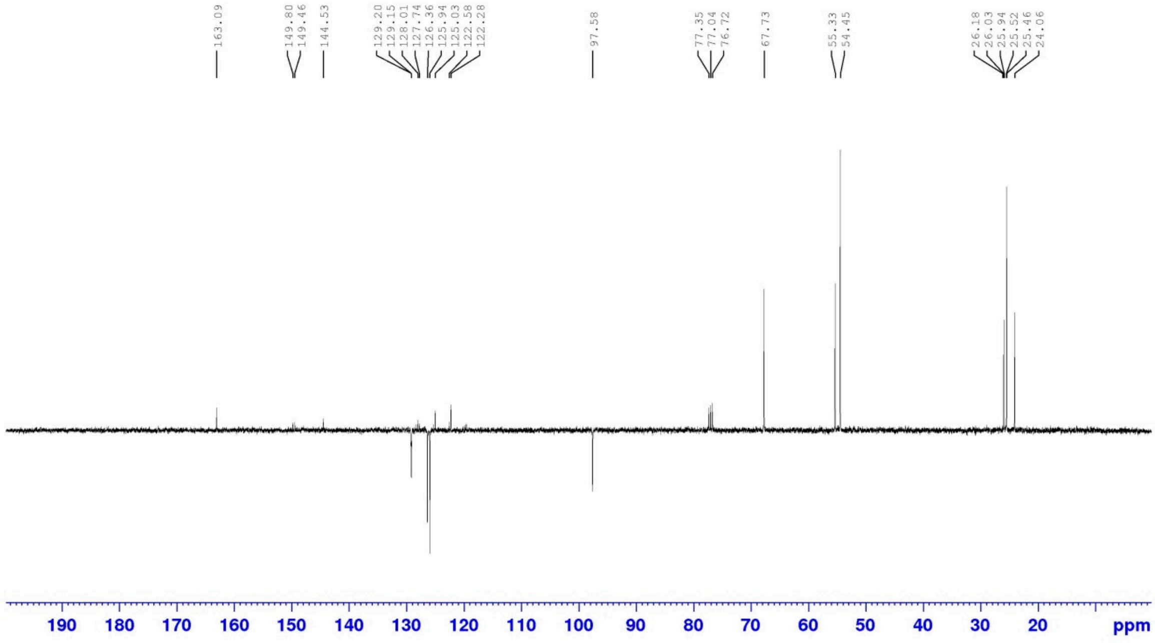


1H NMR Compound 17d
CDCl3

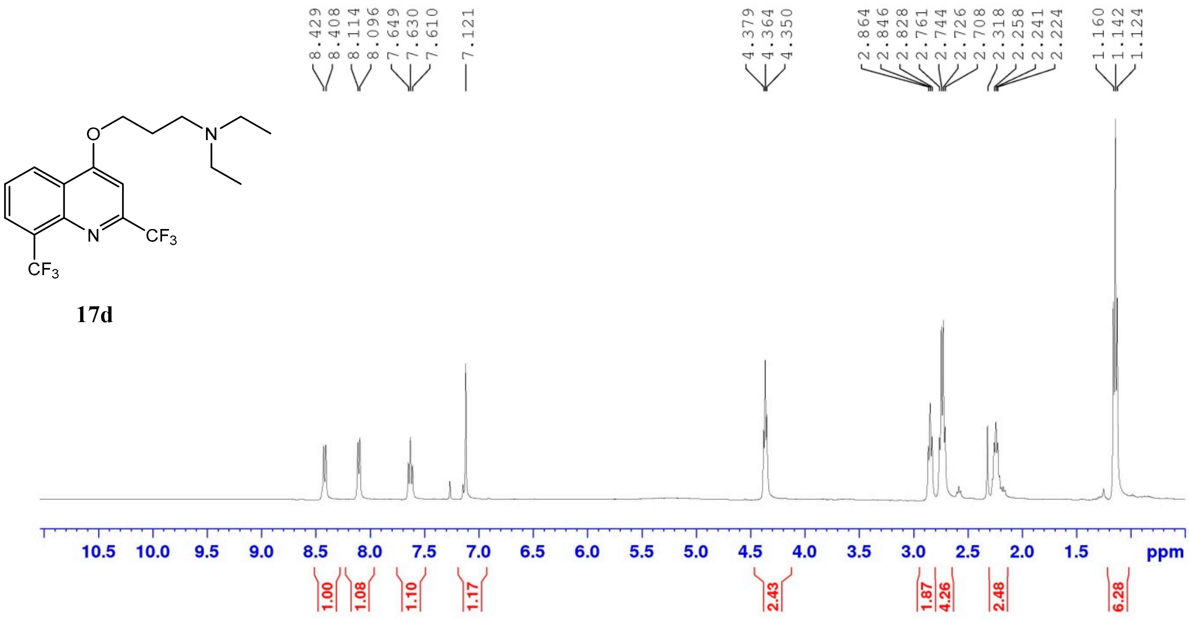

13C NMR Compound 17d
CDCl3

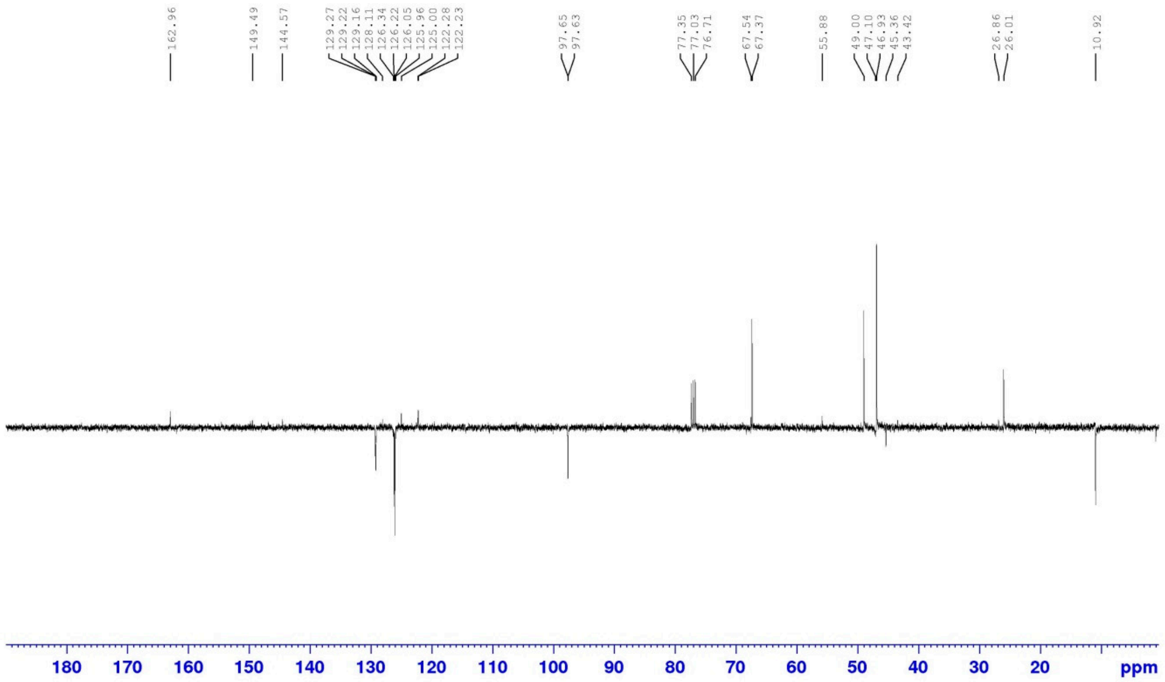


1H NMR Compound 20b
CDCl3

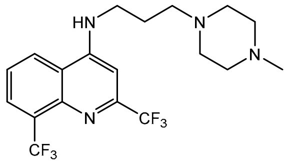


20b

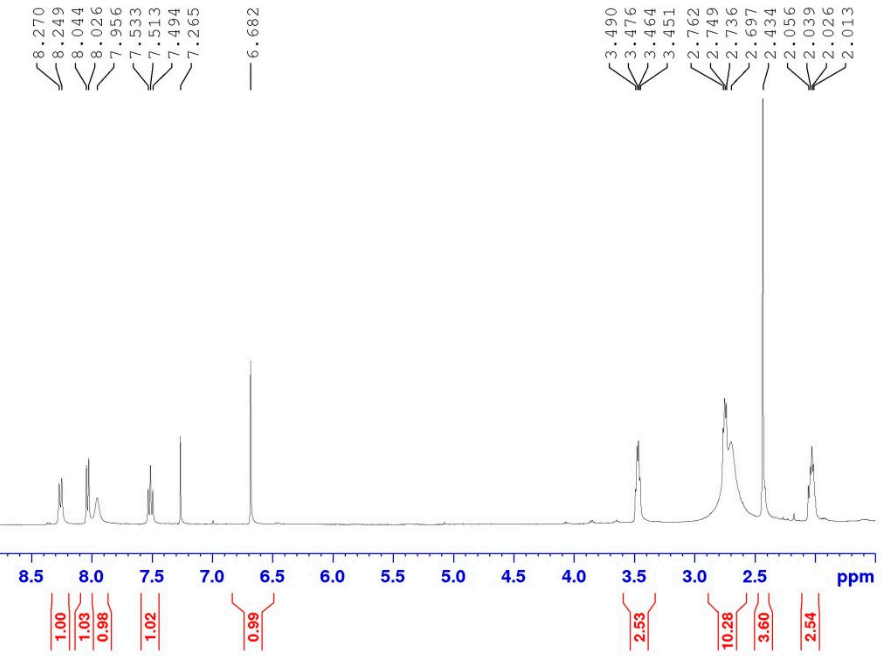

13C NMR Compound **20b**
CDCl3

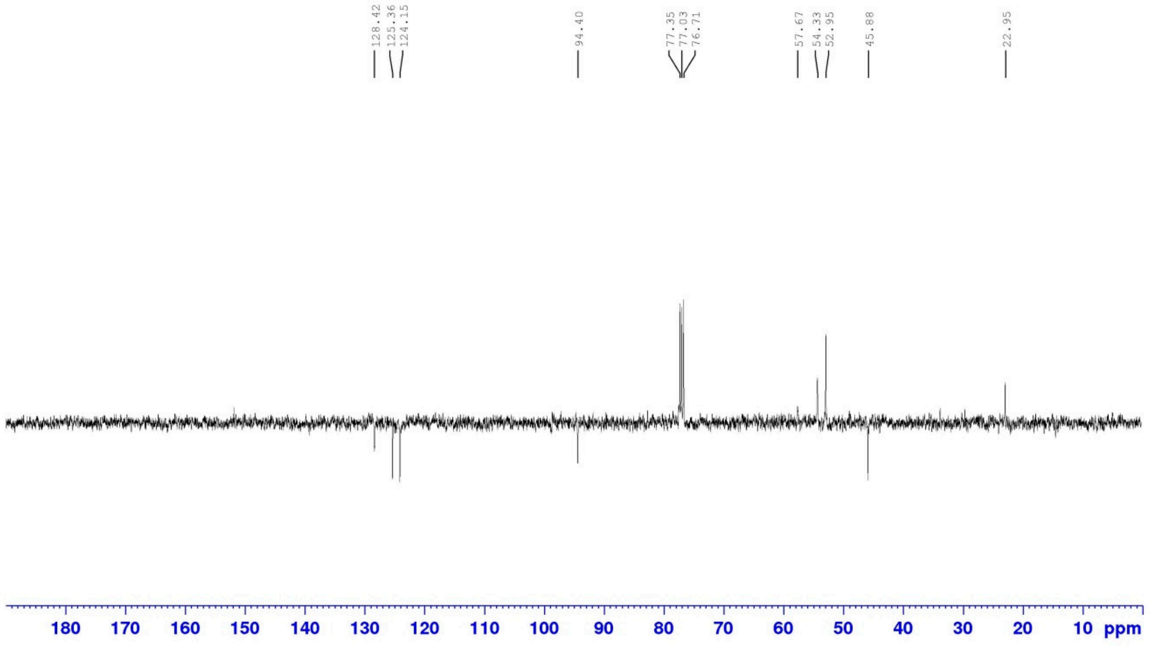


1H NMR Compound **20c**
CDCl3

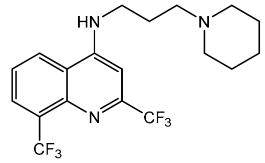


**20c**

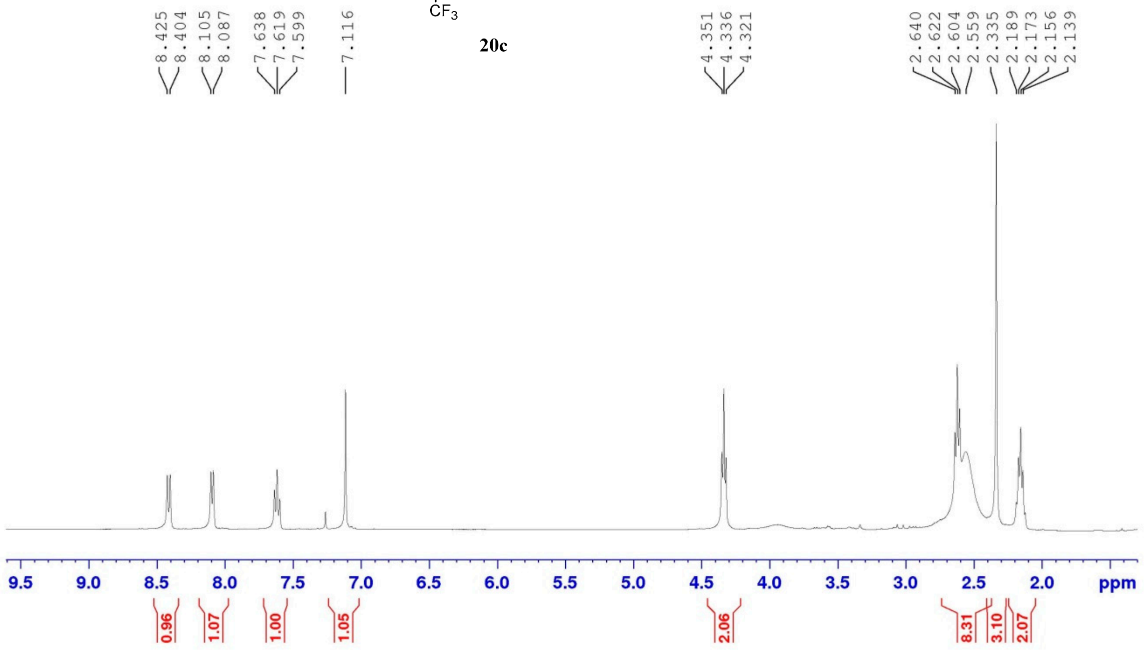

13C NMR Comnpound **20c**
CDCl3

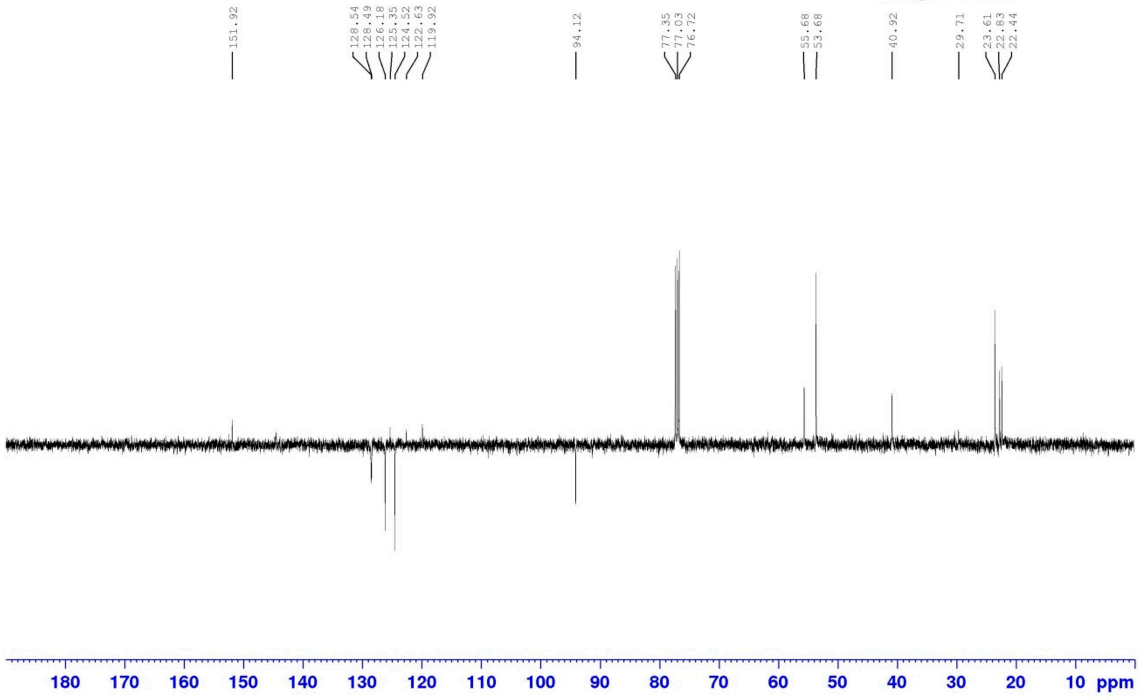


1H NMR Compound **16**
CDCl3

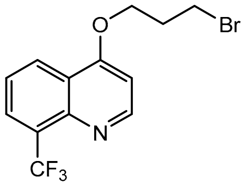


**16**

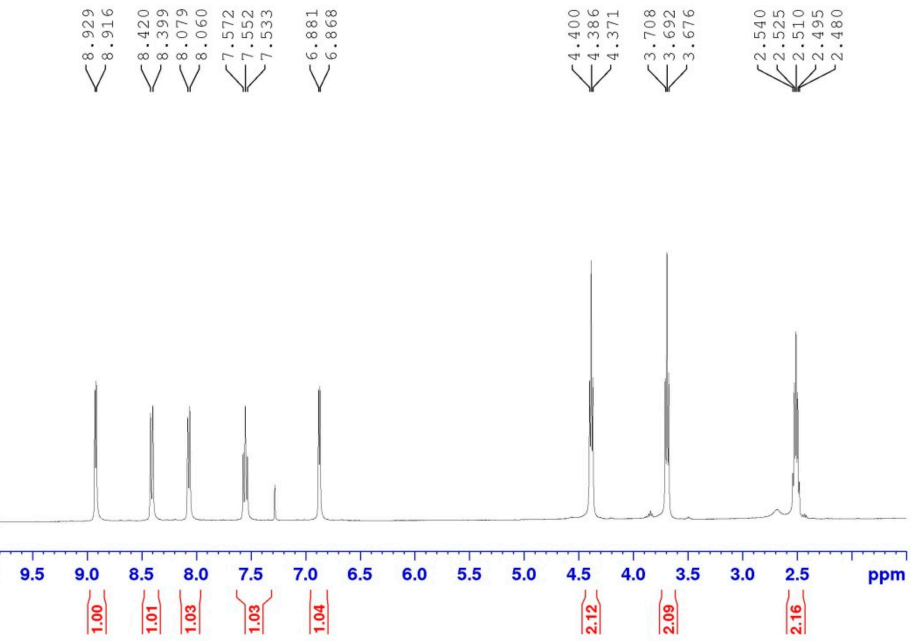

HMBC Compound 16

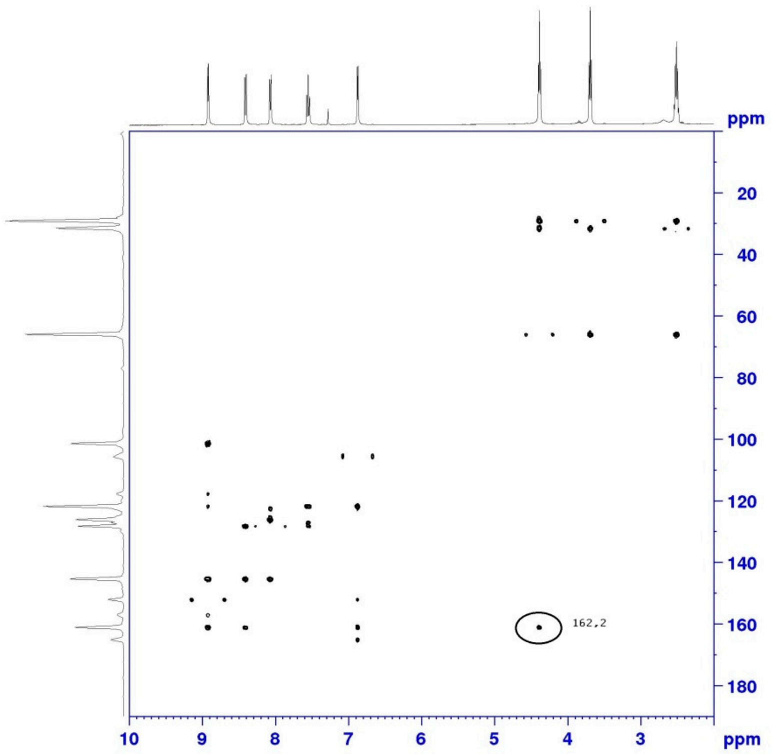


HSQC Compound 16

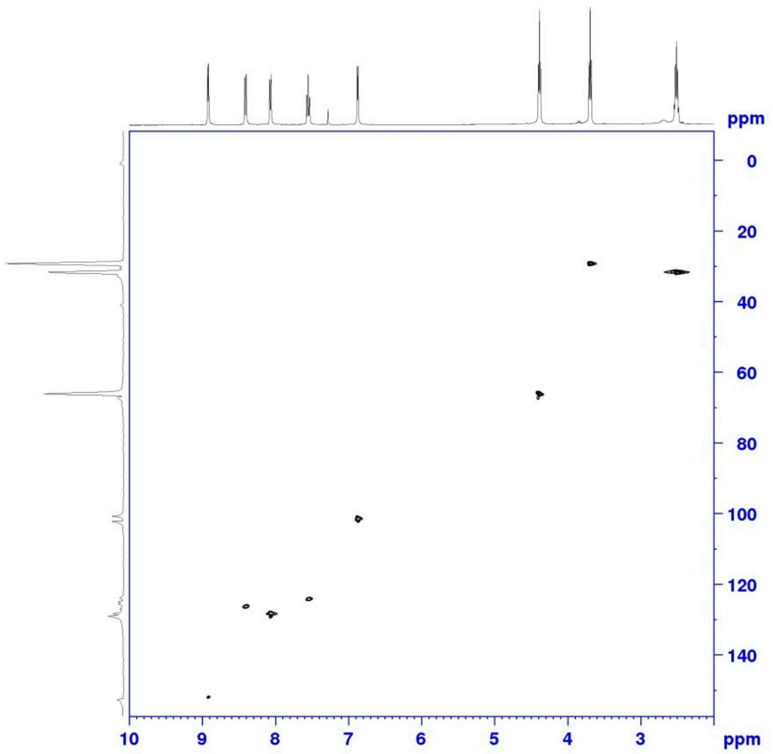

1H NMR Compound 18b
CDCl3

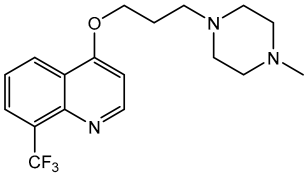


**18b**

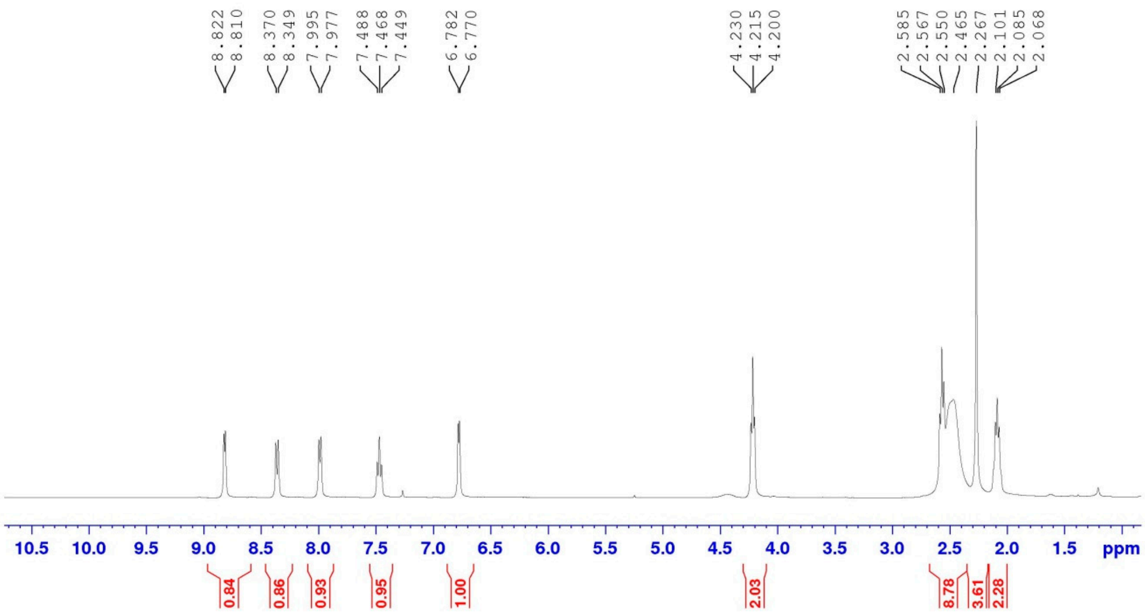


13C NMR Compound 18b
CDCl3

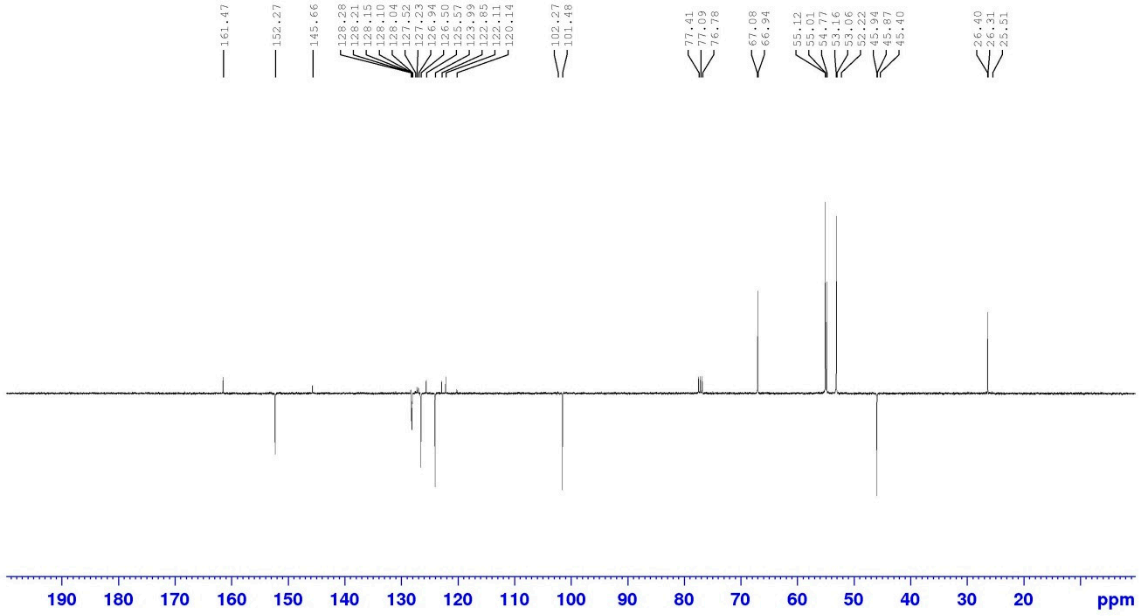

1H NMR Compound 18c
CDCl3

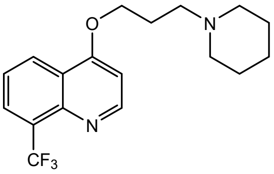


**18c**

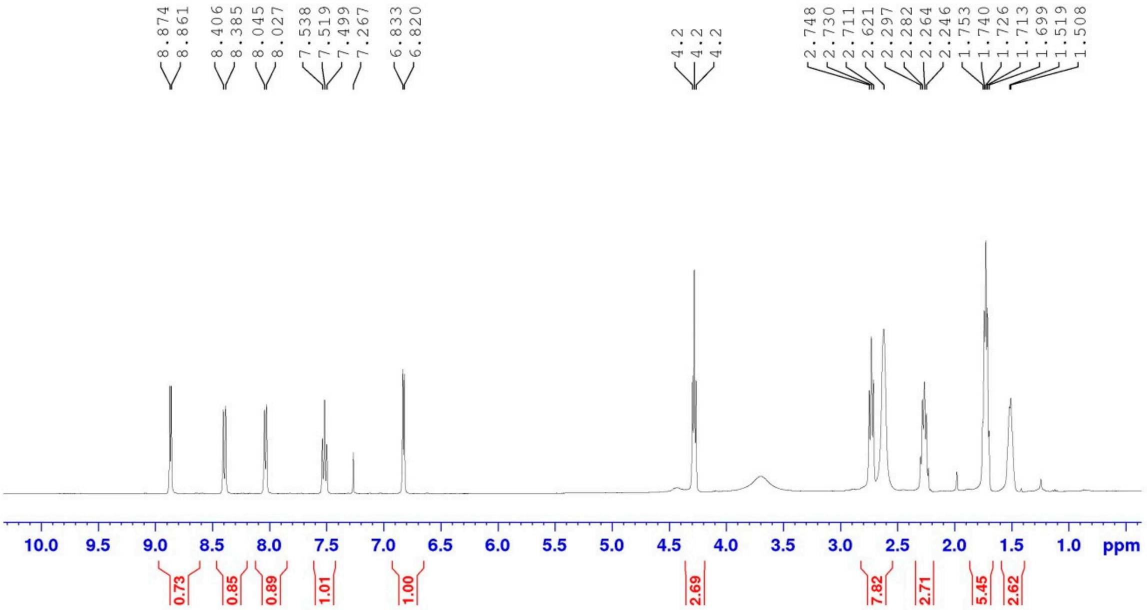


13C NMR Compound 18c
CDCl3

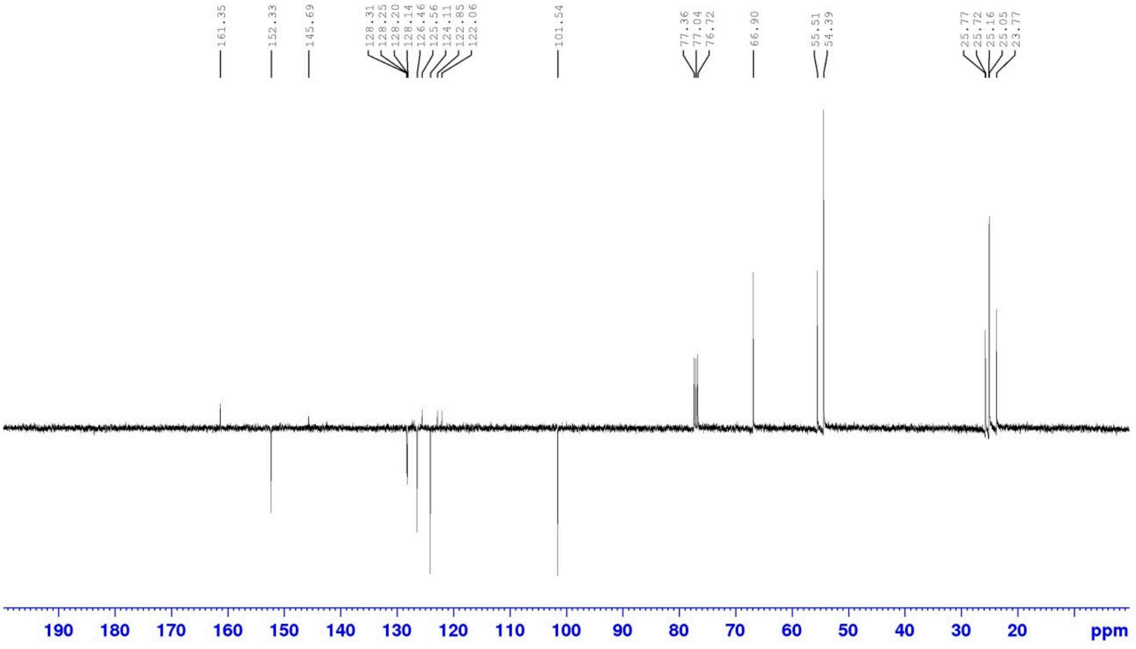

1H NMR Compound 18d
CDCl3

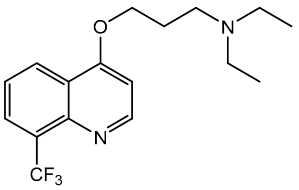


18d

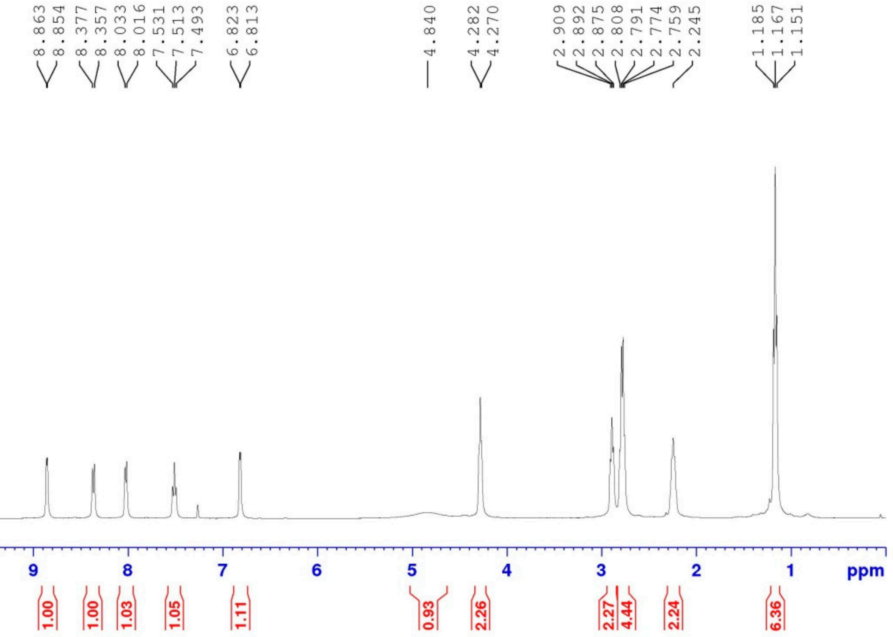


13C NMR Compound 18d
CDCl3

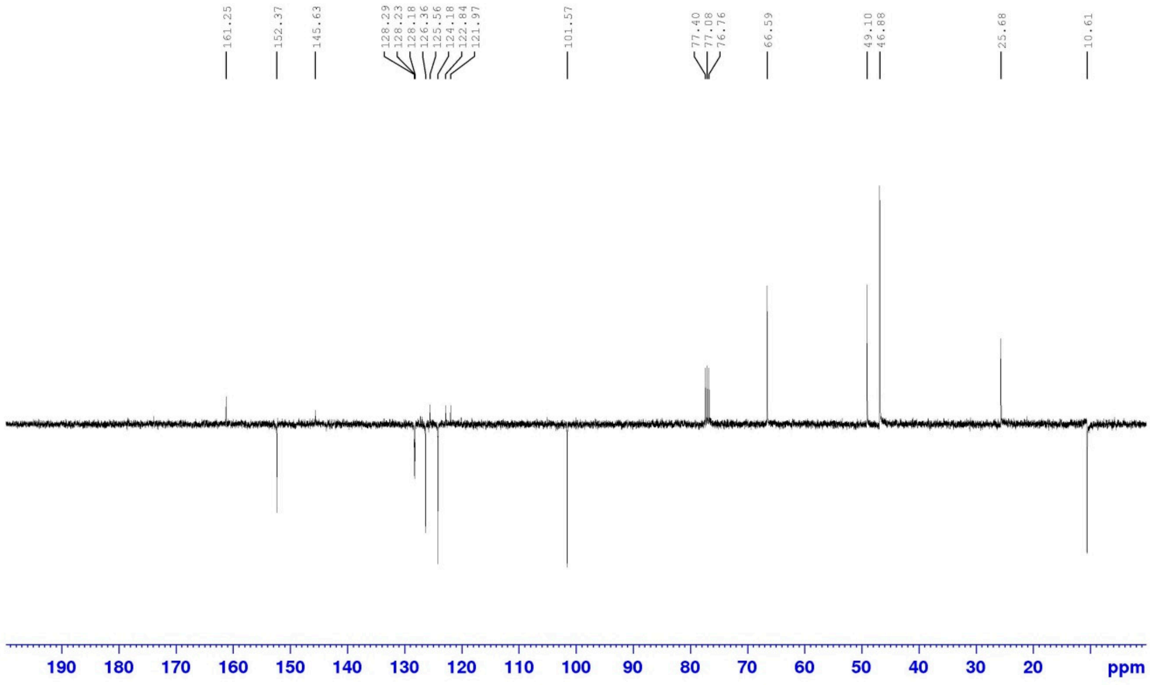

1H NMR Compound 18e
CDCl3

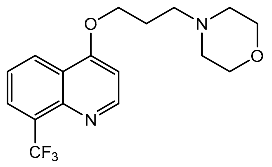

**18e**

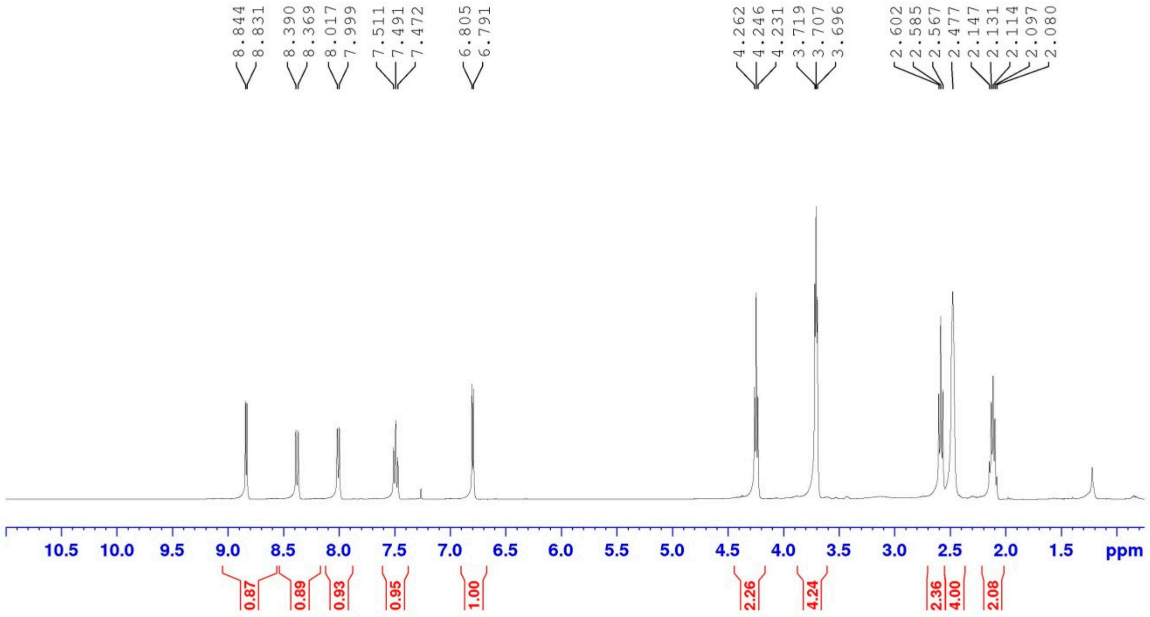


13C NMR Compound 18e
CDCl3

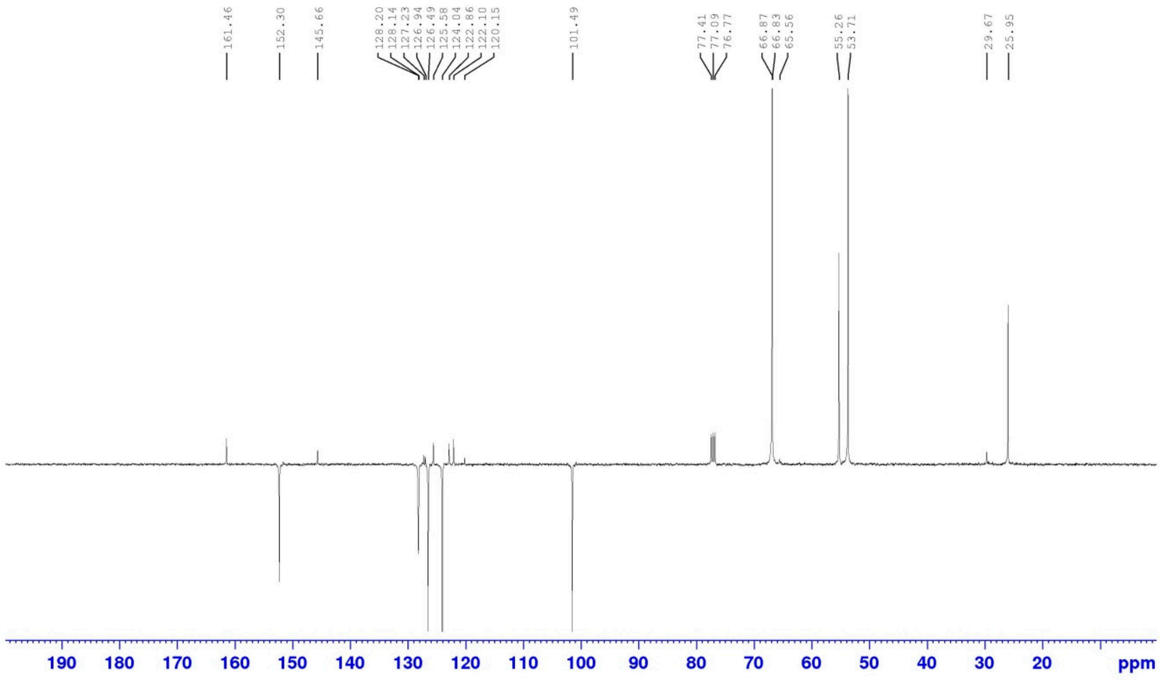

1H NMR Compound 18g
CDCl3

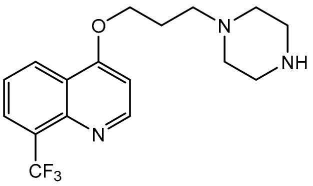


**18g**

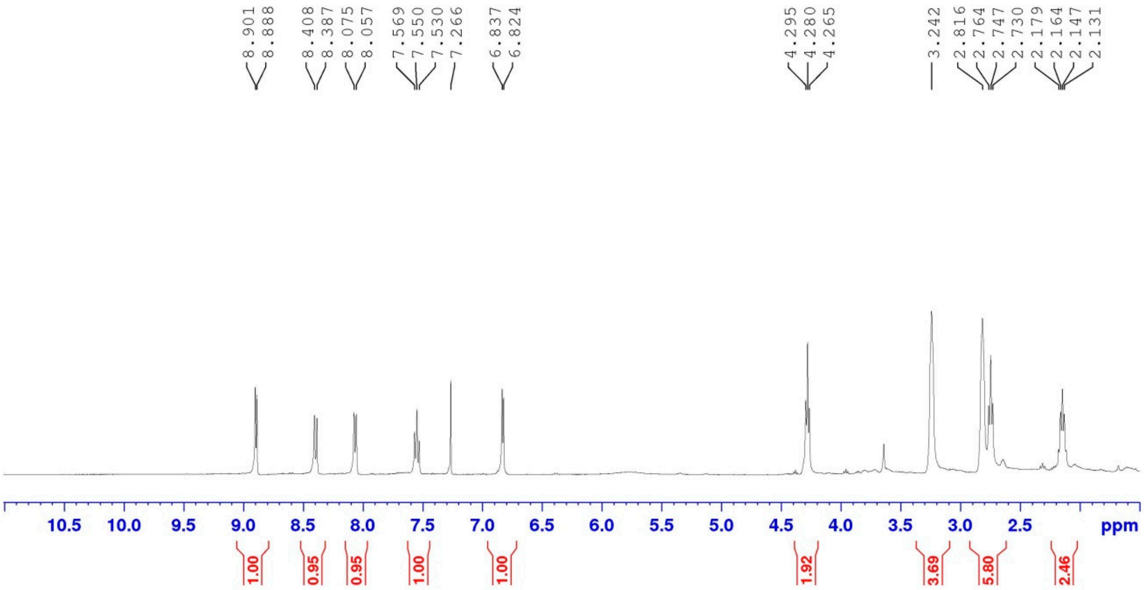


13C NMR Compound 18g
CDCl3

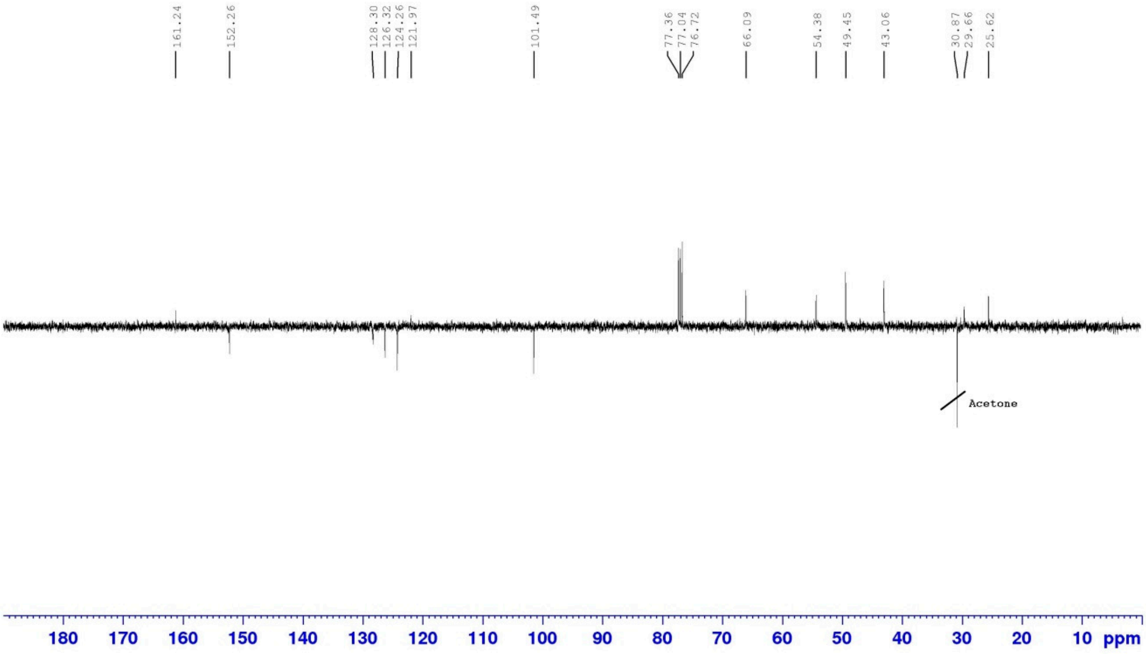

## 7. Elementary Analysis

| | Calculated | | | Found | | |
|---|---|---|---|---|---|---|
| **Comp** | **%C** | **%H** | **%N** | **%C** | **%H** | **%N** |
| **2** | 49.12 | 5.30 | 8.18 | 49.81 | 5.32 | 8.21 |
| **3** | 48.64 | 4.08 | 9.45 | 48.83 | 4.10 | 9.49 |
| **4a** | 59.06 | 4.70 | 7.25 | 59.29 | 4.72 | 7.28 |
| **4b** | 56.75 | 4.54 | 6.30 | 56.52 | 4.52 | 6.27 |
| **5a** | 56.98 | 3.94 | 7.82 | 56.75 | 3.92 | 7.79 |
| **5b** | 53.73 | 3.51 | 6.96 | 53.94 | 3.52 | 6.99 |
| **6** | 63.23 | 4.90 | 3.78 | 63.48 | 4.92 | 3.79 |
| **7** | 60.89 | 3.91 | 4.18 | 60.65 | 3.89 | 4.16 |
| **9b** | 56.32 | 7.09 | 6.57 | 56.54 | 7.11 | 6.59 |
| **9c** | 64.35 | 5.79 | 5.36 | 64.60 | 5.81 | 5.38 |
| **10b** | 56.83 | 6.36 | 7.36 | 56.60 | 6.33 | 7.33 |
| **11a** | 62.43 | 5.92 | 6.33 | 62.68 | 5.94 | 6.35 |
| **11b** | 59.99 | 5.64 | 5.60 | 59.75 | 5.62 | 5.58 |
| **11c** | 52.96 | 5.22 | 8.06 | 52.75 | 5.20 | 8.03 |
| **11d** | 63.81 | 6.43 | 5.95 | 63.55 | 6.40 | 5.92 |
| **11e** | 61.35 | 6.10 | 5.30 | 61.59 | 6.12 | 5.32 |
| **11f** | 54.63 | 5.68 | 7.64 | 54.85 | 5.70 | 7.67 |
| **11g** | 64.44 | 6.66 | 5.78 | 64.70 | 6.68 | 5.80 |
| **12a** | 60.86 | 5.35 | 6.76 | 60.65 | 5.37 | 6.79 |
| **12b** | 57.63 | 4.84 | 6.11 | 57.40 | 4.82 | 6.08 |
| **12c** | 51.11 | 4.70 | 8.51 | 51.31 | 4.72 | 8.54 |
| **12d** | 62.43 | 5.92 | 6.33 | 62.68 | 5.94 | 6.35 |
| **12e** | 59.25 | 5.39 | 5.76 | 59.49 | 5.41 | 5.78 |
| **12f** | 52.96 | 5.22 | 8.06 | 52.75 | 5.20 | 8.03 |
| **12g** | 63.14 | 6.18 | 6.14 | 63.39 | 6.20 | 6.16 |
| **15** | 41.82 | 2.51 | 3.48 | 41.65 | 2.50 | 3.47 |
| **17a** | 54.44 | 5.36 | 8.28 | 54.66 | 5.38 | 8.31 |
| **17b** | 54.16 | 5.02 | 9.97 | 54.38 | 5.04 | 10.00 |
| **17c** | 56.16 | 4.96 | 6.89 | 56.38 | 4.98 | 6.91 |

| | | | | | | |
|---|---|---|---|---|---|---|
| **17d** | 54.82 | 5.11 | 7.10 | 54.60 | 5.09 | 7.07 |
| **17e** | 52.95 | 4.44 | 6.86 | 52.74 | 4.42 | 6.83 |
| **17f** | 56.25 | 4.95 | 6.25 | 56.47 | 4.97 | 6.27 |
| **17g** | 53.07 | 4.70 | 10.32 | 53.28 | 4.72 | 10.36 |
| **20b** | 54.28 | 5.27 | 13.33 | 54.50 | 5.29 | 13.38 |
| **20c** | 56.29 | 5.22 | 10.37 | 56.51 | 5.24 | 10.41 |
| **16** | 46.73 | 3.32 | 4.19 | 46.91 | 3.33 | 4.21 |
| **18b** | 60.13 | 6.42 | 9.56 | 60.37 | 6.44 | 9.59 |
| **18c** | 61.18 | 6.28 | 11.89 | 61.42 | 6.30 | 11.94 |
| **18d** | 63.89 | 6.26 | 8.28 | 63.63 | 6.23 | 8.25 |
| **18e** | 62.56 | 6.49 | 8.58 | 62.31 | 6.46 | 8.54 |
| **18f** | 59.99 | 5.63 | 8.23 | 59.75 | 5.61 | 8.20 |
| **18g** | 60.17 | 5.94 | 12.38 | 60.41 | 5.96 | 12.43 |

## 8. Validation of electrophysiological approach

(A) Protocol of the whole cell patch-clamp included a series of voltage ramp waveform applications (upper panel). Non-transfected cells did not demonstrate significant background current via plasma membrane (middle panel). Cells, expressing Panx1, responded with microscopic current as shown in bottom panel. Our observations indicate that current density via transfected cells membrane at 80 mV is 299±29 pA/pF whereas non-transfected cells developed background current 24±3 pA/pF. In the experiments with drugs, we subtracted the background current to present mPanx1-depended channel activity. (B) I-V characteristic of a typical mPanx1 expressing cell before (black) and after (red) application of $LaCl_3$. (C) effect of cabenoxolone on Panx1-dependent current. CBX was applied and washed out during a continuous series of voltage ramps. (D) Inhibitory effect of cabenoxolone was measured at 80 mV and dose dependent relationship is shown on the graph. For reference, effect of 0.5 mM $LaCl_3$ depicted in red.

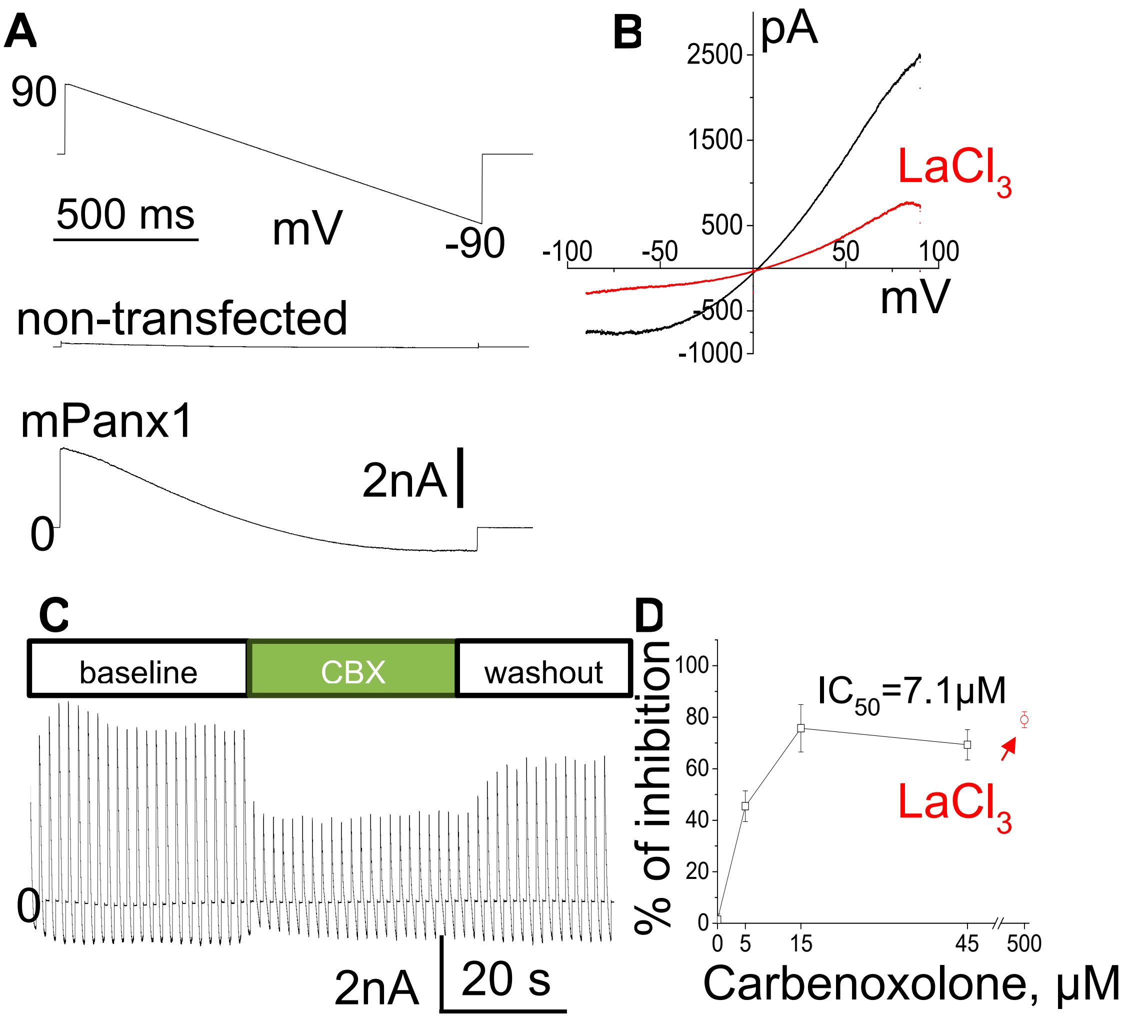

Supplement: Supplementary file 1 [file molecules-30-02171-s001.zip › molecules-3606919-supplementary.pdf]
